# Supplementary material for: Genome assemblies of 11 bamboo species highlight diversification induced by dynamic subgenome dominance
Source: Nat Genet. 2024 Mar 15;56(4):710–20. doi: 10.1038/s41588-024-01683-0 (PMC11018529; doi:10.1038/s41588-024-01683-0)
Supplement: Supplementary file 1 — Supplementary Methods, Supplementary Texts, Supplementary Figs. 1–35 and Supplementary Tables 1, 3–4, 11, 15–16, 18–21, 26–27, 29–30 and 33. [file 41588_2024_1683_MOESM1_ESM.pdf]

nature genetics

Article

https://doi.org/10.1038/s41588-024-01683-0

Genome assemblies of 11 bamboo species highlight diversification induced by dynamic subgenome dominance

In the format provided by the authors and unedited

This PDF file includes:

Supplementary Methods

Supplementary Texts

Supplementary Figs. 1−35

Supplementary Tables 1, 3−4, 11, 15−16, 18−21, 26−27, 29−30, 33

(Supplementary Tables 2, 5−10, 12−14, 17, 22−25, 28, 31−32, 34 are provided in a separate excel document)

Supplementary Methods

Plant materials, sequencing and assembly

Eleven bamboo species of *Ampelocalamus luodianensis*, *Bonia amplexicaulis*, *Dendrocalamus sinicus*, *Guadua angustifolia*, *Hsuehochloa calcarea*, *Melocanna baccifera*, *Olyra latifolia*, *Otatea glauca*, *Phyllostachys edulis*, *Raddia guianensis* and *Rhipidocladum racemiflorum* were sampled for sequencing. Among them, plant materials of *B. amplexicaulis* (Longzhou County, Guangxi, China) and *D. sinicus* (Cangyuan County, Yunnan, China) were collected in the field, and the remaining species were cultivated in Kunming Institute of Botany and Xishuangbanna Tropical Botanical Garden, Chinese Academy of Sciences (CAS). Fresh tissues of the foliage leaf blade, foliage leaf sheath, branch bud, culm leaf sheath, shoot internode, rhizome, root, caryopsis and inflorescence were sampled for RNA extraction and transcriptome sequencing. Shoot tissues in *D. sinicus* were collected at four defined stages of growth as described in Methods. Inflorescence tissues at three developmental stages, including primary spikelet, pre-anthesis spikelet and anthesis spikelet, were collected for *Ra. guianensis*, *A. luodianensis*, *P. edulis*, *Rh. racemiflorum*, *B. amplexicaulis* and *D. sinicus*. At least two and, in most cases, three biological replicates were collected for each tissue. Sequencing libraries of RNA-seq were prepared with 250-350-bp insert size and sequenced on the Illumina NovaSeq or DNBSEQ-T7 platform.

Short-read sequencing was performed on the Illumina NovaSeq or HiSeq 2500 platform. For Oxford Nanopore Technology (ONT) sequencing, DNA libraries were constructed with a standard ONT protocol as per manufacturers' instructions, and long-read sequencing was performed on ONT's PromethION sequencer. Hi-C sequencing libraries were prepared by BioMarker (Beijing, China) or NextOmics Technologies Company (Wuhan, China) following a published protocol[44], and sequenced on the Illumina NovaSeq or HiSeq 2500 platform. The pseudo-chromosomes were built by LACHESIS software[52] with 'CLUSTER_MIN_RE_SITES = 138 or 100,

CLUSTER_MAX_LINK_DENSITY = 2 or 2.5, ORDER_MIN_N_RES_IN_TRUN = 148 or 60, ORDER_MIN_N_RES_IN_SHREDS = 126 or 60'.

Phylogenetic analysis and divergence time estimation

We filtered the 456-gene data set by: (1) genes affected by putative gene conversion (e.g., gene copies from different subgenomes within the same species/clade clustered together in the gene tree), and (2) a >10 Robinson-Foulds distance of the gene tree relative to the ASTRAL species tree. The 456 gene trees were first collapsed by Newick utilities (v1.6.0)[82] for nodes with <10% bootstrap support and then analyzed by ASTRAL (v5.6.3)[80] to infer the species tree (-i <gene trees> -t 3). The Robinson-Foulds distance between each gene tree and the inferred species tree was calculated by the ipyrad analysis toolkit (v0.9.74)[81]. Meanwhile, a larger data set of 2,675 "perfect-copy" syntenic genes was generated from four species whose subgenomes were sampled, i.e., one representative HB of *Ol. latifolia*, two WBs (*H. calcarea* and *M. baccifera*) with each subgenome sampled, and rice as the outgroup. After filtering by ≥50% average bootstrap value of gene tree, 2,021 genes were retained for ML gene tree and coalescent-based tree reconstruction.

The plastomes of 11 bamboos were assembled with GetOrganelle (v1.7.1)[117] using a range of *k*-mers (65, 75, 95, 105, 115 and 125) from the short sequencing reads or downloaded from NCBI (NC_024165.1, NC_028631.1 and NC_029749.1). With rice (NC_001320.1) as outgroup, the 12 plastome sequences were first aligned using MAFFT (v7.471)[74] and subsequently trimmed using trimAl (v1.4)[118] with default parameters. A phylogenetic tree was inferred using RAxML (v8.2.12)[76] under the GTRGAMMA model with 1000 replicates and searching for the best-scoring tree.

MCMCTREE in the PAML (v4.9) package has been used to estimate divergence times in genomic studies with a few taxa and large-scale molecular data sets[109]. Four-fold degenerate sites of the concatenated 430-gene data set were used for analysis with parameters 'model = 4, burnin = 200,000, sampfreq = 100, nsample = 20,000'. The divergence time of rice and the bamboo subfamilies (48.6 Ma to 54 Ma) was used for

secondary calibration based on previous studies[38,119]. Two parallel runs were performed with concordant results.

Ancestral karyotype reconstruction

A total of 183 conserved syntenic blocks were identified by DRIMM-Synteny[89] for analysis. Three ancestral diploid bamboo karyotypes (i.e., ABK-H, ABK-C and ABK-X), the ancestral woody bamboo karyotype (ABK-W) and the ancestral bamboo karyotype (ABK) were reconstructed using the IAGS program[90] with the GMP model iteratively; that is, ABK-H was inferred using the *Ol. latifolia* and *Ra. guianensis* genomes with the rice genome as the outgroup, ABK-C was inferred using the C subgenome of *A. luodianensis* and *M. baccifera* with the B subgenome of *M. baccifera* as the outgroup, ABK-X, which represented the diploid progenitor shared by the A and D subgenome lineages, was reconstructed from the A subgenome of *M. baccifera* and the D subgenome of *A. luodianensis* with the B subgenome of the *M. baccifera* genome as the outgroup, and ABK-W was inferred from ABK-B and ABK-C with the rice genome as the outgroup. Finally, ABK was inferred from ABK-H and ABK-W with the rice genome as the outgroup.

Inference of gene families and homoeologous groups

Five representative grass species [rice, sorghum, *Oropetium thomaeum* (phytozome V12), *Brachypodium distachyon* (Gramene V60) and *Triticum urartu* (http://gigadb.org/dataset/100050)], together with the 11 bamboo genomes, were selected for inferring gene families and homoeologous groups. In addition to the individual genomes, subgenomes of WBs were also treated as operational taxonomic unit. Protein sequences of genes were compared using the DIAMOND method (v2.1.8)[120] in OrthoFinder (v2.5.2)[77] to infer gene families. Groups of genes identified between the two subgenomes (tetraploids) or among the three subgenomes (hexaploids) within one species were classified as homoeologs. Each homoeologous group was assigned a theoretical and an actual cardinality, based on the number of homoeologs identified in each subgenome.

For gene family expansion and contraction analysis, a user-specified divergence

time tree with 11 bamboos and five grasses was obtained by r8s (v1.8.1)[121]. Families with $P < 0.05$ were considered as significantly expanded or contracted. Different types of gene duplication, such as whole-genome duplication (WGD), tandem duplication (TD), proximal duplication (less than 10 gene distances on the same chromosome: PD) or transposed duplication (transposed gene duplications: TRD), were detected using DupGen_finder[122] with default parameters for the expanded gene families. We also identified expanded and contracted gene families at the subgenome level as described above but only with rice as the outgroup. Significantly overrepresented Gene Ontology (GO) and Kyoto Encyclopedia of Genes and Genomes (KEGG) were identified using the R package 'clusterProfiler'[123].

Expression divergence and bias between subgenomes

To examine the breadth of gene expression across tissues, we used the Tau specificity index[124]. The median $\log_2$(TPM) values were calculated for each tissue of all expressed genes (pooling samples from biological replicates to get a single average value). These values were quantile-normalized, and Tau was calculated for each gene in each tissue using the tispec R package (https://rdrr.io/github/roonysgalbi/tispec).

For the analysis of subgenome bias of expression in hexaploids, the normalization of relative expression levels of the A, B and C subgenomes following Ramírez-González et al. (2018)[32] was conducted by focusing exclusively on the 1:1:1 gene triads in PWBs. We defined a triad as expressed when the sum of the A, B and C subgenome homoeologs had TPM> 0.5. This allowed us to include triads in which, for example, only a single homoeolog was expressed, and which could later be classified as a dominant triad. To standardize the relative expression of each homoeolog across the triad, we normalized the absolute TPM for each gene within the triad as follows:

$$\text{Expression}_A = \frac{\text{TPM(A)}}{\text{TPM(A)+TPM(B)+TPM(C)}}$$

$$\text{Expression}_B = \frac{\text{TPM(B)}}{\text{TPM(A)+TPM(B)+TPM(C)}}$$

$$\text{Expression}_C = \frac{\text{TPM(C)}}{\text{TPM(A)+TPM(B)+TPM(C)}}$$

where A, B and C represent the gene corresponding to the A, B and C homoeologs in the triad. The normalized expression was calculated for each tissue and for the average across all tissues in which a gene was expressed. For the homoeologous expression bias categories in tissues, the leaf blade collected on non-flowering branch at reproductive stage in *D. sinicus* was excluded for extremely low proportion (29.2%) of balanced triads[9,32] in subsequent analyses. The values of the relative contributions of each subgenome per triad were used to plot the ternary diagrams using the R package ggtern[105].

Identifying PSGs and tissue-specific expressed genes

For the subgenome-based approach in identifying positively selected genes (PSGs), we used rice and two HB species as outgroups and assembled nine data sets at the subgenome level: (I) PWB-subA, (Osa, ((Ola, Rgu), (MbaA, (DsiA, BamA)) #1)); (Ⅱ) PWB-subB, (Osa, ((Ola, Rgu), (MbaB, (DsiB, BamB)) #1)); (Ⅲ) PWB-subC, (Osa, ((Ola, Rgu), (MbaC, (DsiC, BamC)) #1)); (Ⅳ) NWB_subB, (Osa, ((Ola, Rgu), (RhiB, (GanB, OglB)) #1)); (Ⅴ) NWB_subC, (Osa, ((Ola, Rgu), (RraC, (OglC, GanC)) #1)); (Ⅵ) TWB_subC, (Osa, ((Ola, Rgu), (AluC, (PedC, HcaC)) #1)); (Ⅶ) TWB_subD, (Osa, ((Ola, Rgu), (AluD, (PedD, HcaD)) #1)); (Ⅷ) the subB lineage, (Osa, ((((DsiB, BamB), MbaB), ((GanB, OglB), RraB)) #1, (Rgu, Ola))); (Ⅸ) the subC lineage, (Osa, ((Ola, Rgu), (((HcaC, PedC), AluC), ((MbaC, (DsiC, BamC)), (RraC, (OglC, GanC)))) #1)). We identified 5,939, 5,476, 5,452, 6,193, 6,869, 6,978, 6,625, 3,228 and 2,298 one-to-one orthologs for these nine data sets, respectively, using OrthoFinder (v2.5.2)[77]. The protein sequences were aligned with MAFFT (v7.475)[74] and trimmed with trimAl[118] using the 'automated1' option. The coding sequences were aligned by PAL2NAL (v14)[75] from the corresponding aligned protein sequences.

Positive selection signals on genes along the common branch leading to the subgenome lineage of WBs as labeled above were detected using the branch-site model by the Codeml program in the PAML (v4.8) package[109]. A likelihood ratio test (LRT) was conducted to compare a model that allowed sites to be under positive selection on the foreground branch with the null model in which sites could evolve either neutrally

or under purifying selection. The P value was determined by a Chi-square test with a cutoff of <0.05 for positive selection.

To determine the PSGs in clades of WBs, we defined the following: PWB = PWB-subA ∪ PWB-subB ∪ PWB-subC; NWB = NWB_subB ∪ NWB_subC; and TWB = TWB_subC ∪ TWB_subD. As a result, 1,190, 879 and 981 genes were found as PSGs in the PWBs, NWBs and TWBs, respectively. PSGs shared by all three WB clades were defined as the subC lineage ∪ (the subB lineage ∩ TWB) ∪ (PWB ∩ TWB ∩ NWB).

To identify specifically expressed genes, we took *D. sinicus* and *P. edulis* for analyses. Pairwise comparison between tissues were made by DESeq2 (v1.14.1) package[104] in R with the threshold of up- or down-regulated >2 fold with an FDR adjusted P value <0.05. Genes were considered as having tissue-specific expression if up-regulated in one tissue and down-regulated in another.

We also identified vegetative and reproductive stage-specific expressed genes using the expression data from the leaf blade of *Ra. guianensis*, *P. edulis*, *Rh. racemiflorum*, *B. amplexicaulis* and *D. sinicus* for analyses of nonsynonymous substitution (*Ka*) and synonymous substitution (*Ks*) rates. Genes expressed with TPM ≥ 1 at the vegetative stage while TPM < 1 at the reproductive stage were vegetative-specific, defined as vegetative-related genes, and vice versa for the definition of reproductive-related genes. The *Ka/Ks* values were also calculated for all sampled HBs and WBs based on genome-wide syntenic orthologous gene pairs between the rice and bamboo genomes. Orthologous gene pairs were aligned using the parallel tool ParaAT (v2.0)[125] in MAFFT (v7.475)[74]. The *Ka/Ks* values were estimated for gene pairs based on the YN00 model by KaKs-Calculator (v2.0)[110].

Growth pattern of *D. sinicus* shoot and anatomical observation

During the field investigation, a total of 208 healthy shoots from 38 clumps of *D. sinicus* were randomly selected for measurement at 9 a.m. each day. Based on the growth curve (Extended Data Fig. 8a), we defined four stages (ST1 to ST4) for fast growth of shoot. For anatomical observation, the 10th internode tissue of *D. sinicus* was fixed in FAA for at least 48 hours, dehydrated in an ethanol series, infiltrated with xylene and embedded in paraffin. Longitudinal and transverse sections of 5 μm in

thickness were obtained by a slicer. To visualize the degree of lignification and the anatomical structure of samples, sections were stained with 1% safranin-O for 1.5 hour, washed with distilled water, discolored in an ethanol series and then counterstained with 0.5% fast green for 1 minute, followed by sealing with neutral balsam. Lignified zones were stained in red.

Supplementary Texts

Subgenome identification

For “perfect-copy” syntenic gene blocks, 29 phylogenetic trees were reconstructed; however, out of 41 “low-copy” syntenic gene blocks, six blocks failed to identify orthogroups across all 12 species, and were thus unable to be used to infer phylogeny, resulting in only 35 phylogenetic trees (Supplementary Figs. 7 and 8). Based on the topologies of these trees, we named the subgenome shared by all WBs as C, the subgenome shared by only tropical woody bamboos as B, the remaining subgenomes within PWBs and TWBs as A and D, respectively, and the HB genome as H following our previous work[17].

We found divergence of sequence similarity values among different subgenome pairs (Extended Data Fig. 2b) and the same kinds of subgenomes from different polyploid species clustered together. High similarity values were shown for subgenomes A and D. We then removed those self-comparisons, such as AluC versus AluC, and the average sequence similarities of retained pairs revealed that (Extended Data Fig. 2c), in all comparisons, the similarity between the same subgenome across different polyploid species (85.3%−89.5%) was significantly higher than that for inter-subgenomes (73.7%−82.1%) with $P < 0.001$. The D-D and A-A comparisons from different species showed the highest average similarity (89.5%), followed by C-C (85.7%) and then B-B (85.3%). For comparisons among different subgenomes, A-D, A-C and C-D represented significantly higher similarity (82%) than the others ($P < 0.001$). The B-D (81.1%), A-B (81.1%) and B-C (80.8%) comparisons showed lower similarities, and the similarities between the H genome and the subgenomes of WBs were significantly lower (73.6%−73.7%) when compared with other inter-subgenome pairs within WBs ($P < 0.001$), suggesting the high sequence differentiation between HBs and WBs.

Phylogenetic analysis

Multiple chromosome-level genomes from all clades of bamboos provided an

opportunity to re-assess their origins and polyploidization. Three tree-building strategies of 430-gene dataset consistently resolved WBs as monophyletic (Extended Data Fig. 3a,b). Most nodes within WBs were well-resolved with high support values, suggesting B progenitor diverged firstly, and either A or D progenitor were sisters with C progenitor, whereas low support and conflicting relationships of the A and D progenitors between the concatenation four-fold degenerate site tree and the other two were revealed.

As mentioned in the main text, extensive gene tree discordance (Fig. 2a) were observed within both 430 and 2,021 gene datasets, thus we further adopted several analyses for discordance quantification and visualizations. Results of phyparts analyses for both two datasets revealed similar patterns (Extended Data Fig. 3c). Most nodes of the ASTRAL tree topology were supported by the majority of individual gene trees, while only 75 (17.4%) and 80 (18.6%) genes out of the 430 genes and 391 (19.3%) and 318 (15.7%) out of the 2,021 genes supported the placement of the A and D subgenome lineages in the coalescent-based tree, respectively. More strikingly, we found only 196 (46%) out of 430 and 950 (47%) out of 2,021 genes supported the monophyly of WBs.

We then calculated the distributions of each observed topology (Supplementary Tables 7 and 9). We first examined the frequency of the plastid-like topology, that is, the C/D subgenomes of TWB diverged first, followed by H and other subgenomes, which appeared in 6% (27) and 8% (168) of the nuclear gene trees from 430 and 2,021 genes. The majority (57%, 246/1,146) of gene topologies from the 430 and 2,021 genes matched the H(B,C) topology of the bifurcating tree, with 25% (105/509) and 18% (79/366) supporting the B(H,C) and C(H,B) topologies, respectively. In addition, 17% (74) and 16% (320) of the nuclear gene trees from 430 and 2,021 genes supported a close relationship between the A and H progenitors. We found that the B and C progenitors are more closely related to A or D individually than to each other, with B(A,C) (48%/46%, 206/919) and B(D,C) (46%/42%, 199/855) topologies being the most common. The frequencies of the other two minor alternative topologies were unequal, with 28%/29% (121/592) and 31%/34% (132/677) supporting the C(A,B) and

C(D,B) topologies, and 24%/25% (103/510) and 23%/24% (99/489) supporting the A(B,C) and D(B,C) topologies (Fig. 2b and Supplementary Fig. 11).

ILS, hybridization and introgression analyses

The estimated theta values (Supplementary Fig. 10) ranged from 0.0027 (the split of DsiC and BamC) to 0.0575 (the divergence of herbaceous and woody bamboos), indicating a low ILS level for the whole tree (0.01 and 0.1 for low and high ILS, respectively)[84]. The ILS level of deep nodes, i.e., the divergence among different subgenomes, was observed to be higher (average 0.0257) than shallow ones (average 0.0045), which is consistent with the short internodes connecting different subgenome lineages.

The inferred phylogenetic networks for both gene data sets (430 and 2,021 genes) revealed a total of seven hybridization events (Extended Data Fig. 3e,f and Supplementary Fig. 12), indicating frequent gene flow among bamboo ancestor lineages. Among these, two main hybridization scenarios, supporting ancient hybridization between B and C progenitors and between H and A progenitors, were consistently recovered with the highest log probabilities. Most of the other hybridization scenarios also occurred before species diversification but with significantly skewed inheritance probabilities (such as 0.9 and 0.1), suggesting introgression. These introgression events were also revealed by QuIBL and HyDe analyses (Extended Data Fig. 3d and Supplementary Table 10). QuIBL analysis indicated that 27% of the tested triplets showed significant evidence for introgression (ΔBIC< -10). Moreover, introgression levels were higher among subgenomes and deeper nodes in the phylogenetic tree, such as between H and A progenitors. Similarly, HyDe detected introgression between H and WB progenitors, as well as between different WB ancestors (such as B and D). Based on results of our comprehensive analyses, we expect hybridization/introgression to be the main factor explaining most of the observed phylogenetic discordance.

Inferring the origin and evolution of major bamboo clades

Taking the evidence from sequence similarity, gene tree topology distribution, cytonuclear discordance, ILS analysis, phylogenetic networks and introgression analyses together, we propose a refined model to decipher the reticulate relationships among different bamboo progenitors (Fig. 2d). In this model, hybridization was considered as the major factor underlying the reticulate evolution with introgression playing a minor role. The differentiation of the herbaceous and woody lineages that occurred early in the evolution of bamboo was supported by their highest sequence differentiation and most gene trees, followed by the divergence of the woody ancestors initially into two (B and C) diploid progenitors, which was supported from their low sequence similarity and results of phylogenetic inference. The other two diploid progenitors of WBs, A and D, likely originated from an extinct diploid ancestor, which derived through homoploid hybrid speciation between the B and C progenitors. The higher similarity between the A and D progenitors compared with other inter-subgenome comparisons and the similar origins supported by phylogenetic networks indicate their close affinity; however, a non-sister relationship of A and D progenitors in most gene trees and their significantly lower sequence similarity than that of the intra-subgenome comparisons suggested that A and D have already differentiated to a certain extent and are thus invoked as distinct subgenomes in our study.

After the early divergence between the herbaceous and woody lineages, introgression between the H and A progenitors was detected based on the results of gene tree topology distribution, cytonuclear discordance, phylogenetic networks and introgression analyses. Moreover, we also observed gene flow between the H progenitor and other diploid ancestors of WBs, such as the D progenitor, indicating that the evolutionary history between HBs and WBs may be more complex than previously thought[17]. Based on the distribution of gene tree topologies, except for B and C lineages, gene flow between other diploid ancestors of WBs may also occur, indicating the possibility of the existence of other scenarios, such as those proposed based on TE evidence in Chalopin et al.[16].

Estimation of divergence times

Based on the results of age estimation (Supplementary Fig. 14), the time range of the major evolutionary events within bamboos is inferred as follows: the time range of hybridization event between B and C progenitors leading to A and D progenitors (30.17–32.04 Ma) was inferred to have occurred after the differentiation of the B and C progenitors ~32.04 Ma and no later than the divergence between the A and D progenitors ~30.17 Ma; the hybridization between the B and C1 progenitors happened around ~20.58 Ma and was inferred to have occurred after the divergence of the C1 (in the tropical WBs) and C2 (in the TWBs) progenitors ~20.59 Ma and no later than the species divergence within the B progenitors ~20.57 Ma; the hybridization event between the C2 and D progenitors from 20.57 Ma to 12.04 Ma was inferred to have occurred after the divergence of the C2 from C1 progenitors ~20.57 Ma and earlier than the species divergence within TWBs of ~12.04 Ma; the hexaploidization from a hybridization between an A progenitor and allotetraploid NWBs, which occurred no later than 13.43 Ma, was inferred to have occurred after the divergence of the NWBs and PWBs around 18.56 Ma, and earlier than the species divergence within PWBs of ~13.43 Ma.

Ancestral karyotype reconstruction

ABK-B was represented using the B subgenome of *M. baccifera* due to the extremely conserved chromosomal structure of the B subgenome. Ancestral bamboo karyotypes of six progenitors were reconstructed with high estimated accuracy (from 92.91% for ABK-C to 99.47% for ABK-X) and low completely rearranged endpoint (CRE) ratio (from 1.64% for ABK-X to 16.94% for ABK-C), indicating the high accuracy. Except for ABK-H and ABK-C, ancestral bamboo karyotypes of the other progenitors, ABK-B, ABK-X, ABK-W and ABK, maintain stunning stability with the ancestral grass karyotype (AGK) in evolution (Fig. 3a). ABK-H was reconstructed as 11 chromosomes, with a nested chromosome fusion (NCF)[126] between chr12 and chr10 of the rice genome representing the AGK (Extended Data Fig. 2d). We did find this event occurring in both sequenced herbaceous bamboos, but these two species were sampled from the same subtribe Olyrinae; increased samplings of genomes from the remaining two subtribes

of Buergersiochloinae and Parianinae to test these results remain to be further explored. ABK-C was reconstructed as 12 chromosomes, and only three chromosomes (chrs2, 4, 7) were preserved in the ancestral state of AGK.

Identification of chromosomal rearrangements and inversions

An NCF event in HBs was responsible for their chromosome number reduction from 12 to 11 as demonstrated above. Interestingly, we found a shared one-way translocation event that occurred in all three TWB species, in which a large segment of chr2C transferred to chr9D and formed a mosaic chromosome by chromosomal fusion (Extended Data Fig. 2d). The chromosome evolution scenarios are more complex in the tropical clades, 35 chromosomes of 12A+12B+11C in all PWBs species, and for NWBs, 24 chromosomes of 12B+11C and a possible B chromosome ('Y') in *Rh. racemiflorum*, 23 chromosomes of 12B+11C in *G. angustifolia*, and possible 24 chromosomes of 12B+12C in *Ot. glauca*. Our syntenic analyses showed that five fission and five fusion events involving chrs 3, 6, 11, and 12 accounted for the chromosome number reduction from 12 to 11 of subgenome C in tropical WBs. We speculated that this reduction of chromosome number in the C subgenome has occurred in the ancestors of the tropical clades, and that chr11 of the C subgenome in *Ot. glauca* experienced an additional fission event leading to two chromosomes.

To detect putative homoeologous exchanges (HE) between subgenomes within each WB species, we analyzed a set of 25,249 orthogroup genes and their corresponding gene trees. Our examination revealed that the occurrence of HEs in each WB genome varied between 0.43% and 1.27% (Supplementary Table 11), which is quite low when compared with the typical mosaic genomes such as allohexaploid oat[28]. Importantly, we observed no evidence of large-scale HEs between homoeologous chromosomes (Supplementary Fig. 17), further indicating only occurrence of small-scale HEs and general subgenome stability.

We observed contrasting rearrangement patterns among different bamboo subgenomes, with most rearrangements occurring in the H and C subgenomes (Fig. 3b

and Supplementary Table 12). A total of 12/5 fission and 13/6 fusion events for *Ol. latifolia* and *Ra. guianensis* were detected, respectively. Among these, only three out of 36 events were shared, indicating largely species-specific rearrangement patterns in the HBs. The C subgenome of tropical WB clades is the most activated, with a total of 24(25) fission and 25 fusion events for each species in comparison to the rice genome. The C subgenome of TWBs also experienced frequent rearrangements, with 12(10) fission and 11(9) fusion events for each species. Unlike the species-specific pattern observed in HB species, a large number of events in the C subgenome were shared by the species within the tropical and temperate clades, respectively. Additionally, different rearrangement patterns were observed for the C subgenomes between tropical and temperate clades, probably due to their independent polyploidizations and subsequent distinct evolutionary processes. Therefore, taking evidence of different rearrangement patterns, independent polyploidization processes and their monophyletic positions into consideration, we divided the C subgenome of tropical and temperate bamboos into C1 and C2 lineages, respectively.

We mapped all the detected inversions to the phylogenetic network and traced the evolutionary history of those shared ones (Fig. 3a). The average percentages of species-specific inversions for HBs, TWBs, NWBs and PWBs was 61%, 32%, 33% and 30%, and for H, A, B, C and D subgenomes were 61%, 35%, 34%, 26% and 36%, respectively. More than half of the inversions within HBs were species-specific, and the C subgenome contained the fewest species-specific inversions within the four woody subgenomes. In total, we reconstructed the evolutionary history of 196 out of 245 inversions shared by two or more species (Supplementary Table 14). Among these, eight inversions occurred in the common ancestor of all bamboos, six occurred in the WBs ancestor, 13 occurred in the HBs ancestor and ten occurred in the C ancestor. Notably, we found eight inversions being shared only by the A and D lineages, consistent with their origin from a common ancestor. The most shared inversions (94, 48%) occurred at nodes before species diversification, likely induced by polyploidization events. We also found a total of 17 out of 245 inversions shared by

HBs and woody subgenomes, suggesting complex evolutionary history between them as documented in phylogenetic analyses above.

Classification of gene families and homoeologous groups among subgenomes

The genes of five grasses and 11 bamboo genomes were clustered into a total of 35,626 gene families. Most genes in subgenomes (50%−77%) are present in homoeologous groups for all WB species. In tetraploids, the numbers of genes in homoeologous groups are very similar between subgenomes, and hence could be considered balanced. In contrast, hexaploids showed a slightly higher number of A-subgenome genes in homoeologous groups, together with less gene loss (Supplementary Fig. 18), which was consistent with its more recent incorporation into the hexaploid genomes by hybridization with the BBCC tetraploid progenitor. About 74% to 85% of homoeologous groups were maintained as 1:1 (B:C or C:D) in tetraploid bamboos while 22% to 25% as 1:1:1 (A:B:C) in hexaploid bamboos. We further checked the microsynteny of the 1:1/1:1:1 homoeologs and identified 10,929, 9,092, 10,808, 7,931, 7,455, 10,648, 3,510, 2,631 and 3,895 homoeologous pairs/triads in synteny between subgenomes within *A. luodianensis*, *H. calcarea*, *P. edulis*, *Rh. racemiflorum*, *G. angustifolia*, *Ot. glauca*, *M. baccifera*, *B. amplexicaulis* and *D. sinicus*, respectively.

The subgenome-specific genes were further defined as those found only in one subgenome but not in its counterpart(s) within the genome of WBs while with (conserved) or without (non-conserved) homoeologs in the other 15 analyzed genomes. In tetraploids, the C subgenome has more conserved subgenome-specific genes compared to the B or D subgenome (8,627, 8,695 and 9,704 of the C subgenome versus 7,515, 6,920 and 8,215 of the D subgenome in *A. luodianensis*, *H. calcarea* and *P. edulis*, respectively; 8,872, 9,208 and 7,713 of the C subgenome versus 8,614, 8,854 and 6,843 of the B subgenome in *Rh. racemiflorum*, *G. angustifolia* and *Ot. Glauca*, respectively), indicating greater gene retention for the C subgenome. In hexaploids, the A subgenome has more conserved subgenome-specific genes (A:B:C=5,084:4,358:3,579, 5,353:4,505:3,892 and 5,471:4,355:3,328 in *M. baccifera*,

B. amplexicaulis and *D. sinicus*, respectively) and the lowest level of individual homoeologs loss (1:1:0/1:0:1 > 0:1:1 of A:B:C) in all three species.

Subgenome expression bias and origin

The global expression patterns across all tissues revealed a significantly lower number of preferentially expressed genes in the B subgenome compared to the A subgenome (up-regulated genes in A versus B: 7,662 versus 7,370, 9,265 versus 9,035, 10,908 versus 10,489 in *M. baccifera*, *B. amplexicaulis* and *D. sinicus*, respectively; $P < 0.01$, Wilcoxon rank-sum test) and the C subgenome (B versus C: 7,349 versus 7,621, 8,867 versus 9,337, and 10,688 versus 10,874; $P < 0.01$, Wilcoxon rank-sum test). We further performed WGCNA analysis and obtained 2,604 (C) and 2,440 (D) hub genes in *P. edulis*, 3,163 (B) and 3,391 (C) in *G. angustifolia*, and 1,387 (A), 1,277 (B) and 1,255 (C) in *D. sinicus* (Supplementary Table 25).

We investigated the origin and evolution of gene expression bias according to the rule of parsimony based on the expression data from vegetative leaf blade. Biased homoeologous pairs/triads that were present in all three species of each clade were defined as ancestral biased pairs/triads. We identified 175, 460 and 16 ancestral biased pairs/triads, accounting for 4.25%, 11.98% and 1.38% of the total shared pairs/triads in TWBs, NWBs and PWBs (Supplementary Table 26), respectively. This indicated that expression bias could form immediately following the polyploidization events, and that the bias in NWBs may evolve faster than TWBs. Moreover, we observed a gradually increase in the percentage of biased homoeologous pairs/triads as species diverged (Extended Data Fig. 6c), reaching an average of 31.64%, 50.30% and 20.63% in TWB, NWB and PWB species, respectively.

In addition, we identified 2,117, 371, 443, 137, 476, 383, 520 and 4,678 tissue-specific genes in inflorescence, reproductive leaf blade, vegetative leaf blade, leaf sheath, root, rhizome, culm sheath and shoot in *D. sinicus*, respectively, and 247, 359, 302, 781, 410, 402 and 4,841 genes in inflorescence, reproductive leaf blade, vegetative leaf blade, vegetative leaf sheath, root, rhizome and shoot in *P. edulis* (Supplementary

Table 27), respectively. Most tissue-specific genes were found in inflorescence and shoot, which is in agreement with our previous study[34].

Evolution of new genes and gene families

We identified 163 new gene families shared by the ancestor of WBs. In fact, many of these new genes were lost after origin during subsequent evolution, leading to varying numbers of retained new genes in extant bamboo species, with 116, 90, 173, 116, 114, 138, 125, 142 and 131 ones in *A. luodianensis*, *H. calcarea*, *P. edulis*, *Rh. racemiflorum*, *G. angustifolia*, *Ot. glauca*, *M. baccifera*, *B. amplexicaulis* and *D. sinicus*, respectively (Supplementary Table 28). The expression profiles across different tissues were scanned for 131 and 173 new genes in *D. sinicus* and *P. edulis*, respectively.

For gene family evolution, 6,800 gene families were identified as expanded while 24 gene families contracted in the ancestor branch leading to WBs. These expanded gene families contain 15,404 to 22,580 genes in different WB species, representing two-fold to three-fold as many as that in HBs and other diploid grasses (Supplementary Table 29). Moreover, 46%−86% of the extra genes resulted from polyploidization, and 1%−3% were associated with tandem duplication in different species (Supplementary Table 30). In addition, there were 262, 69, 302 and 902 gene families expanded in the subgenomes of A, B, C and D, respectively.

Growth pattern of shoot in *D. sinicus* and anatomical observation

Based on the growth curves, the shoot development of *D. sinicus* is initially slow upon unearthing, and the growth rate remains slow when the whole shoot height is under 1 m, with daily increments of less than 5 cm. Subsequently, the growth rate begins to accelerate, with daily increments of about 20 cm, 30 cm and 50 cm when the whole shoot height is under 2 m, 8 m and 10 m, respectively. This rapid growth is the result of simultaneous rapid elongation of multiple internodes, which have similar ‘slow-fast-slow’ growth patterns. We thus defined four stages (ST1, slow-growing stage; ST2, early fast-growing stage; ST3, middle fast-growing stage; and ST4, late fast-growing stage) to collect the 10th internode for anatomical observation, which were fixed in

formalin–acetic acid–alcohol (FAA).

At the ST1 stage, many cellular tissues within the internode were undifferentiated, with only parenchyma and vasculature visible (Extended Data Fig. 8c). Cells in the basic tissue displaced uniform length and distinct nuclei. At the ST2 stage, vascular tissues have been completely differentiated into phloem, protoxylem vessel and metaxylem vessels, and the volume of parenchyma cells increased. At the ST3 stage, the cell wall of the vascular bundle sheath began to thicken, and the nucleus of the parenchyma cells decreased. Parenchyma cells further increased in size and diverged into long and short parenchyma cells. By the ST4 stage, the deposition of lignin on the secondary cell wall of the vascular bundle sheath makes it significantly thickened, and the vessel has been fully connected with the cavity formed by the degradation of the protoxylem. The nucleus of parenchyma cells became almost invisible, and long and short parenchyma cells exhibited a mosaic distribution in the longitudinal direction. As the degree of lignification increased, the cell wall of the vascular bundle sheath also thickened, and together these processes ensured the material transport and mechanical support during the rapid growth of the *D. sinicus* shoot.

There were 13,901, 1,501 and 2,716 differentially expressed genes (DEGs) between ST2 versus ST1, ST3 versus ST2 and ST4 versus ST3 during the rapid growth of the *D. sinicus* shoot, respectively, and 114 of them were enriched in lignin synthesis. These DEGs were grouped into 18 clusters by STEM. The trend of expression of 31 DEGs in a significantly grouped cluster showed an increase from ST1 to ST4, which was consistent with the increase of lignin content in the shoot during rapid growth.

In addition, WGCNA analysis of the total 16,074 DEGs was performed with 17 modules clustered. The module–trait relationships were estimated using the correlation between MEs and traits of growth rate, content of lignin, cellulose and hemicellulose. The brown module with 1,423 DEGs was significantly positively correlated with all four traits (Extended Data Fig. 9a). Intersecting the genes in the brown module and those 31 DEGs in the significantly grouped cluster identified by STEM revealed a shared a hub lignin-related gene, *COMT*.

Supplementary-only references

117. Jin, J.-J. *et al.* GetOrganelle: a fast and versatile toolkit for accurate *de novo* assembly of organelle genomes. *Genome Biol.* **21**, 1−31 (2020).
118. Capella-Gutiérrez, S., Silla-Martínez, J. M. & Gabaldón, T. trimAl: a tool for automated alignment trimming in large-scale phylogenetic analyses. *Bioinformatics* **25**, 1972−1973 (2009).
119. The International *Brachypodium* Initiative. Genome sequencing and analysis of the model grass *Brachypodium distachyon*. *Nature* **463**, 763−768 (2010).
120. Buchfink, B., Reuter, K. & Drost, H.-G. Sensitive protein alignments at tree-of-life scale using DIAMOND. *Nat. Methods* **18**, 366−368 (2021).
121. Sanderson, M. J. r8s: inferring absolute rates of molecular evolution and divergence times in the absence of a molecular clock. *Bioinformatics* **19**, 301−302 (2003).
122. Qiao, X. *et al.* Gene duplication and evolution in recurring polyploidization-diploidization cycles in plants. *Genome Biol.* **20**, 38 (2019).
123. Yu, G., Wang, L.-G., Han, Y. & He, Q.-Y. clusterProfiler: an R package for comparing biological themes among gene clusters. *OMICS: J. Integrative Biol.* **16**, 284−287 (2012).
124. Yanai, I. *et al.* Genome-wide midrange transcription profiles reveal expression level relationships in human tissue specification. *Bioinformatics* **21**, 650−659 (2005).
125. Zhang, Z. *et al.* ParaAT: a parallel tool for constructing multiple protein-coding DNA alignments. *Biochem. Biophys. Res. Commun.* **419**, 779−781 (2012).
126. Lysak, M. A. Celebrating Mendel, McClintock, and Darlington: On end-to-end chromosome fusions and nested chromosome fusions. *Plant Cell* **34**, 2475−2491 (2022).

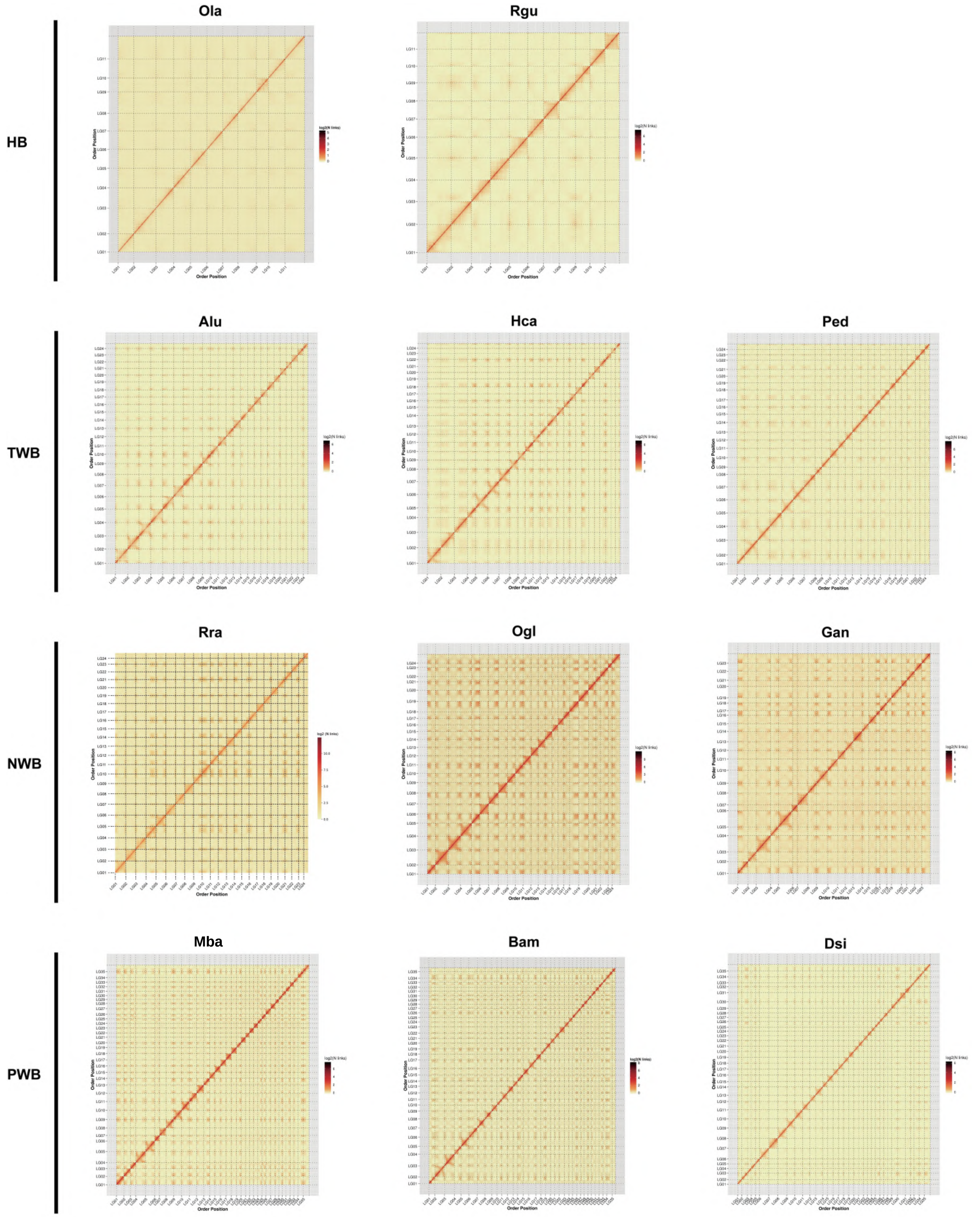

Supplementary Fig. 1. The Hi-C heatmap of 11 pseudochromosome-scale assembled genomes of bamboos. A clear distinction of 11, 23, 24 and 35 groups across the 11 assemblies could be found.

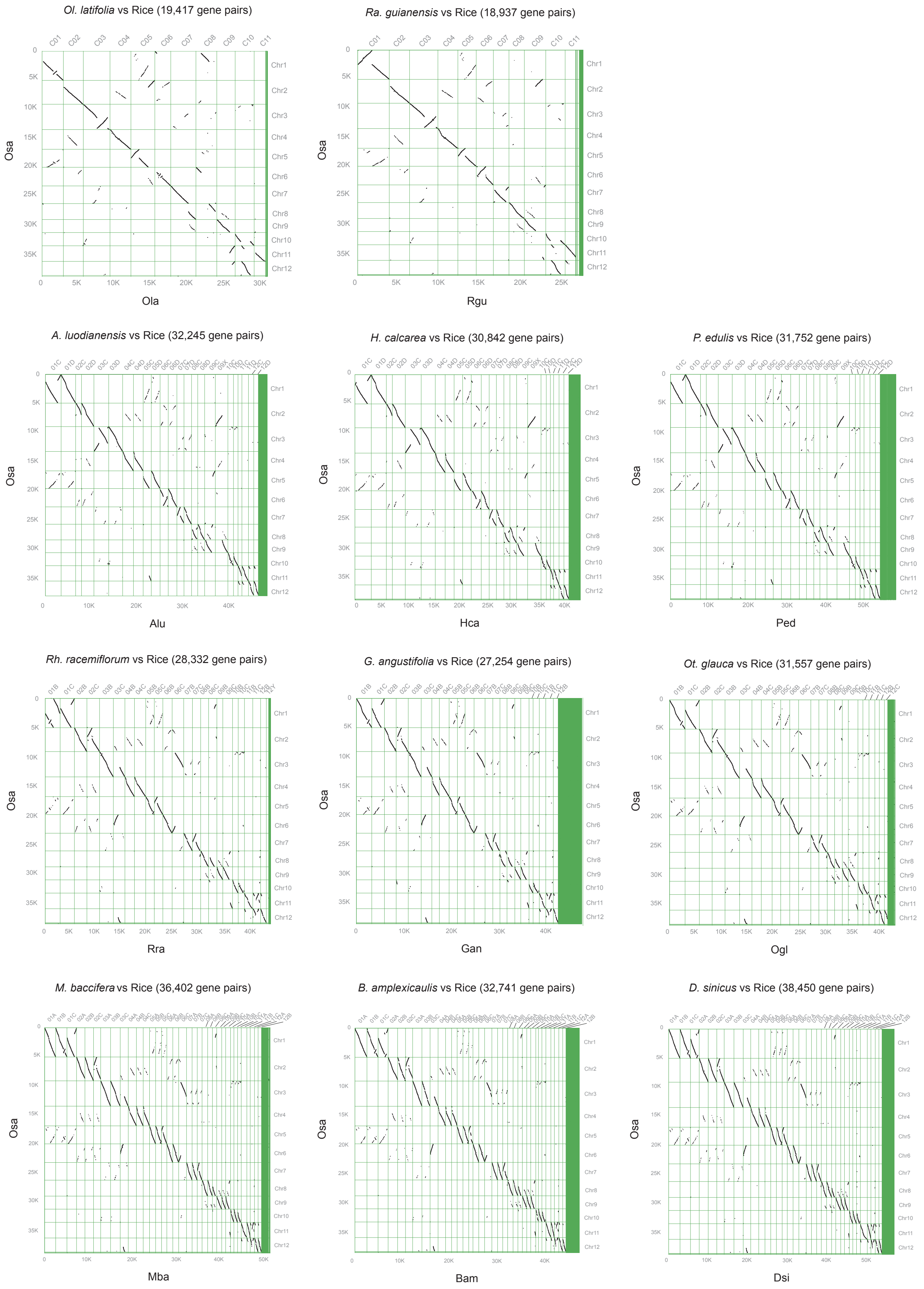

Supplementary Fig. 2. The chromosome-level syntenic dotplots between the rice and bamboo genomes. A general pattern of 1:2:3 between the rice chromosome and the diploid, tetraploid and hexaploid bamboo chromosomes are revealed, respectively.

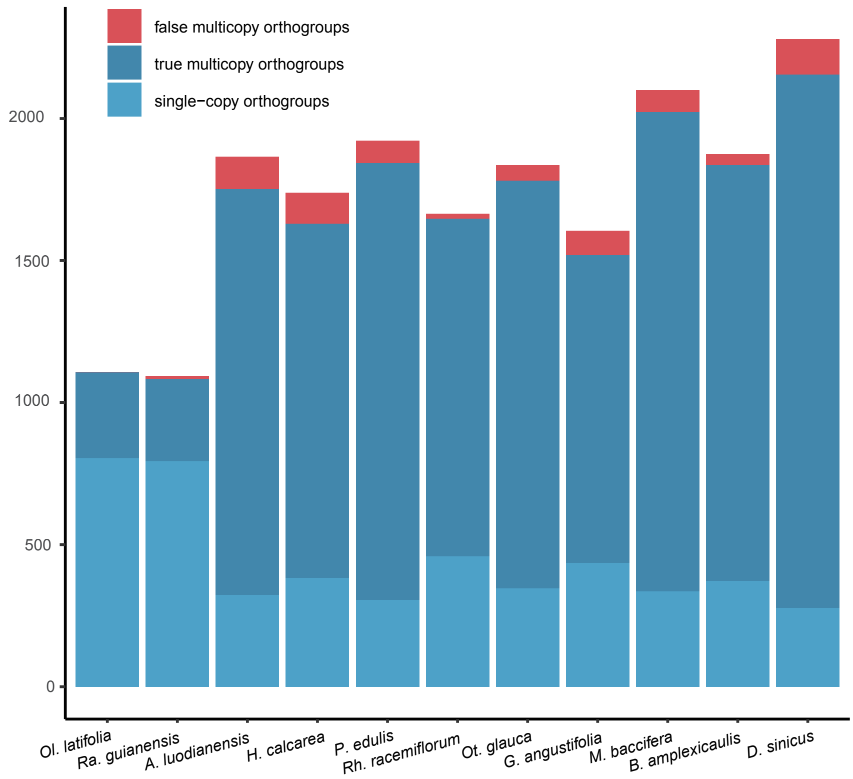

Supplementary Fig. 3. Values of accurately assembled genes (AG) for the 11 assembled bamboo genomes. AG is calculated summing the number of BUSCO genes in single-copy and true multicopy orthogroups.

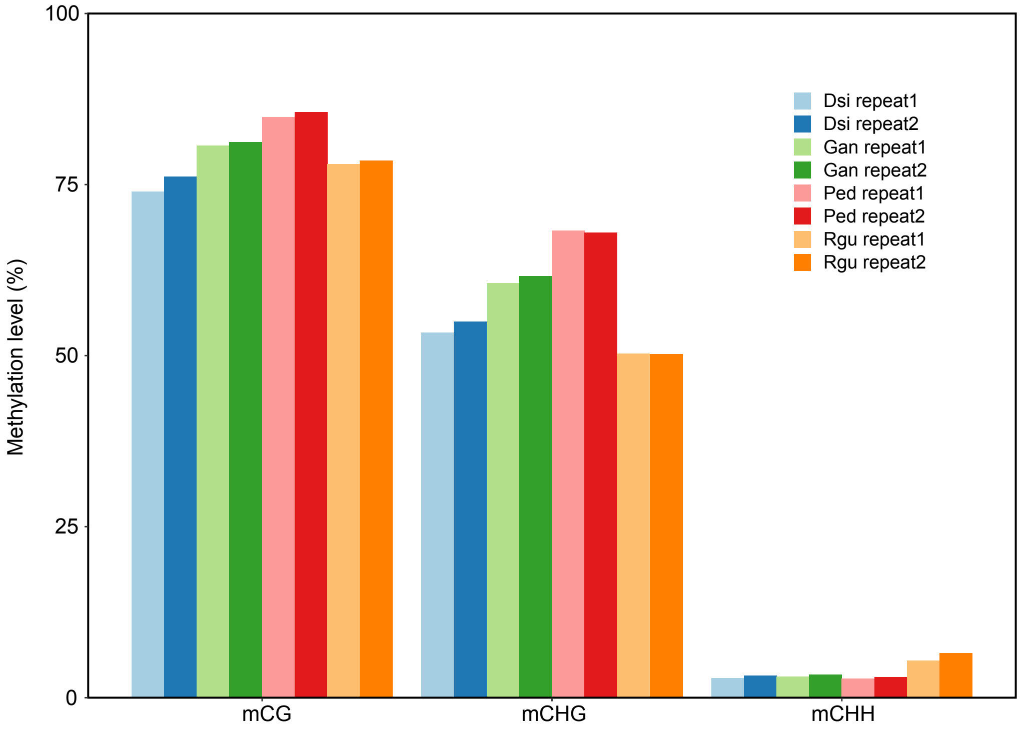

Supplementary Fig. 4. Comparison of global average DNA methylation level between herbaceous (*Ra. guianensis*, Rgu) and woody (*D. sinicus*, Dsi; *G. angustifolia*, Gan; *P. edulis*, Ped) bamboos. The whole genome bisulfite sequencing was conducted with two biological repeats.

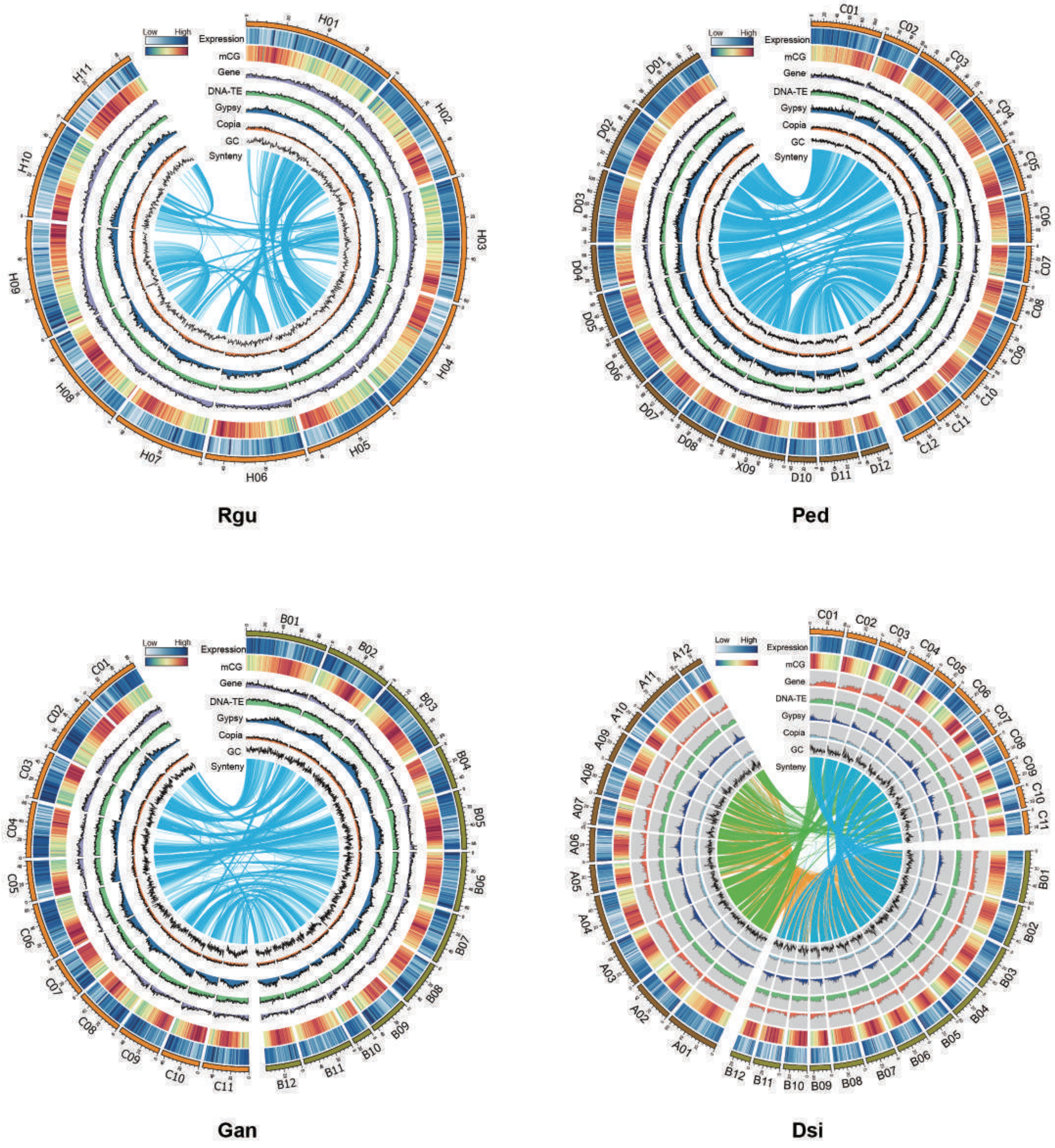

Supplementary Fig. 5. Circos diagrams showing distribution of genomic features for *Ra. guianensis* (Rgu), *G. angustifolia* (Gan), *P. edulis* (Ped) and *D. sinicus* (Dsi). Gene expression levels in leaf, mCG methylation levels, gene density, DNA transposon density, *Gypsy* retrotransposon density, *Copia* retrotransposon density, GC content and intra-genome gene synteny are shown from outer to inner tracts.

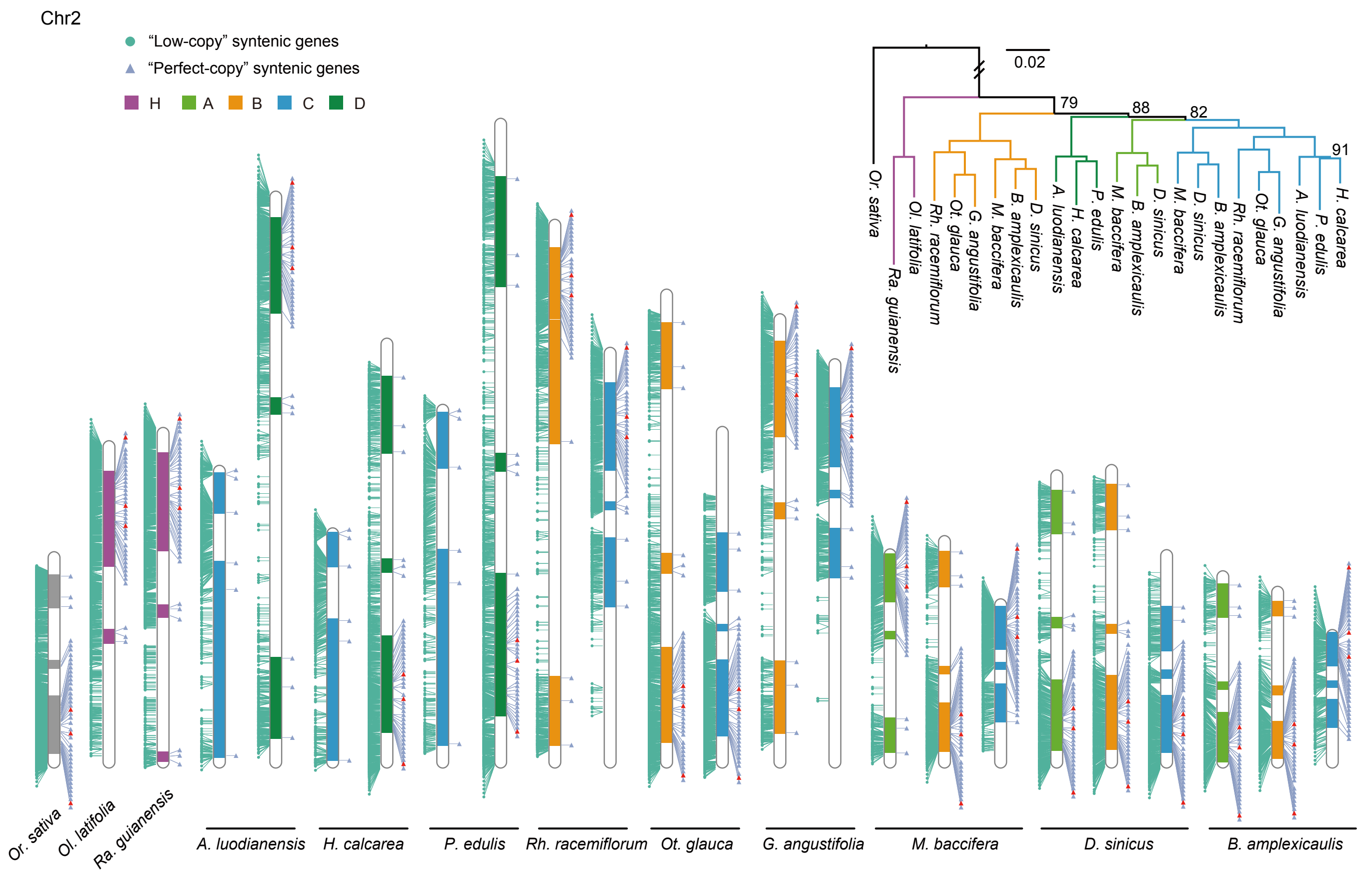
Chr2
"Low-copy" syntenic genes
"Perfect-copy" syntenic genes
H A B C D
0.02
79
88
82
91
Or. sativa
Ra. guianensis
Ol. latifolia
Rh. racemiflorum
Ot. glauca
G. angustifolia
M. baccifera
B. amplexicaulis
D. sinicus
A. luodianensis
H. calcarea
P. edulis
M. baccifera
B. amplexicaulis
D. sinicus
M. baccifera
D. sinicus
B. amplexicaulis
Rh. racemiflorum
Ot. glauca
G. angustifolia
A. luodianensis
P. edulis
H. calcarea
Or. sativa
Ol. latifolia
Ra. guianensis
A. luodianensis
H. calcarea
P. edulis
Rh. racemiflorum
Ot. glauca
G. angustifolia
M. baccifera
D. sinicus
B. amplexicaulis

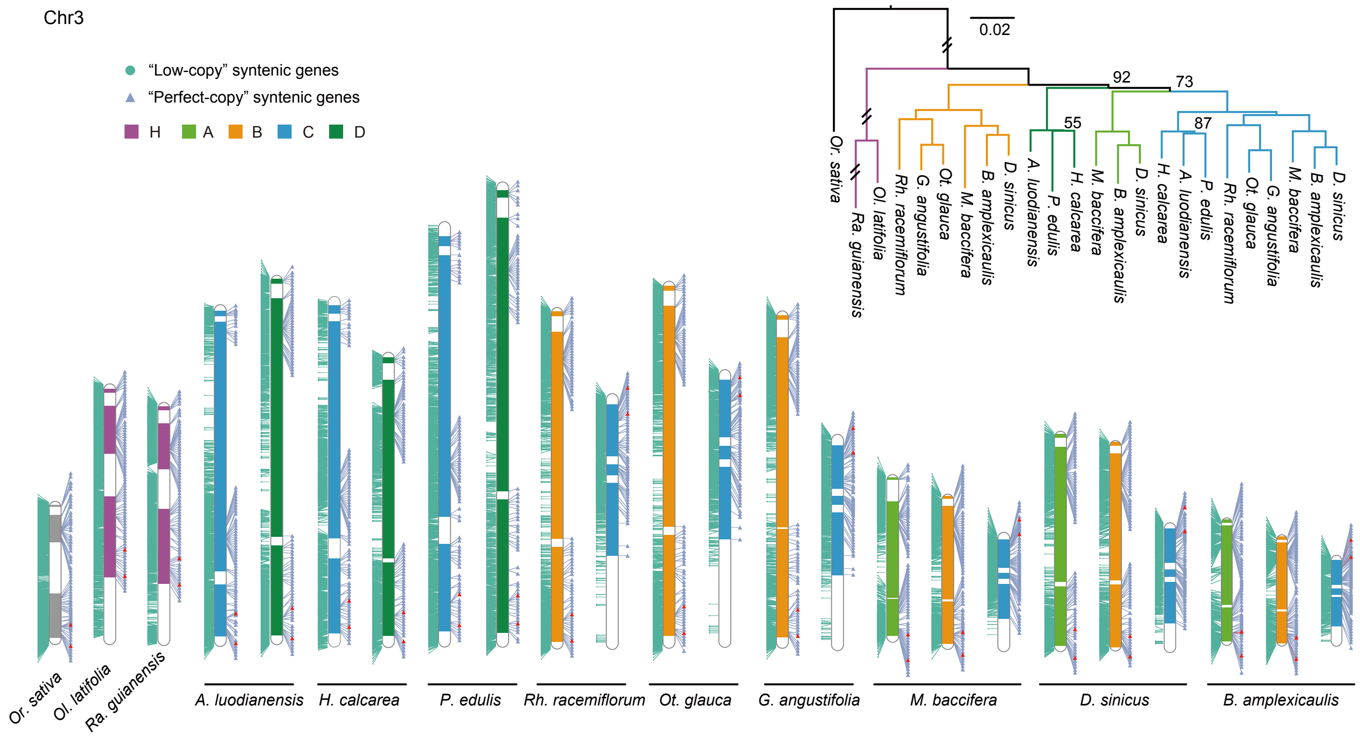
Chr3
"Low-copy" syntenic genes
"Perfect-copy" syntenic genes
H A B C D
0.02
92
73
55
87
Or. sativa
Ra. guianensis
Ol. latifolia
Rh. racemiflorum
G. angustifolia
Ot. glauca
M. baccifera
B. amplexicaulis
D. sinicus
A. luodianensis
P. edulis
H. calcarea
M. baccifera
B. amplexicaulis
D. sinicus
H. calcarea
A. luodianensis
P. edulis
Rh. racemiflorum
Ot. glauca
G. angustifolia
M. baccifera
B. amplexicaulis
D. sinicus
Or. sativa
Ol. latifolia
Ra. guianensis
A. luodianensis
H. calcarea
P. edulis
Rh. racemiflorum
Ot. glauca
G. angustifolia
M. baccifera
D. sinicus
B. amplexicaulis

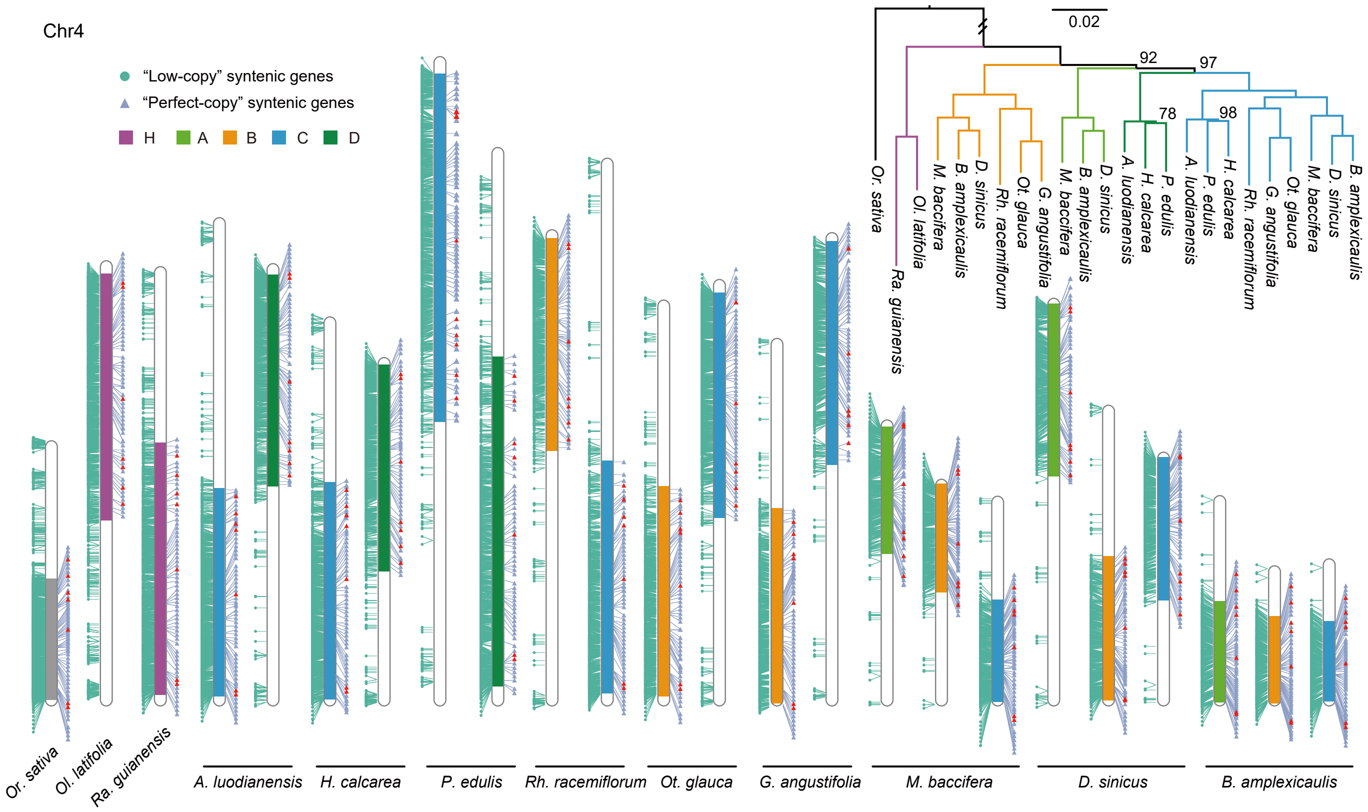

Chr4
"Low-copy" syntenic genes
"Perfect-copy" syntenic genes
H A B C D
0.02
92
97
78
98
Or. sativa
Ol. latifolia
Ra. guianensis
A. luodianensis
H. calcarea
P. edulis
Rh. racemiflorum
Ot. glauca
G. angustifolia
M. baccifera
D. sinicus
B. amplexicaulis

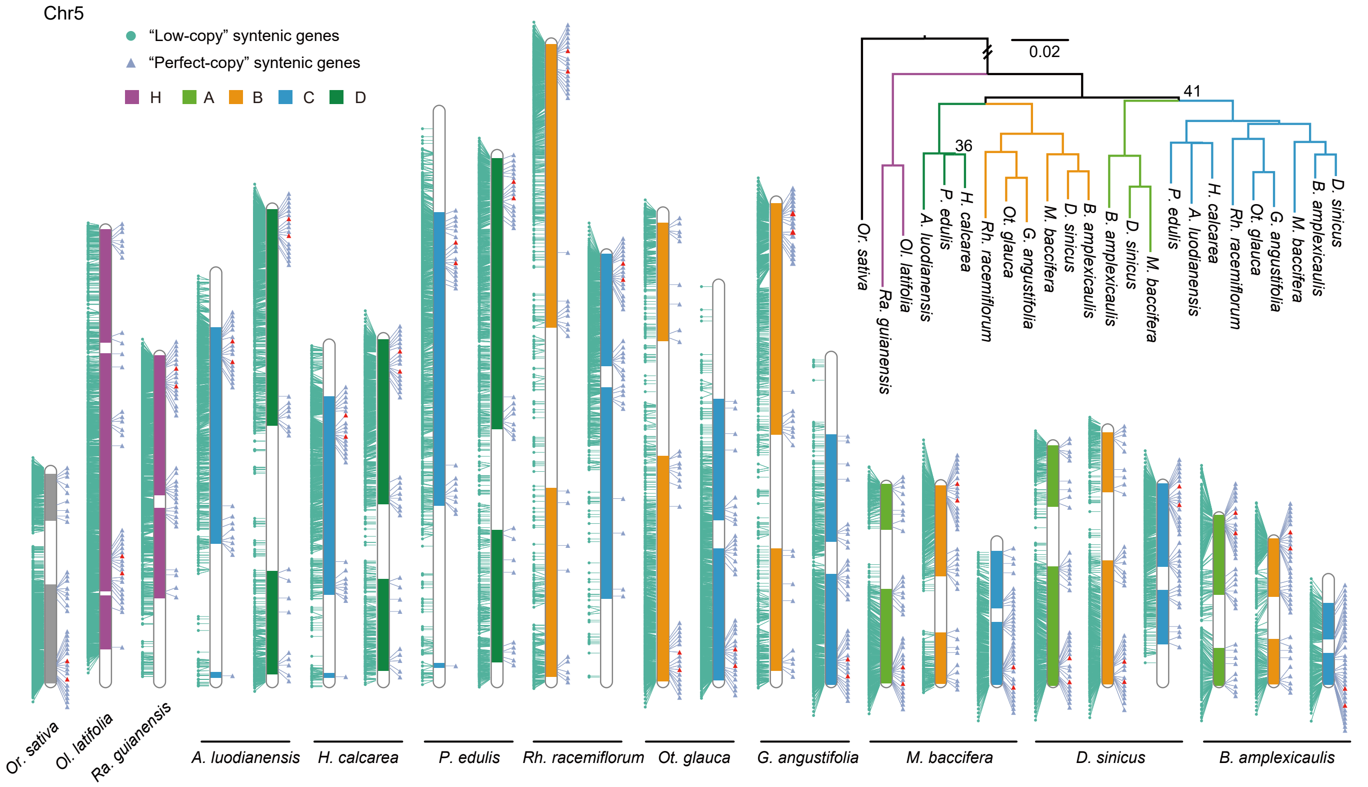

Chr5
"Low-copy" syntenic genes
"Perfect-copy" syntenic genes
H A B C D
0.02
41
36
Or. sativa
Ol. latifolia
Ra. guianensis
A. luodianensis
H. calcarea
P. edulis
Rh. racemiflorum
Ot. glauca
G. angustifolia
M. baccifera
D. sinicus
B. amplexicaulis

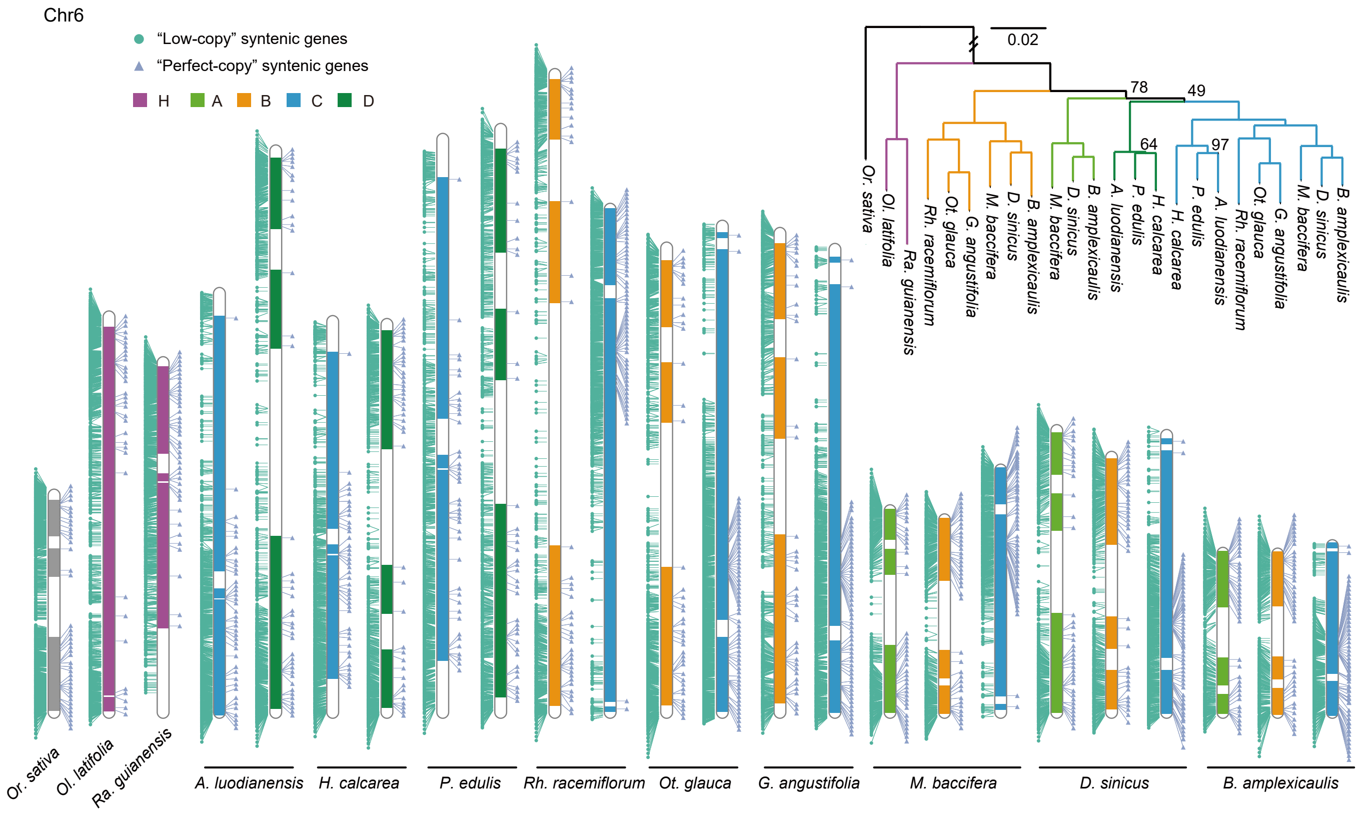
Chr6
"Low-copy" syntenic genes
"Perfect-copy" syntenic genes
H A B C D
0.02
78
49
64
97
Or. sativa
Ol. latifolia
Ra. guianensis
Rh. racemiflorum
Ot. glauca
G. angustifolia
M. baccifera
D. sinicus
B. amplexicaulis
M. baccifera
D. sinicus
B. amplexicaulis
A. luodianensis
P. edulis
H. calcarea
H. calcarea
P. edulis
A. luodianensis
Rh. racemiflorum
Ot. glauca
G. angustifolia
M. baccifera
D. sinicus
B. amplexicaulis
Or. sativa
Ol. latifolia
Ra. guianensis
A. luodianensis
H. calcarea
P. edulis
Rh. racemiflorum
Ot. glauca
G. angustifolia
M. baccifera
D. sinicus
B. amplexicaulis

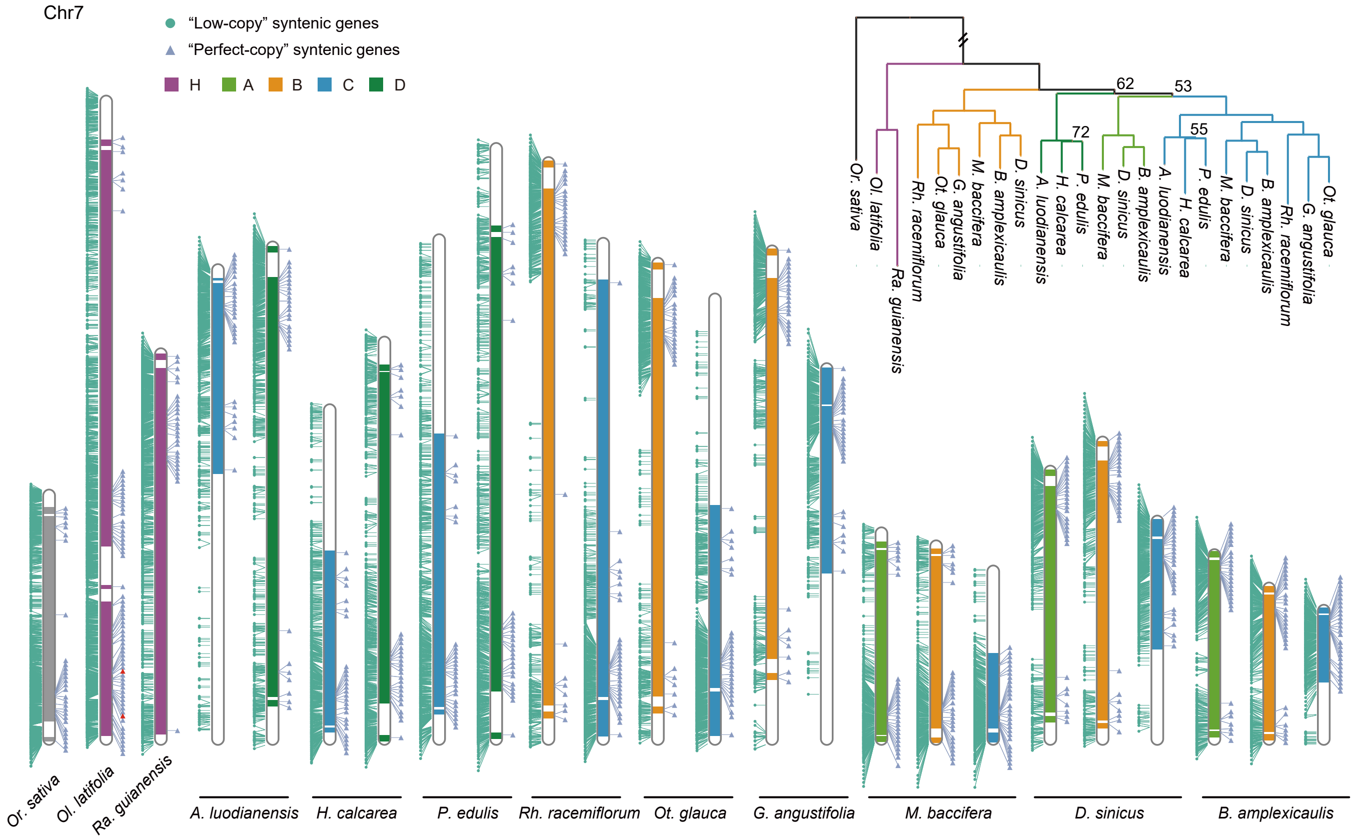
Chr7
"Low-copy" syntenic genes
"Perfect-copy" syntenic genes
H A B C D
62
53
72
55
Or. sativa
Ol. latifolia
Ra. guianensis
Rh. racemiflorum
Ot. glauca
G. angustifolia
M. baccifera
B. amplexicaulis
D. sinicus
A. luodianensis
H. calcarea
P. edulis
M. baccifera
D. sinicus
B. amplexicaulis
A. luodianensis
H. calcarea
P. edulis
M. baccifera
D. sinicus
B. amplexicaulis
Rh. racemiflorum
G. angustifolia
Ot. glauca
Or. sativa
Ol. latifolia
Ra. guianensis
A. luodianensis
H. calcarea
P. edulis
Rh. racemiflorum
Ot. glauca
G. angustifolia
M. baccifera
D. sinicus
B. amplexicaulis

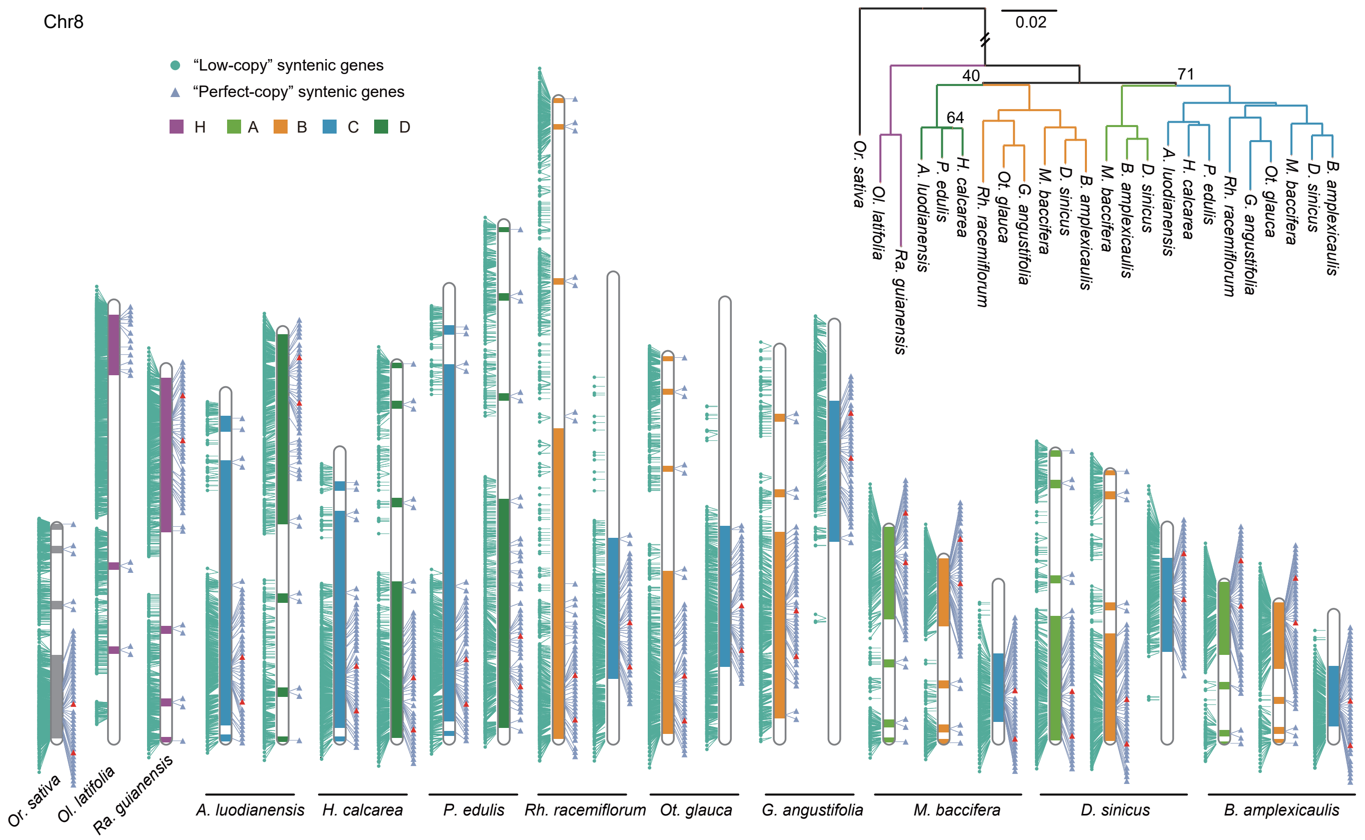
Chr8
"Low-copy" syntenic genes
"Perfect-copy" syntenic genes
H
A
B
C
D
0.02
40
71
64
Or. sativa
Ol. latifolia
Ra. guianensis
A. luodianensis
P. edulis
H. calcarea
Rh. racemiflorum
Ot. glauca
G. angustifolia
M. baccifera
D. sinicus
B. amplexicaulis
M. baccifera
B. amplexicaulis
D. sinicus
A. luodianensis
H. calcarea
P. edulis
Rh. racemiflorum
G. angustifolia
Ot. glauca
M. baccifera
D. sinicus
B. amplexicaulis
Or. sativa
Ol. latifolia
Ra. guianensis
A. luodianensis
H. calcarea
P. edulis
Rh. racemiflorum
Ot. glauca
G. angustifolia
M. baccifera
D. sinicus
B. amplexicaulis

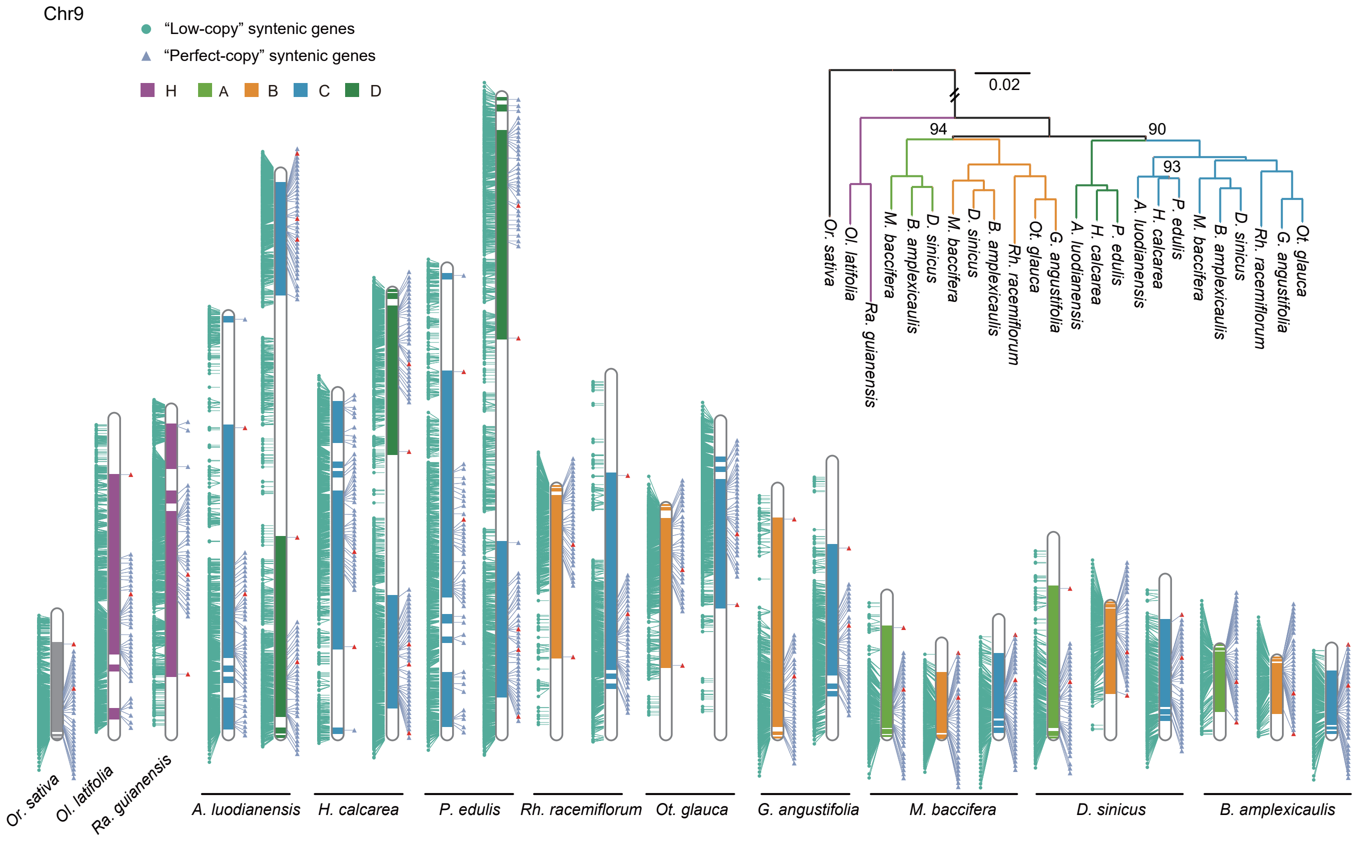
Chr9
"Low-copy" syntenic genes
"Perfect-copy" syntenic genes
H
A
B
C
D
0.02
94
90
93
Or. sativa
Ol. latifolia
Ra. guianensis
M. baccifera
B. amplexicaulis
D. sinicus
M. baccifera
D. sinicus
B. amplexicaulis
Rh. racemiflorum
Ot. glauca
G. angustifolia
A. luodianensis
H. calcarea
P. edulis
A. luodianensis
H. calcarea
P. edulis
M. baccifera
B. amplexicaulis
D. sinicus
Rh. racemiflorum
G. angustifolia
Ot. glauca
Or. sativa
Ol. latifolia
Ra. guianensis
A. luodianensis
H. calcarea
P. edulis
Rh. racemiflorum
Ot. glauca
G. angustifolia
M. baccifera
D. sinicus
B. amplexicaulis

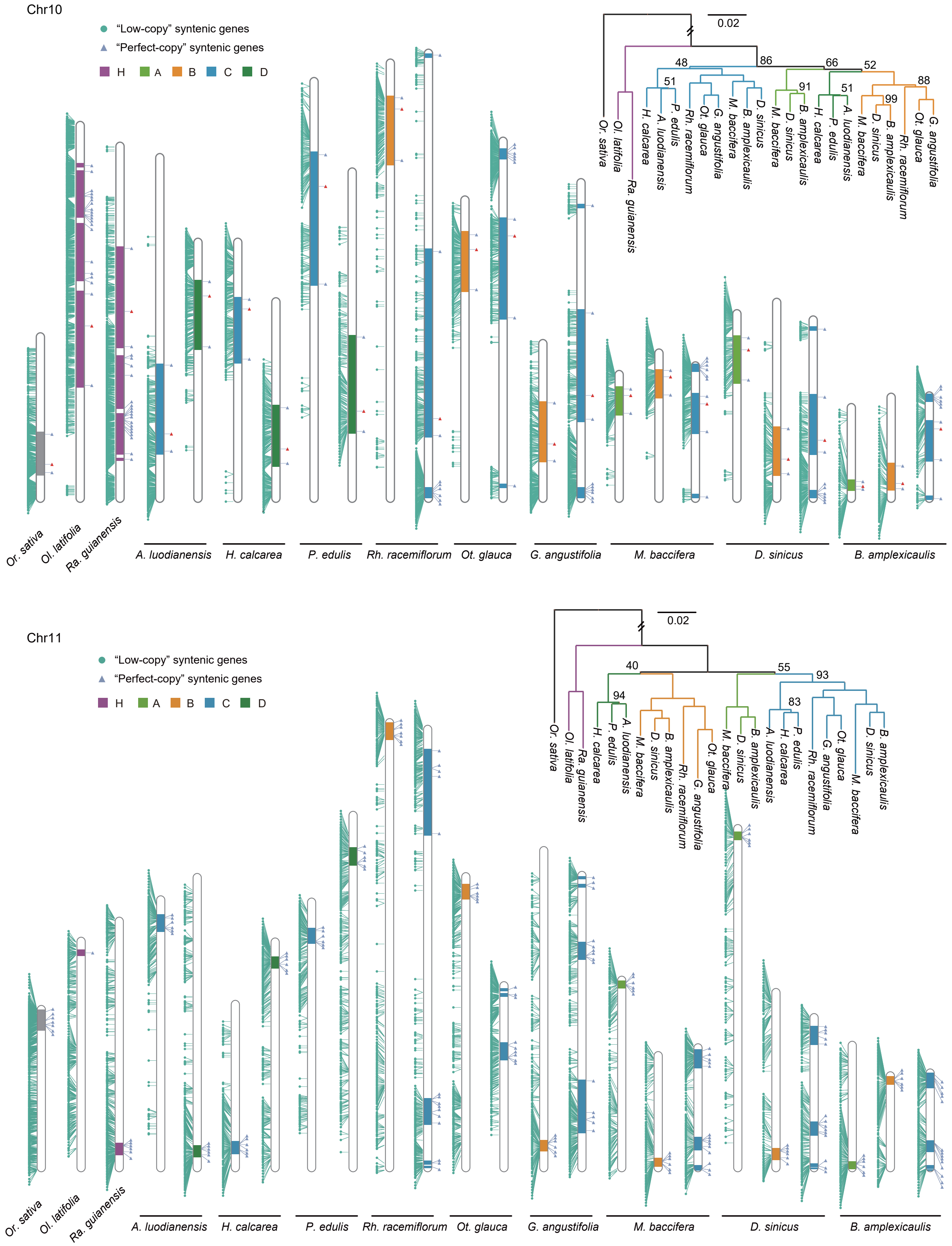

Chr10
"Low-copy" syntenic genes
"Perfect-copy" syntenic genes
H
A
B
C
D
0.02
48
86
66
52
51
91
51
88
99
Or. sativa
Ol. latifolia
Ra. guianensis
H. calcarea
A. luodianensis
P. edulis
Rh. racemiflorum
Ot. glauca
G. angustifolia
M. baccifera
B. amplexicaulis
D. sinicus
M. baccifera
D. sinicus
B. amplexicaulis
H. calcarea
P. edulis
A. luodianensis
M. baccifera
D. sinicus
B. amplexicaulis
Rh. racemiflorum
Ot. glauca
G. angustifolia
Or. sativa
Ol. latifolia
Ra. guianensis
A. luodianensis
H. calcarea
P. edulis
Rh. racemiflorum
Ot. glauca
G. angustifolia
M. baccifera
D. sinicus
B. amplexicaulis
Chr11
"Low-copy" syntenic genes
"Perfect-copy" syntenic genes
H
A
B
C
D
0.02
40
55
93
94
83
Or. sativa
Ol. latifolia
Ra. guianensis
H. calcarea
P. edulis
A. luodianensis
M. baccifera
D. sinicus
B. amplexicaulis
Rh. racemiflorum
G. angustifolia
Ot. glauca
M. baccifera
D. sinicus
B. amplexicaulis
A. luodianensis
H. calcarea
P. edulis
Rh. racemiflorum
G. angustifolia
Ot. glauca
M. baccifera
D. sinicus
B. amplexicaulis
Or. sativa
Ol. latifolia
Ra. guianensis
A. luodianensis
H. calcarea
P. edulis
Rh. racemiflorum
Ot. glauca
G. angustifolia
M. baccifera
D. sinicus
B. amplexicaulis

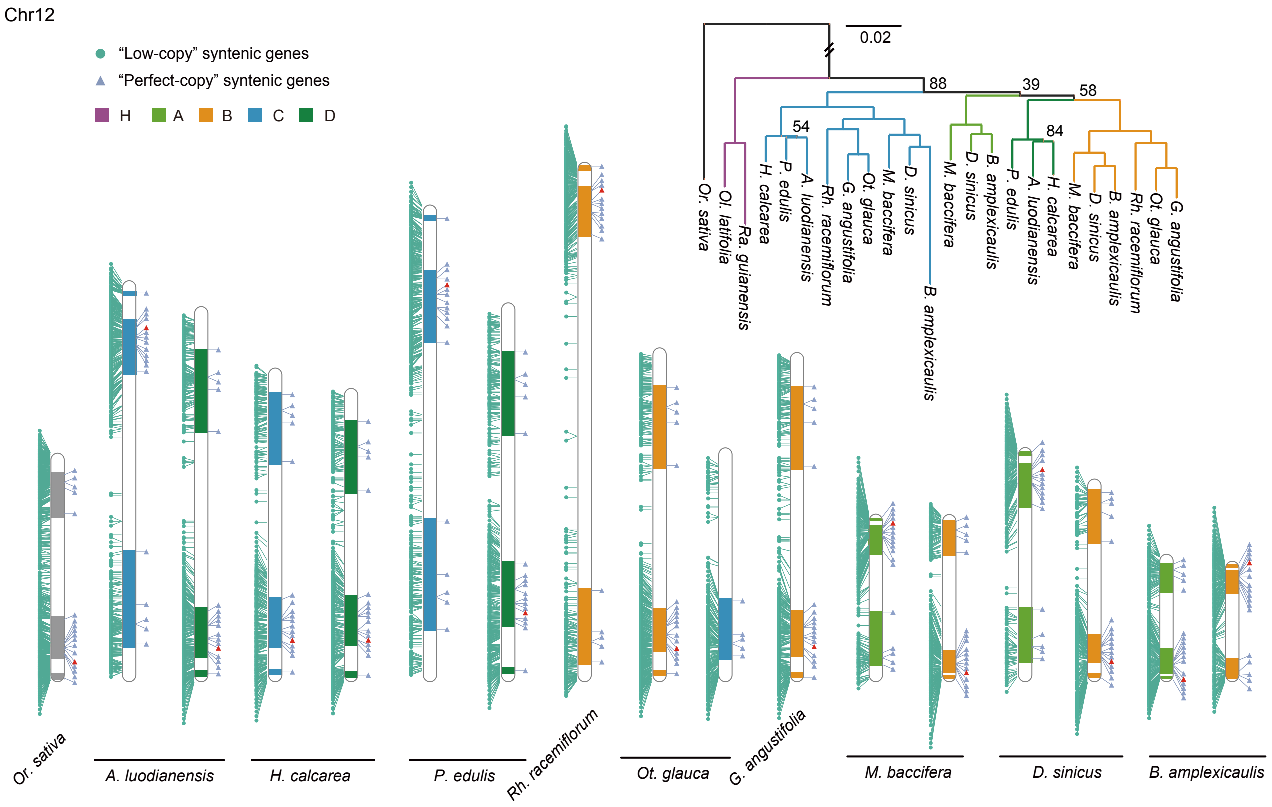

Supplementary Fig. 6. Distribution of "perfect-copy" (456) and "low-copy" (13,891) syntenic gene orthogroups along the chromosomes of 11 sampled bamboo genomes corresponding to the 12 rice chromosomes. Chrs2-12 are presented here, and see Extended Data Fig. 2a for the chr1. Each bar indicates a chromosome with green circles and blue triangles representing the locations of "low-copy" and "perfect-copy" syntenic genes, respectively. The red triangles represent those genes filtered by putative gene conversion or highly deviating from the ASTRAL species tree (26 in total). Colored bands represent blocks in which "perfect-copy" syntenic genes are clustered and different colors correspond to the identified subgenomes. The phylogenetic tree inferred by concatenated "perfect-copy" syntenic genes from the longest syntenic block for each chromosome was shown on the upper right. Bootstrap values are only shown for the nodes not supported by 100%.

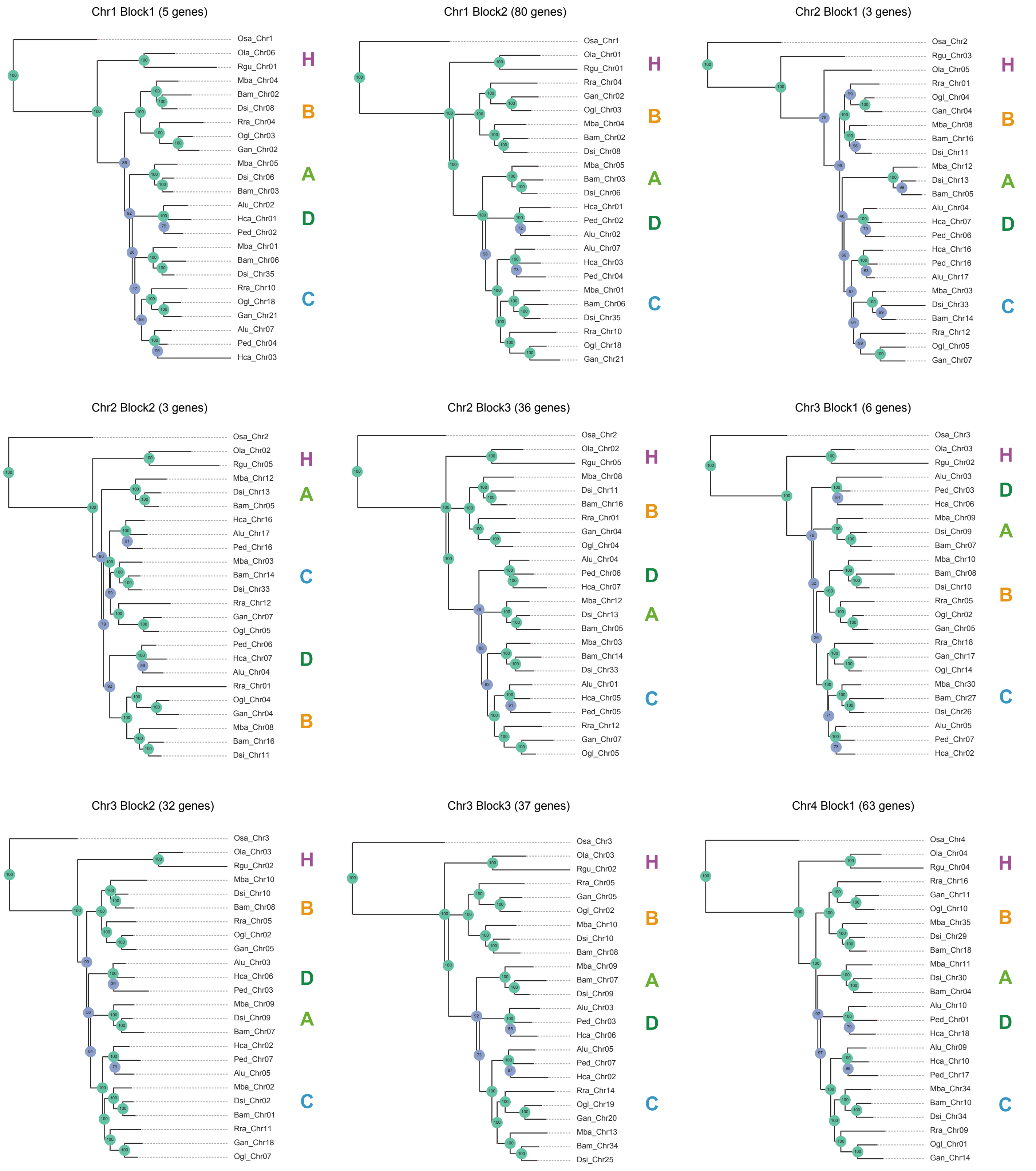

Chr1 Block1 (5 genes)
Osa_Chr1
Ola_Chr06
Rgu_Chr01
Mba_Chr04
Bam_Chr02
Dsi_Chr08
Rra_Chr04
Ogl_Chr03
Gan_Chr02
Mba_Chr05
Dsi_Chr06
Bam_Chr03
Alu_Chr02
Hca_Chr01
Ped_Chr02
Mba_Chr01
Bam_Chr06
Dsi_Chr35
Rra_Chr10
Ogl_Chr18
Gan_Chr21
Alu_Chr07
Ped_Chr04
Hca_Chr03
H
B
A
D
C
Chr1 Block2 (80 genes)
Osa_Chr1
Ola_Chr01
Rgu_Chr01
Rra_Chr04
Gan_Chr02
Ogl_Chr03
Mba_Chr04
Bam_Chr02
Dsi_Chr08
Mba_Chr05
Bam_Chr03
Dsi_Chr06
Hca_Chr01
Ped_Chr02
Alu_Chr02
Alu_Chr07
Hca_Chr03
Ped_Chr04
Mba_Chr01
Bam_Chr06
Dsi_Chr35
Rra_Chr10
Ogl_Chr18
Gan_Chr21
H
B
A
D
C
Chr2 Block1 (3 genes)
Osa_Chr2
Rgu_Chr03
Ola_Chr05
Rra_Chr01
Ogl_Chr04
Gan_Chr04
Mba_Chr08
Bam_Chr16
Dsi_Chr11
Mba_Chr12
Dsi_Chr13
Bam_Chr05
Alu_Chr04
Hca_Chr07
Ped_Chr06
Hca_Chr16
Ped_Chr16
Alu_Chr17
Mba_Chr03
Dsi_Chr33
Bam_Chr14
Rra_Chr12
Ogl_Chr05
Gan_Chr07
H
B
A
D
C
Chr2 Block2 (3 genes)
Osa_Chr2
Ola_Chr02
Rgu_Chr05
Mba_Chr12
Dsi_Chr13
Bam_Chr05
Hca_Chr16
Alu_Chr17
Ped_Chr16
Mba_Chr03
Bam_Chr14
Dsi_Chr33
Rra_Chr12
Gan_Chr07
Ogl_Chr05
Ped_Chr06
Hca_Chr07
Alu_Chr04
Rra_Chr01
Ogl_Chr04
Gan_Chr04
Mba_Chr08
Bam_Chr16
Dsi_Chr11
H
A
C
D
B
Chr2 Block3 (36 genes)
Osa_Chr2
Ola_Chr02
Rgu_Chr05
Mba_Chr08
Dsi_Chr11
Bam_Chr16
Rra_Chr01
Gan_Chr04
Ogl_Chr04
Alu_Chr04
Ped_Chr06
Hca_Chr07
Mba_Chr12
Dsi_Chr13
Bam_Chr05
Mba_Chr03
Bam_Chr14
Dsi_Chr33
Alu_Chr01
Hca_Chr05
Ped_Chr05
Rra_Chr12
Gan_Chr07
Ogl_Chr05
H
B
D
A
C
Chr3 Block1 (6 genes)
Osa_Chr3
Ola_Chr03
Rgu_Chr02
Alu_Chr03
Ped_Chr03
Hca_Chr06
Mba_Chr09
Dsi_Chr09
Bam_Chr07
Mba_Chr10
Bam_Chr08
Dsi_Chr10
Rra_Chr05
Ogl_Chr02
Gan_Chr05
Rra_Chr18
Gan_Chr17
Ogl_Chr14
Mba_Chr30
Bam_Chr27
Dsi_Chr26
Alu_Chr05
Ped_Chr07
Hca_Chr02
H
D
A
B
C
Chr3 Block2 (32 genes)
Osa_Chr3
Ola_Chr03
Rgu_Chr02
Mba_Chr10
Dsi_Chr10
Bam_Chr08
Rra_Chr05
Ogl_Chr02
Gan_Chr05
Alu_Chr03
Hca_Chr06
Ped_Chr03
Mba_Chr09
Dsi_Chr09
Bam_Chr07
Hca_Chr02
Ped_Chr07
Alu_Chr05
Mba_Chr02
Dsi_Chr02
Bam_Chr01
Rra_Chr11
Gan_Chr18
Ogl_Chr07
H
B
D
A
C
Chr3 Block3 (37 genes)
Osa_Chr3
Ola_Chr03
Rgu_Chr02
Rra_Chr05
Gan_Chr05
Ogl_Chr02
Mba_Chr10
Dsi_Chr10
Bam_Chr08
Mba_Chr09
Bam_Chr07
Dsi_Chr09
Alu_Chr03
Ped_Chr03
Hca_Chr06
Alu_Chr05
Ped_Chr07
Hca_Chr02
Rra_Chr14
Ogl_Chr19
Gan_Chr20
Mba_Chr13
Bam_Chr34
Dsi_Chr25
H
B
A
D
C
Chr4 Block1 (63 genes)
Osa_Chr4
Ola_Chr04
Rgu_Chr04
Rra_Chr16
Gan_Chr11
Ogl_Chr10
Mba_Chr35
Dsi_Chr29
Bam_Chr18
Mba_Chr11
Dsi_Chr30
Bam_Chr04
Alu_Chr10
Ped_Chr01
Hca_Chr18
Alu_Chr09
Hca_Chr10
Ped_Chr17
Mba_Chr34
Bam_Chr10
Dsi_Chr34
Rra_Chr09
Ogl_Chr01
Gan_Chr14
H
B
A
D
C

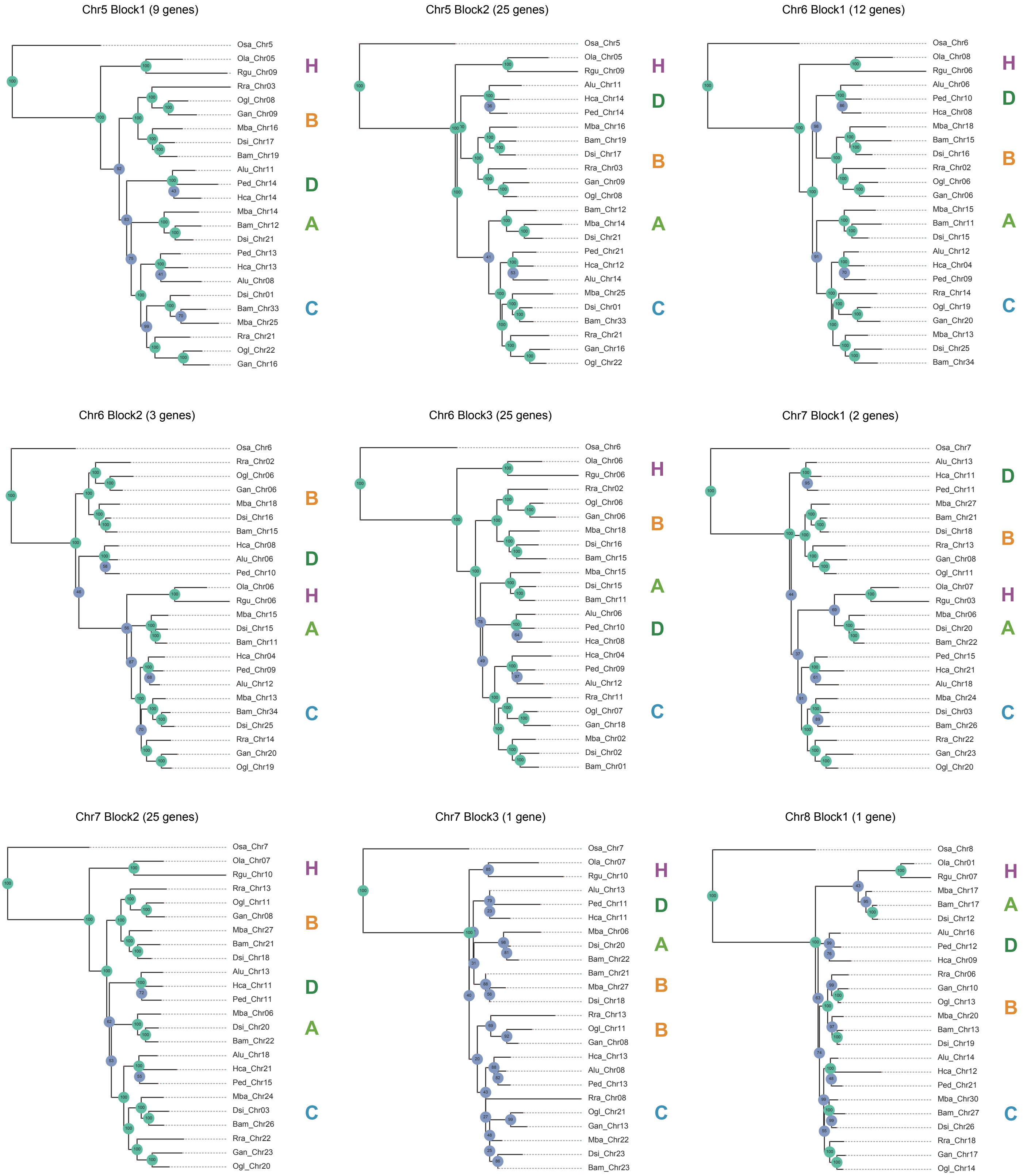
Chr5 Block1 (9 genes)
Osa_Chr5
Ola_Chr05
Rgu_Chr09
Rra_Chr03
Ogl_Chr08
Gan_Chr09
Mba_Chr16
Dsi_Chr17
Bam_Chr19
Alu_Chr11
Ped_Chr14
Hca_Chr14
Mba_Chr14
Bam_Chr12
Dsi_Chr21
Ped_Chr13
Hca_Chr13
Alu_Chr08
Dsi_Chr01
Bam_Chr33
Mba_Chr25
Rra_Chr21
Ogl_Chr22
Gan_Chr16
H
B
D
A
C
Chr5 Block2 (25 genes)
Osa_Chr5
Ola_Chr05
Rgu_Chr09
Alu_Chr11
Hca_Chr14
Ped_Chr14
Mba_Chr16
Bam_Chr19
Dsi_Chr17
Rra_Chr03
Gan_Chr09
Ogl_Chr08
Bam_Chr12
Mba_Chr14
Dsi_Chr21
Ped_Chr21
Hca_Chr12
Alu_Chr14
Mba_Chr25
Dsi_Chr01
Bam_Chr33
Rra_Chr21
Gan_Chr16
Ogl_Chr22
H
D
B
A
C
Chr6 Block1 (12 genes)
Osa_Chr6
Ola_Chr08
Rgu_Chr06
Alu_Chr06
Ped_Chr10
Hca_Chr08
Mba_Chr18
Bam_Chr15
Dsi_Chr16
Rra_Chr02
Ogl_Chr06
Gan_Chr06
Mba_Chr15
Bam_Chr11
Dsi_Chr15
Alu_Chr12
Hca_Chr04
Ped_Chr09
Rra_Chr14
Ogl_Chr19
Gan_Chr20
Mba_Chr13
Dsi_Chr25
Bam_Chr34
H
D
B
A
C
Chr6 Block2 (3 genes)
Osa_Chr6
Rra_Chr02
Ogl_Chr06
Gan_Chr06
Mba_Chr18
Dsi_Chr16
Bam_Chr15
Hca_Chr08
Alu_Chr06
Ped_Chr10
Ola_Chr06
Rgu_Chr06
Mba_Chr15
Dsi_Chr15
Bam_Chr11
Hca_Chr04
Ped_Chr09
Alu_Chr12
Mba_Chr13
Bam_Chr34
Dsi_Chr25
Rra_Chr14
Gan_Chr20
Ogl_Chr19
B
D
H
A
C
Chr6 Block3 (25 genes)
Osa_Chr6
Ola_Chr06
Rgu_Chr06
Rra_Chr02
Ogl_Chr06
Gan_Chr06
Mba_Chr18
Dsi_Chr16
Bam_Chr15
Mba_Chr15
Dsi_Chr15
Bam_Chr11
Alu_Chr06
Ped_Chr10
Hca_Chr08
Hca_Chr04
Ped_Chr09
Alu_Chr12
Rra_Chr11
Ogl_Chr07
Gan_Chr18
Mba_Chr02
Dsi_Chr02
Bam_Chr01
H
B
A
D
C
Chr7 Block1 (2 genes)
Osa_Chr7
Alu_Chr13
Hca_Chr11
Ped_Chr11
Mba_Chr27
Bam_Chr21
Dsi_Chr18
Rra_Chr13
Gan_Chr08
Ogl_Chr11
Ola_Chr07
Rgu_Chr03
Mba_Chr06
Dsi_Chr20
Bam_Chr22
Ped_Chr15
Hca_Chr21
Alu_Chr18
Mba_Chr24
Dsi_Chr03
Bam_Chr26
Rra_Chr22
Gan_Chr23
Ogl_Chr20
D
B
H
A
C
Chr7 Block2 (25 genes)
Osa_Chr7
Ola_Chr07
Rgu_Chr10
Rra_Chr13
Ogl_Chr11
Gan_Chr08
Mba_Chr27
Bam_Chr21
Dsi_Chr18
Alu_Chr13
Hca_Chr11
Ped_Chr11
Mba_Chr06
Dsi_Chr20
Bam_Chr22
Alu_Chr18
Hca_Chr21
Ped_Chr15
Mba_Chr24
Dsi_Chr03
Bam_Chr26
Rra_Chr22
Gan_Chr23
Ogl_Chr20
H
B
D
A
C
Chr7 Block3 (1 gene)
Osa_Chr7
Ola_Chr07
Rgu_Chr10
Alu_Chr13
Ped_Chr11
Hca_Chr11
Mba_Chr06
Dsi_Chr20
Bam_Chr22
Bam_Chr21
Mba_Chr27
Dsi_Chr18
Rra_Chr13
Ogl_Chr11
Gan_Chr08
Hca_Chr13
Alu_Chr08
Ped_Chr13
Rra_Chr08
Ogl_Chr21
Gan_Chr13
Mba_Chr22
Dsi_Chr23
Bam_Chr23
H
D
A
B
B
C
Chr8 Block1 (1 gene)
Osa_Chr8
Ola_Chr01
Rgu_Chr07
Mba_Chr17
Bam_Chr17
Dsi_Chr12
Alu_Chr16
Ped_Chr12
Hca_Chr09
Rra_Chr06
Gan_Chr10
Ogl_Chr13
Mba_Chr20
Bam_Chr13
Dsi_Chr19
Alu_Chr14
Hca_Chr12
Ped_Chr21
Mba_Chr30
Bam_Chr27
Dsi_Chr26
Rra_Chr18
Gan_Chr17
Ogl_Chr14
H
A
D
B
C

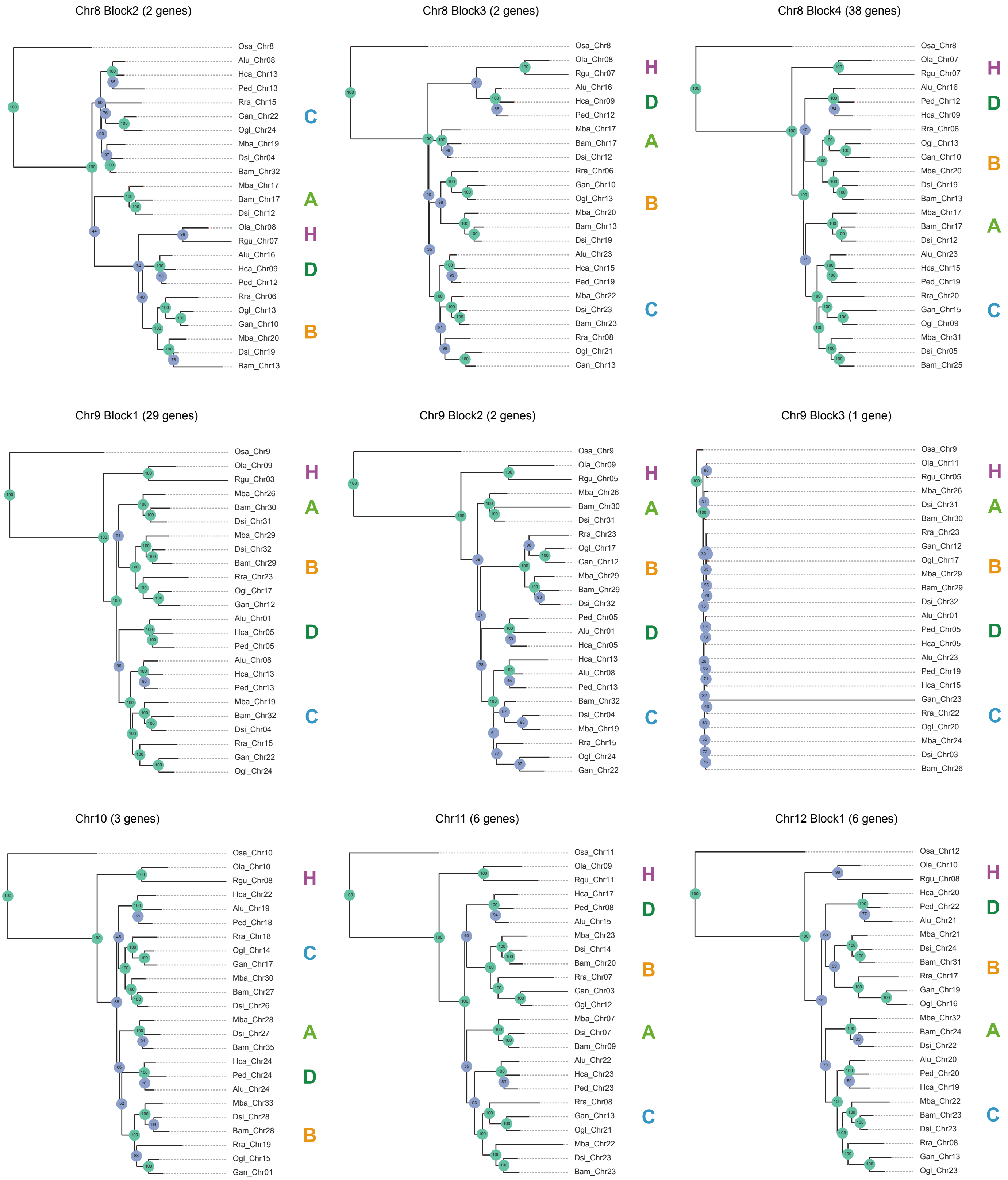
Chr8 Block2 (2 genes)
Osa_Chr8
Alu_Chr08
Hca_Chr13
Ped_Chr13
Rra_Chr15
Gan_Chr22
Ogl_Chr24
Mba_Chr19
Dsi_Chr04
Bam_Chr32
Mba_Chr17
Bam_Chr17
Dsi_Chr12
Ola_Chr08
Rgu_Chr07
Alu_Chr16
Hca_Chr09
Ped_Chr12
Rra_Chr06
Ogl_Chr13
Gan_Chr10
Mba_Chr20
Dsi_Chr19
Bam_Chr13
C
A
H
D
B
Chr8 Block3 (2 genes)
Osa_Chr8
Ola_Chr08
Rgu_Chr07
Alu_Chr16
Hca_Chr09
Ped_Chr12
Mba_Chr17
Bam_Chr17
Dsi_Chr12
Rra_Chr06
Gan_Chr10
Ogl_Chr13
Mba_Chr20
Bam_Chr13
Dsi_Chr19
Alu_Chr23
Hca_Chr15
Ped_Chr19
Mba_Chr22
Dsi_Chr23
Bam_Chr23
Rra_Chr08
Ogl_Chr21
Gan_Chr13
H
D
A
B
C
Chr8 Block4 (38 genes)
Osa_Chr8
Ola_Chr07
Rgu_Chr07
Alu_Chr16
Ped_Chr12
Hca_Chr09
Rra_Chr06
Ogl_Chr13
Gan_Chr10
Mba_Chr20
Dsi_Chr19
Bam_Chr13
Mba_Chr17
Bam_Chr17
Dsi_Chr12
Alu_Chr23
Hca_Chr15
Ped_Chr19
Rra_Chr20
Gan_Chr15
Ogl_Chr09
Mba_Chr31
Dsi_Chr05
Bam_Chr25
H
D
B
A
C
Chr9 Block1 (29 genes)
Osa_Chr9
Ola_Chr09
Rgu_Chr03
Mba_Chr26
Bam_Chr30
Dsi_Chr31
Mba_Chr29
Dsi_Chr32
Bam_Chr29
Rra_Chr23
Ogl_Chr17
Gan_Chr12
Alu_Chr01
Hca_Chr05
Ped_Chr05
Alu_Chr08
Hca_Chr13
Ped_Chr13
Mba_Chr19
Bam_Chr32
Dsi_Chr04
Rra_Chr15
Gan_Chr22
Ogl_Chr24
H
A
B
D
C
Chr9 Block2 (2 genes)
Osa_Chr9
Ola_Chr09
Rgu_Chr05
Mba_Chr26
Bam_Chr30
Dsi_Chr31
Rra_Chr23
Ogl_Chr17
Gan_Chr12
Mba_Chr29
Bam_Chr29
Dsi_Chr32
Ped_Chr05
Alu_Chr01
Hca_Chr05
Hca_Chr13
Alu_Chr08
Ped_Chr13
Bam_Chr32
Dsi_Chr04
Mba_Chr19
Rra_Chr15
Ogl_Chr24
Gan_Chr22
H
A
B
D
C
Chr9 Block3 (1 gene)
Osa_Chr9
Ola_Chr11
Rgu_Chr05
Mba_Chr26
Dsi_Chr31
Bam_Chr30
Rra_Chr23
Gan_Chr12
Ogl_Chr17
Mba_Chr29
Bam_Chr29
Dsi_Chr32
Alu_Chr01
Ped_Chr05
Hca_Chr05
Alu_Chr23
Ped_Chr19
Hca_Chr15
Gan_Chr23
Rra_Chr22
Ogl_Chr20
Mba_Chr24
Dsi_Chr03
Bam_Chr26
H
A
B
D
C
Chr10 (3 genes)
Osa_Chr10
Ola_Chr10
Rgu_Chr08
Hca_Chr22
Alu_Chr19
Ped_Chr18
Rra_Chr18
Ogl_Chr14
Gan_Chr17
Mba_Chr30
Bam_Chr27
Dsi_Chr26
Mba_Chr28
Dsi_Chr27
Bam_Chr35
Hca_Chr24
Ped_Chr24
Alu_Chr24
Mba_Chr33
Dsi_Chr28
Bam_Chr28
Rra_Chr19
Ogl_Chr15
Gan_Chr01
H
C
A
D
B
Chr11 (6 genes)
Osa_Chr11
Ola_Chr09
Rgu_Chr11
Hca_Chr17
Ped_Chr08
Alu_Chr15
Mba_Chr23
Dsi_Chr14
Bam_Chr20
Rra_Chr07
Gan_Chr03
Ogl_Chr12
Mba_Chr07
Dsi_Chr07
Bam_Chr09
Alu_Chr22
Hca_Chr23
Ped_Chr23
Rra_Chr08
Gan_Chr13
Ogl_Chr21
Mba_Chr22
Dsi_Chr23
Bam_Chr23
H
D
B
A
C
Chr12 Block1 (6 genes)
Osa_Chr12
Ola_Chr10
Rgu_Chr08
Hca_Chr20
Ped_Chr22
Alu_Chr21
Mba_Chr21
Dsi_Chr24
Bam_Chr31
Rra_Chr17
Gan_Chr19
Ogl_Chr16
Mba_Chr32
Bam_Chr24
Dsi_Chr22
Alu_Chr20
Ped_Chr20
Hca_Chr19
Mba_Chr22
Bam_Chr23
Dsi_Chr23
Rra_Chr08
Gan_Chr13
Ogl_Chr23
H
D
B
A
C

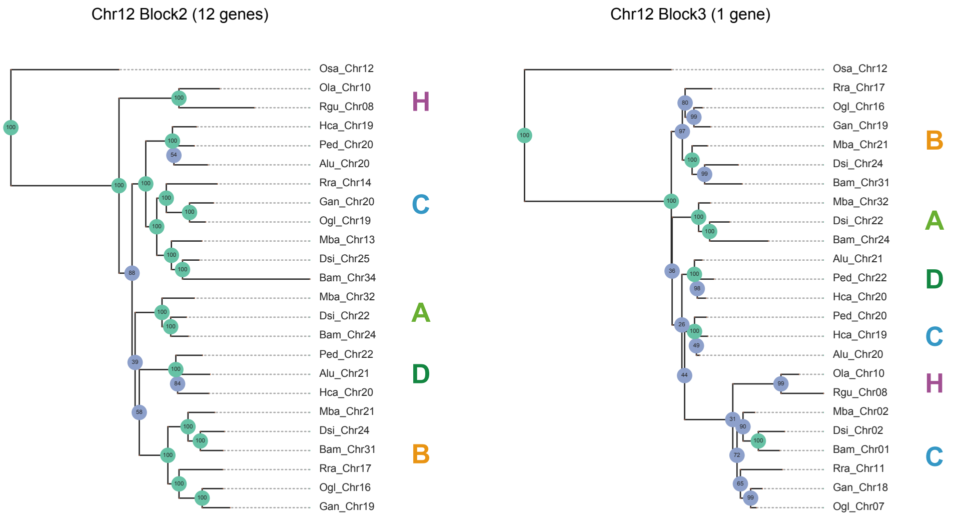

Supplementary Fig. 7. Identification of the subgenomes in woody bamboos based on phylogenetic topologies inferred by a total of 456 "perfect-copy" syntenic gene orthogroups from 29 syntenic blocks spanning the whole genomes. The phylogenetic tree of each syntenic block is shown, with bootstrap support values on nodes and those less than 100% in blue. The tip labels are represented by the abbreviation of the species name and the initial pseudo-chromosome number constructed by Hi-C connected by an underline. Each identified clade is marked by its comprising subgenome.

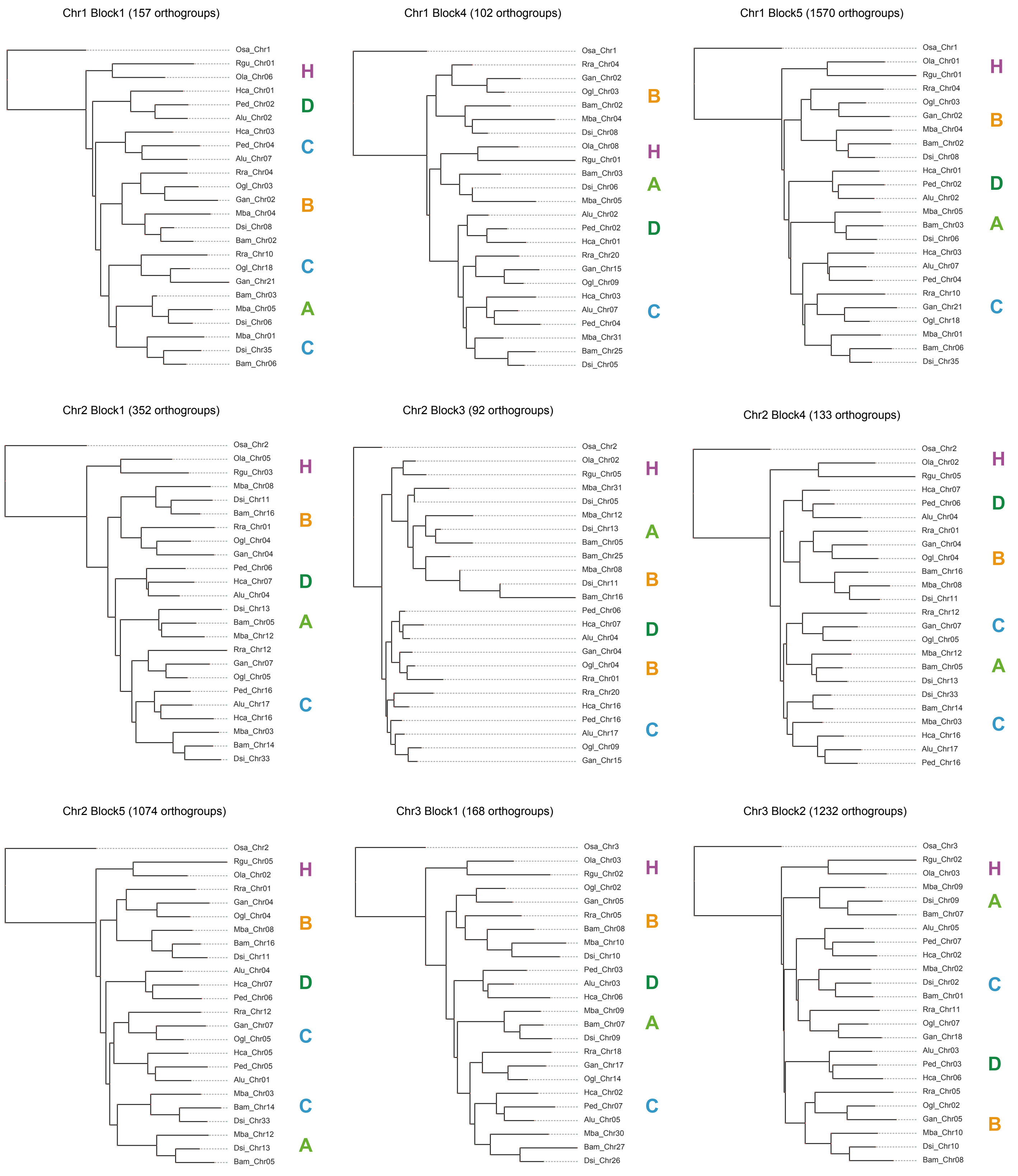

Chr1 Block1 (157 orthogroups)
Osa_Chr1
Rgu_Chr01
Ola_Chr06
Hca_Chr01
Ped_Chr02
Alu_Chr02
Hca_Chr03
Ped_Chr04
Alu_Chr07
Rra_Chr04
Ogl_Chr03
Gan_Chr02
Mba_Chr04
Dsi_Chr08
Bam_Chr02
Rra_Chr10
Ogl_Chr18
Gan_Chr21
Bam_Chr03
Mba_Chr05
Dsi_Chr06
Mba_Chr01
Dsi_Chr35
Bam_Chr06
H
D
C
B
C
A
C
Chr1 Block4 (102 orthogroups)
Osa_Chr1
Rra_Chr04
Gan_Chr02
Ogl_Chr03
Bam_Chr02
Mba_Chr04
Dsi_Chr08
Ola_Chr08
Rgu_Chr01
Bam_Chr03
Dsi_Chr06
Mba_Chr05
Alu_Chr02
Ped_Chr02
Hca_Chr01
Rra_Chr20
Gan_Chr15
Ogl_Chr09
Hca_Chr03
Alu_Chr07
Ped_Chr04
Mba_Chr31
Bam_Chr25
Dsi_Chr05
B
H
A
D
C
Chr1 Block5 (1570 orthogroups)
Osa_Chr1
Ola_Chr01
Rgu_Chr01
Rra_Chr04
Ogl_Chr03
Gan_Chr02
Mba_Chr04
Bam_Chr02
Dsi_Chr08
Hca_Chr01
Ped_Chr02
Alu_Chr02
Mba_Chr05
Bam_Chr03
Dsi_Chr06
Hca_Chr03
Alu_Chr07
Ped_Chr04
Rra_Chr10
Gan_Chr21
Ogl_Chr18
Mba_Chr01
Bam_Chr06
Dsi_Chr35
H
B
D
A
C
Chr2 Block1 (352 orthogroups)
Osa_Chr2
Ola_Chr05
Rgu_Chr03
Mba_Chr08
Dsi_Chr11
Bam_Chr16
Rra_Chr01
Ogl_Chr04
Gan_Chr04
Ped_Chr06
Hca_Chr07
Alu_Chr04
Dsi_Chr13
Bam_Chr05
Mba_Chr12
Rra_Chr12
Gan_Chr07
Ogl_Chr05
Ped_Chr16
Alu_Chr17
Hca_Chr16
Mba_Chr03
Bam_Chr14
Dsi_Chr33
H
B
D
A
C
Chr2 Block3 (92 orthogroups)
Osa_Chr2
Ola_Chr02
Rgu_Chr05
Mba_Chr31
Dsi_Chr05
Mba_Chr12
Dsi_Chr13
Bam_Chr05
Bam_Chr25
Mba_Chr08
Dsi_Chr11
Bam_Chr16
Ped_Chr06
Hca_Chr07
Alu_Chr04
Gan_Chr04
Ogl_Chr04
Rra_Chr01
Rra_Chr20
Hca_Chr16
Ped_Chr16
Alu_Chr17
Ogl_Chr09
Gan_Chr15
H
A
B
D
B
C
Chr2 Block4 (133 orthogroups)
Osa_Chr2
Ola_Chr02
Rgu_Chr05
Hca_Chr07
Ped_Chr06
Alu_Chr04
Rra_Chr01
Gan_Chr04
Ogl_Chr04
Bam_Chr16
Mba_Chr08
Dsi_Chr11
Rra_Chr12
Gan_Chr07
Ogl_Chr05
Mba_Chr12
Bam_Chr05
Dsi_Chr13
Dsi_Chr33
Bam_Chr14
Mba_Chr03
Hca_Chr16
Alu_Chr17
Ped_Chr16
H
D
B
C
A
C
Chr2 Block5 (1074 orthogroups)
Osa_Chr2
Rgu_Chr05
Ola_Chr02
Rra_Chr01
Gan_Chr04
Ogl_Chr04
Mba_Chr08
Bam_Chr16
Dsi_Chr11
Alu_Chr04
Hca_Chr07
Ped_Chr06
Rra_Chr12
Gan_Chr07
Ogl_Chr05
Hca_Chr05
Ped_Chr05
Alu_Chr01
Mba_Chr03
Bam_Chr14
Dsi_Chr33
Mba_Chr12
Dsi_Chr13
Bam_Chr05
H
B
D
C
C
A
Chr3 Block1 (168 orthogroups)
Osa_Chr3
Ola_Chr03
Rgu_Chr02
Ogl_Chr02
Gan_Chr05
Rra_Chr05
Bam_Chr08
Mba_Chr10
Dsi_Chr10
Ped_Chr03
Alu_Chr03
Hca_Chr06
Mba_Chr09
Bam_Chr07
Dsi_Chr09
Rra_Chr18
Gan_Chr17
Ogl_Chr14
Hca_Chr02
Ped_Chr07
Alu_Chr05
Mba_Chr30
Bam_Chr27
Dsi_Chr26
H
B
D
A
C
Chr3 Block2 (1232 orthogroups)
Osa_Chr3
Rgu_Chr02
Ola_Chr03
Mba_Chr09
Dsi_Chr09
Bam_Chr07
Alu_Chr05
Ped_Chr07
Hca_Chr02
Mba_Chr02
Dsi_Chr02
Bam_Chr01
Rra_Chr11
Ogl_Chr07
Gan_Chr18
Alu_Chr03
Ped_Chr03
Hca_Chr06
Rra_Chr05
Ogl_Chr02
Gan_Chr05
Mba_Chr10
Dsi_Chr10
Bam_Chr08
H
A
C
D
B

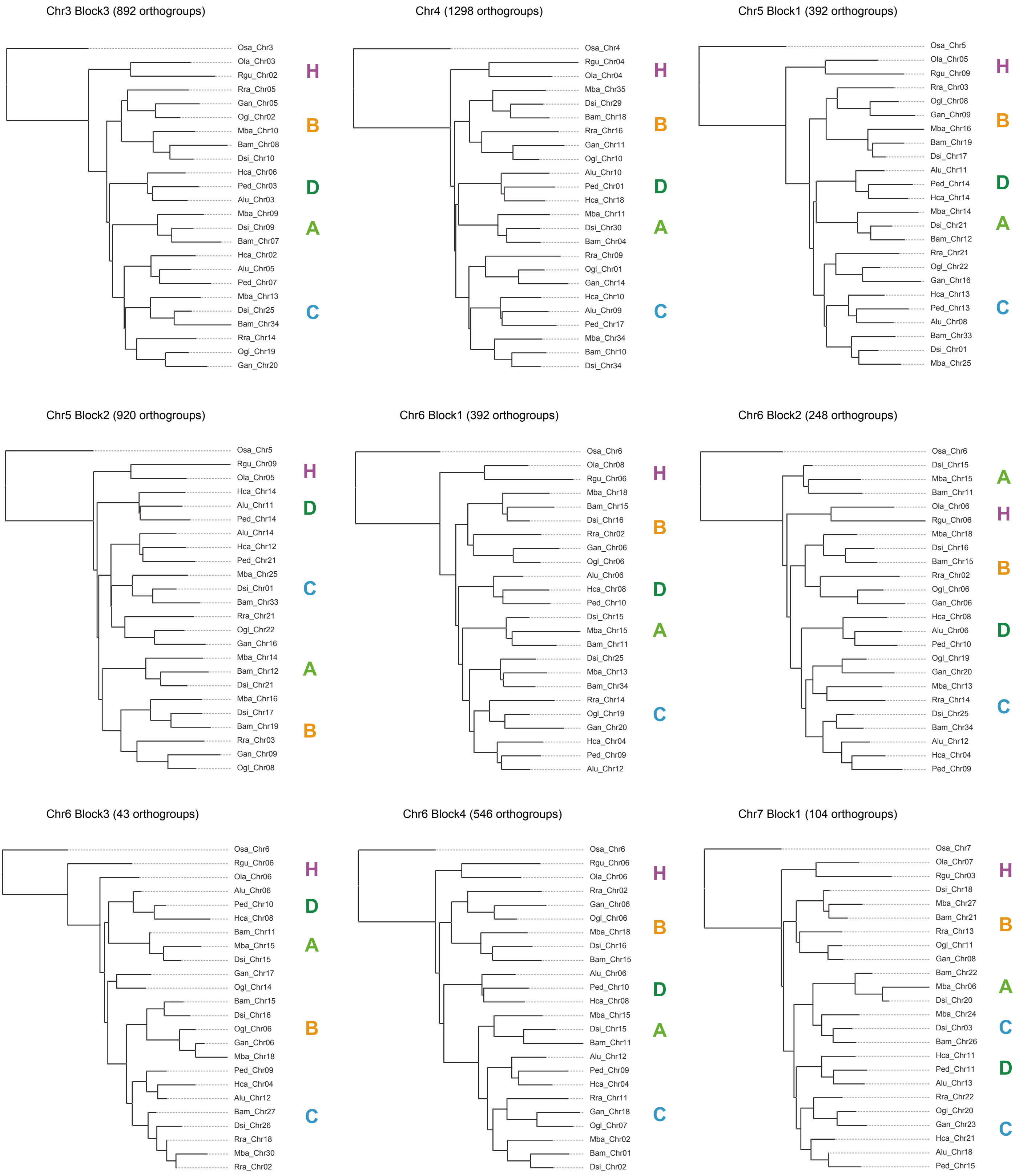
Chr3 Block3 (892 orthogroups)
Osa_Chr3
Ola_Chr03
Rgu_Chr02
Rra_Chr05
Gan_Chr05
Ogl_Chr02
Mba_Chr10
Bam_Chr08
Dsi_Chr10
Hca_Chr06
Ped_Chr03
Alu_Chr03
Mba_Chr09
Dsi_Chr09
Bam_Chr07
Hca_Chr02
Alu_Chr05
Ped_Chr07
Mba_Chr13
Dsi_Chr25
Bam_Chr34
Rra_Chr14
Ogl_Chr19
Gan_Chr20
H
B
D
A
C
Chr4 (1298 orthogroups)
Osa_Chr4
Rgu_Chr04
Ola_Chr04
Mba_Chr35
Dsi_Chr29
Bam_Chr18
Rra_Chr16
Gan_Chr11
Ogl_Chr10
Alu_Chr10
Ped_Chr01
Hca_Chr18
Mba_Chr11
Dsi_Chr30
Bam_Chr04
Rra_Chr09
Ogl_Chr01
Gan_Chr14
Hca_Chr10
Alu_Chr09
Ped_Chr17
Mba_Chr34
Bam_Chr10
Dsi_Chr34
H
B
D
A
C
Chr5 Block1 (392 orthogroups)
Osa_Chr5
Ola_Chr05
Rgu_Chr09
Rra_Chr03
Ogl_Chr08
Gan_Chr09
Mba_Chr16
Bam_Chr19
Dsi_Chr17
Alu_Chr11
Ped_Chr14
Hca_Chr14
Mba_Chr14
Dsi_Chr21
Bam_Chr12
Rra_Chr21
Ogl_Chr22
Gan_Chr16
Hca_Chr13
Ped_Chr13
Alu_Chr08
Bam_Chr33
Dsi_Chr01
Mba_Chr25
H
B
D
A
C
Chr5 Block2 (920 orthogroups)
Osa_Chr5
Rgu_Chr09
Ola_Chr05
Hca_Chr14
Alu_Chr11
Ped_Chr14
Alu_Chr14
Hca_Chr12
Ped_Chr21
Mba_Chr25
Dsi_Chr01
Bam_Chr33
Rra_Chr21
Ogl_Chr22
Gan_Chr16
Mba_Chr14
Bam_Chr12
Dsi_Chr21
Mba_Chr16
Dsi_Chr17
Bam_Chr19
Rra_Chr03
Gan_Chr09
Ogl_Chr08
H
D
C
A
B
Chr6 Block1 (392 orthogroups)
Osa_Chr6
Ola_Chr08
Rgu_Chr06
Mba_Chr18
Bam_Chr15
Dsi_Chr16
Rra_Chr02
Gan_Chr06
Ogl_Chr06
Alu_Chr06
Hca_Chr08
Ped_Chr10
Dsi_Chr15
Mba_Chr15
Bam_Chr11
Dsi_Chr25
Mba_Chr13
Bam_Chr34
Rra_Chr14
Ogl_Chr19
Gan_Chr20
Hca_Chr04
Ped_Chr09
Alu_Chr12
H
B
D
A
C
Chr6 Block2 (248 orthogroups)
Osa_Chr6
Dsi_Chr15
Mba_Chr15
Bam_Chr11
Ola_Chr06
Rgu_Chr06
Mba_Chr18
Dsi_Chr16
Bam_Chr15
Rra_Chr02
Ogl_Chr06
Gan_Chr06
Hca_Chr08
Alu_Chr06
Ped_Chr10
Ogl_Chr19
Gan_Chr20
Mba_Chr13
Rra_Chr14
Dsi_Chr25
Bam_Chr34
Alu_Chr12
Hca_Chr04
Ped_Chr09
A
H
B
D
C
Chr6 Block3 (43 orthogroups)
Osa_Chr6
Rgu_Chr06
Ola_Chr06
Alu_Chr06
Ped_Chr10
Hca_Chr08
Bam_Chr11
Mba_Chr15
Dsi_Chr15
Gan_Chr17
Ogl_Chr14
Bam_Chr15
Dsi_Chr16
Ogl_Chr06
Gan_Chr06
Mba_Chr18
Ped_Chr09
Hca_Chr04
Alu_Chr12
Bam_Chr27
Dsi_Chr26
Rra_Chr18
Mba_Chr30
Rra_Chr02
H
D
A
B
C
Chr6 Block4 (546 orthogroups)
Osa_Chr6
Rgu_Chr06
Ola_Chr06
Rra_Chr02
Gan_Chr06
Ogl_Chr06
Mba_Chr18
Dsi_Chr16
Bam_Chr15
Alu_Chr06
Ped_Chr10
Hca_Chr08
Mba_Chr15
Dsi_Chr15
Bam_Chr11
Alu_Chr12
Ped_Chr09
Hca_Chr04
Rra_Chr11
Gan_Chr18
Ogl_Chr07
Mba_Chr02
Bam_Chr01
Dsi_Chr02
H
B
D
A
C
Chr7 Block1 (104 orthogroups)
Osa_Chr7
Ola_Chr07
Rgu_Chr03
Dsi_Chr18
Mba_Chr27
Bam_Chr21
Rra_Chr13
Ogl_Chr11
Gan_Chr08
Bam_Chr22
Mba_Chr06
Dsi_Chr20
Mba_Chr24
Dsi_Chr03
Bam_Chr26
Hca_Chr11
Ped_Chr11
Alu_Chr13
Rra_Chr22
Ogl_Chr20
Gan_Chr23
Hca_Chr21
Alu_Chr18
Ped_Chr15
H
B
A
C
D
C

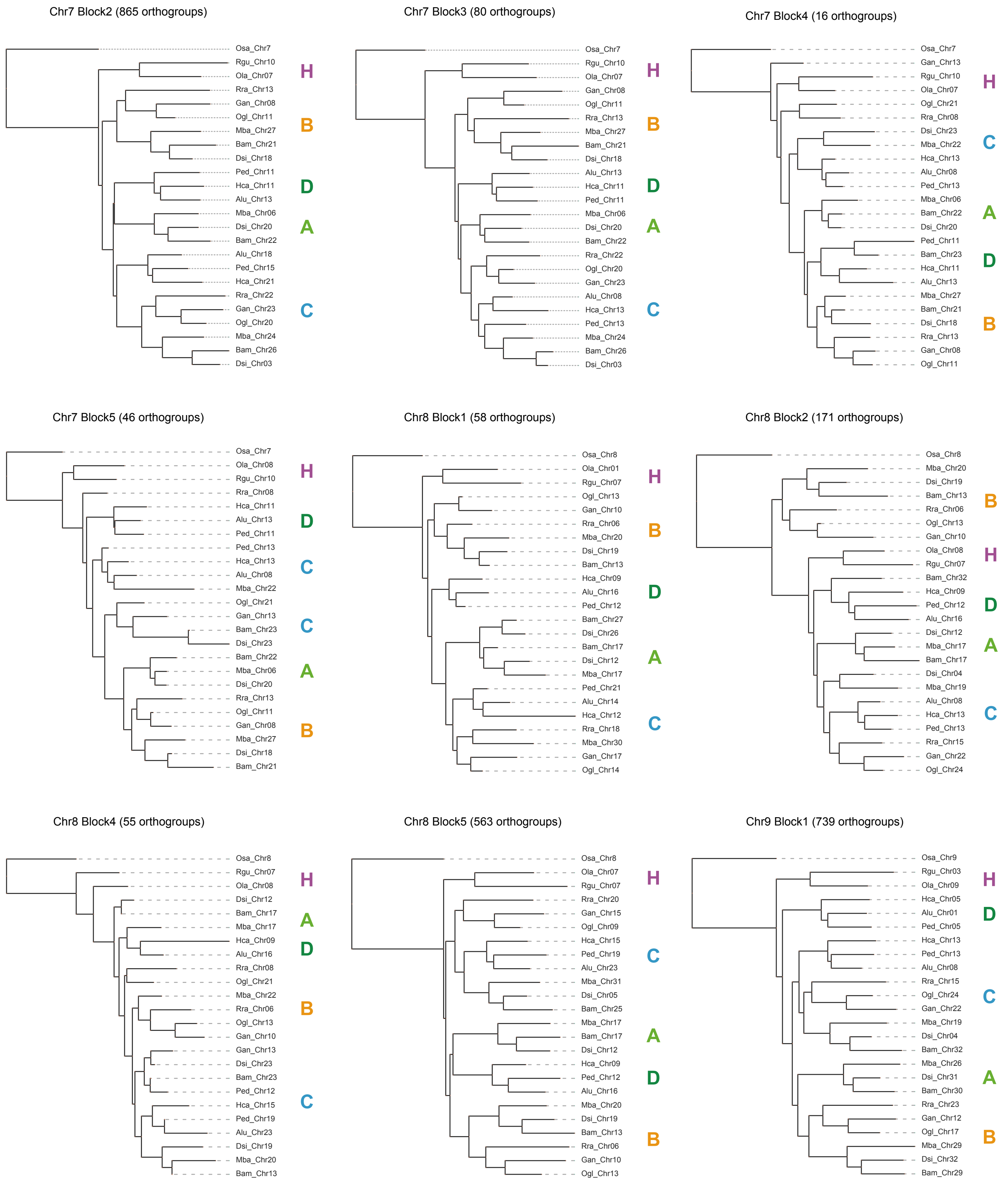
Chr7 Block2 (865 orthogroups)
Osa_Chr7
Rgu_Chr10
Ola_Chr07
Rra_Chr13
Gan_Chr08
Ogl_Chr11
Mba_Chr27
Bam_Chr21
Dsi_Chr18
Ped_Chr11
Hca_Chr11
Alu_Chr13
Mba_Chr06
Dsi_Chr20
Bam_Chr22
Alu_Chr18
Ped_Chr15
Hca_Chr21
Rra_Chr22
Gan_Chr23
Ogl_Chr20
Mba_Chr24
Bam_Chr26
Dsi_Chr03
H
B
D
A
C
Chr7 Block3 (80 orthogroups)
Osa_Chr7
Rgu_Chr10
Ola_Chr07
Gan_Chr08
Ogl_Chr11
Rra_Chr13
Mba_Chr27
Bam_Chr21
Dsi_Chr18
Alu_Chr13
Hca_Chr11
Ped_Chr11
Mba_Chr06
Dsi_Chr20
Bam_Chr22
Rra_Chr22
Ogl_Chr20
Gan_Chr23
Alu_Chr08
Hca_Chr13
Ped_Chr13
Mba_Chr24
Bam_Chr26
Dsi_Chr03
H
B
D
A
C
Chr7 Block4 (16 orthogroups)
Osa_Chr7
Gan_Chr13
Rgu_Chr10
Ola_Chr07
Ogl_Chr21
Rra_Chr08
Dsi_Chr23
Mba_Chr22
Hca_Chr13
Alu_Chr08
Ped_Chr13
Mba_Chr06
Bam_Chr22
Dsi_Chr20
Ped_Chr11
Bam_Chr23
Hca_Chr11
Alu_Chr13
Mba_Chr27
Bam_Chr21
Dsi_Chr18
Rra_Chr13
Gan_Chr08
Ogl_Chr11
H
C
A
D
B
Chr7 Block5 (46 orthogroups)
Osa_Chr7
Ola_Chr08
Rgu_Chr10
Rra_Chr08
Hca_Chr11
Alu_Chr13
Ped_Chr11
Ped_Chr13
Hca_Chr13
Alu_Chr08
Mba_Chr22
Ogl_Chr21
Gan_Chr13
Bam_Chr23
Dsi_Chr23
Bam_Chr22
Mba_Chr06
Dsi_Chr20
Rra_Chr13
Ogl_Chr11
Gan_Chr08
Mba_Chr27
Dsi_Chr18
Bam_Chr21
H
D
C
C
A
B
Chr8 Block1 (58 orthogroups)
Osa_Chr8
Ola_Chr01
Rgu_Chr07
Ogl_Chr13
Gan_Chr10
Rra_Chr06
Mba_Chr20
Dsi_Chr19
Bam_Chr13
Hca_Chr09
Alu_Chr16
Ped_Chr12
Bam_Chr27
Dsi_Chr26
Bam_Chr17
Dsi_Chr12
Mba_Chr17
Ped_Chr21
Alu_Chr14
Hca_Chr12
Rra_Chr18
Mba_Chr30
Gan_Chr17
Ogl_Chr14
H
B
D
A
C
Chr8 Block2 (171 orthogroups)
Osa_Chr8
Mba_Chr20
Dsi_Chr19
Bam_Chr13
Rra_Chr06
Ogl_Chr13
Gan_Chr10
Ola_Chr08
Rgu_Chr07
Bam_Chr32
Hca_Chr09
Ped_Chr12
Alu_Chr16
Dsi_Chr12
Mba_Chr17
Bam_Chr17
Dsi_Chr04
Mba_Chr19
Alu_Chr08
Hca_Chr13
Ped_Chr13
Rra_Chr15
Gan_Chr22
Ogl_Chr24
B
H
D
A
C
Chr8 Block4 (55 orthogroups)
Osa_Chr8
Rgu_Chr07
Ola_Chr08
Dsi_Chr12
Bam_Chr17
Mba_Chr17
Hca_Chr09
Alu_Chr16
Rra_Chr08
Ogl_Chr21
Mba_Chr22
Rra_Chr06
Ogl_Chr13
Gan_Chr10
Gan_Chr13
Dsi_Chr23
Bam_Chr23
Ped_Chr12
Hca_Chr15
Ped_Chr19
Alu_Chr23
Dsi_Chr19
Mba_Chr20
Bam_Chr13
H
A
D
B
C
Chr8 Block5 (563 orthogroups)
Osa_Chr8
Ola_Chr07
Rgu_Chr07
Rra_Chr20
Gan_Chr15
Ogl_Chr09
Hca_Chr15
Ped_Chr19
Alu_Chr23
Mba_Chr31
Dsi_Chr05
Bam_Chr25
Mba_Chr17
Bam_Chr17
Dsi_Chr12
Hca_Chr09
Ped_Chr12
Alu_Chr16
Mba_Chr20
Dsi_Chr19
Bam_Chr13
Rra_Chr06
Gan_Chr10
Ogl_Chr13
H
C
A
D
B
Chr9 Block1 (739 orthogroups)
Osa_Chr9
Rgu_Chr03
Ola_Chr09
Hca_Chr05
Alu_Chr01
Ped_Chr05
Hca_Chr13
Ped_Chr13
Alu_Chr08
Rra_Chr15
Ogl_Chr24
Gan_Chr22
Mba_Chr19
Dsi_Chr04
Bam_Chr32
Mba_Chr26
Dsi_Chr31
Bam_Chr30
Rra_Chr23
Gan_Chr12
Ogl_Chr17
Mba_Chr29
Dsi_Chr32
Bam_Chr29
H
D
C
A
B

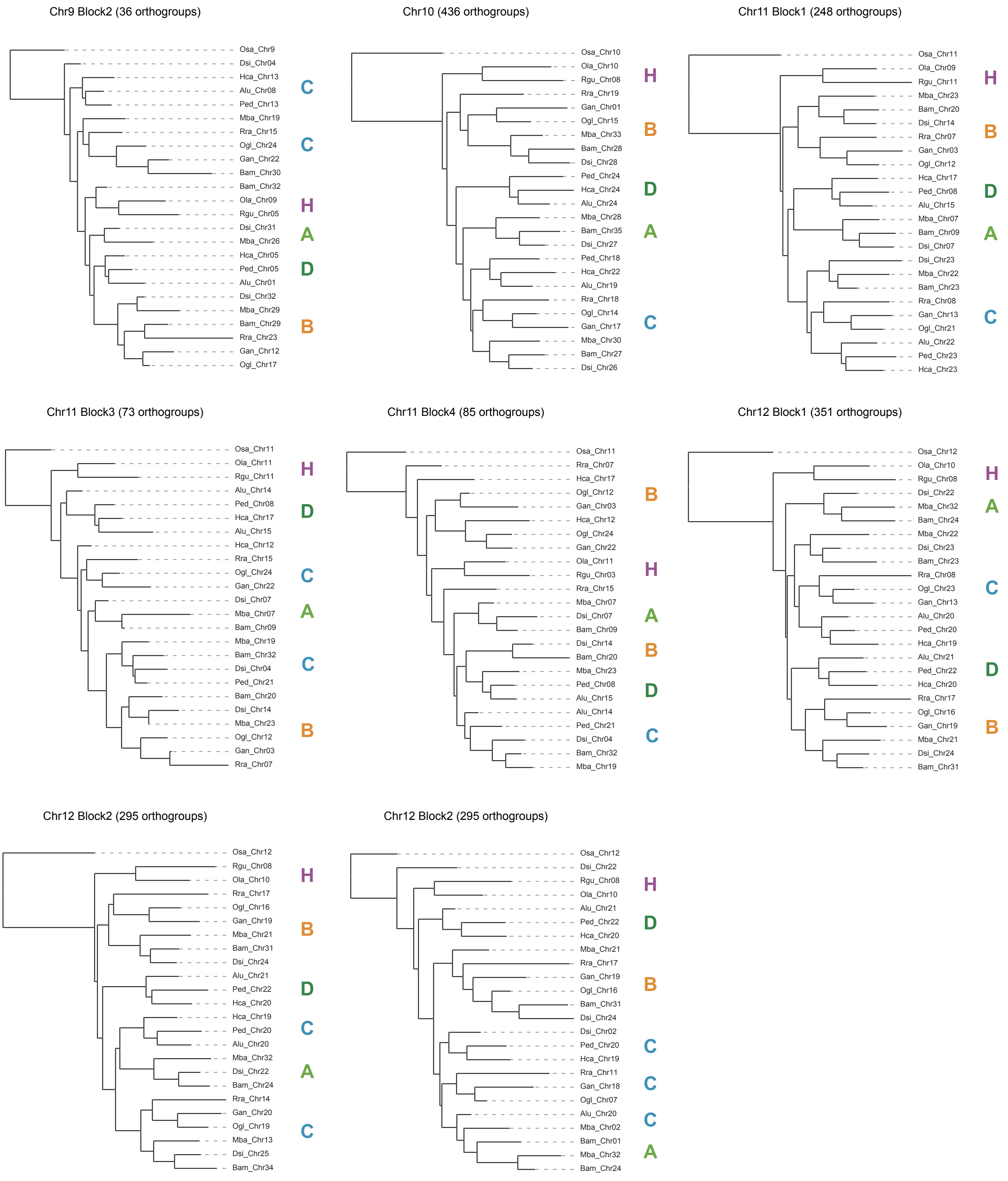

Supplementary Fig. 8. Identification of the subgenomes in woody bamboos based on phylogenetic topologies inferred by a total of 13,891 "low-copy" syntenic gene orthogroups from 35 syntenic blocks spanning the whole genomes. The phylogenetic tree of each syntenic block is shown. The tip labels are represented by the abbreviation of the species name and the initial pseudo-chromosome number constructed by Hi-C connected by an underline. Each identified clade is marked by its comprising subgenome.

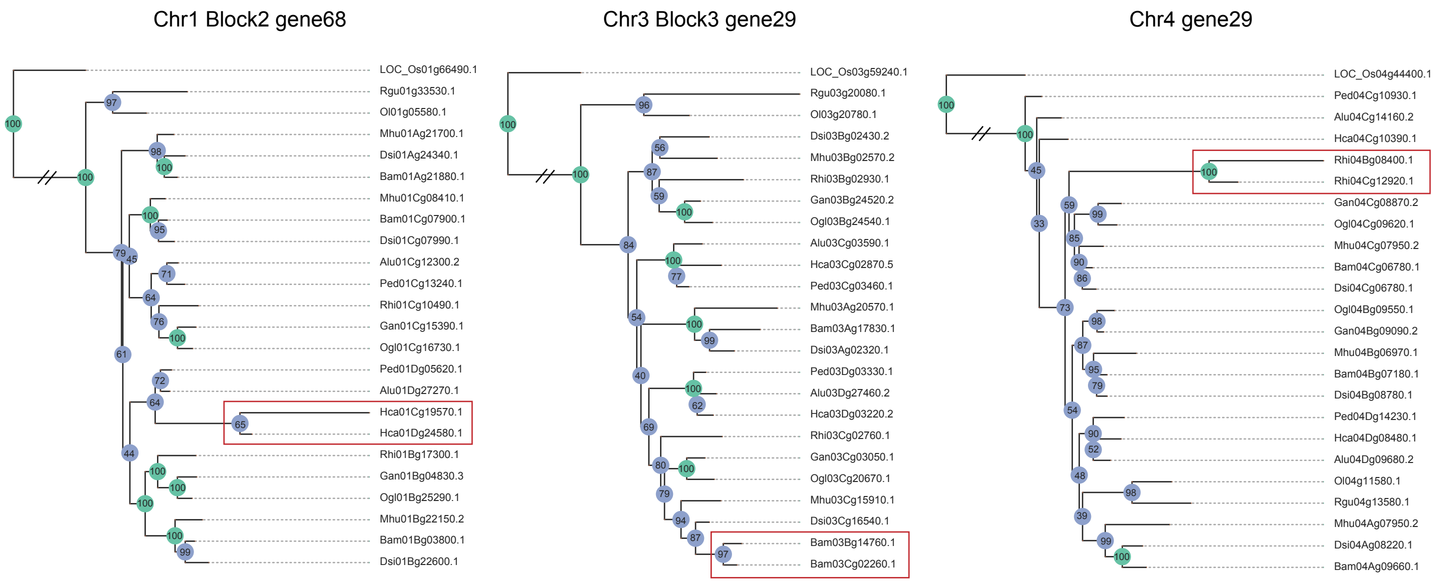

Supplementary Fig. 9. Examples of putative gene conversion in red rectangle observed for the individual gene tree in the 456 "perfect-copy" syntenic gene data set.

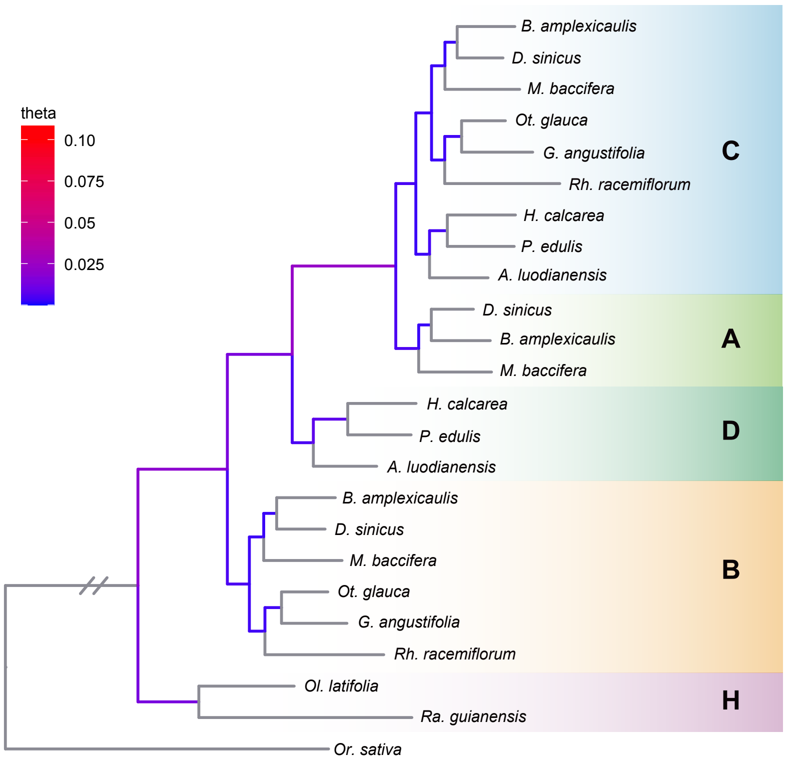

Supplementary Fig. 10. Assessment of impact of incomplete lineage sorting (ILS) on the observed discordance among gene trees. Phylogenetic tree topology inferred from the 430 gene data set is colored by the inferred theta parameter, which is computed by dividing the mutation units from RAxML analyses and coalescent units from ASTRAL analyses for each internal branch. The grey color represents branches with a lack of data to infer theta.

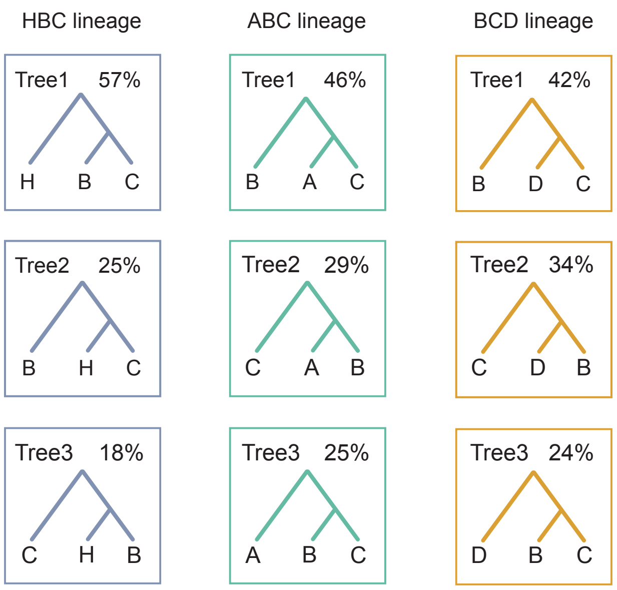

Supplementary Fig. 11. The proportions of gene tree topologies regarding to relationships among three clades for the 2,021 genes. The value in the top right corner is the percentage of all 2,021 genes recovered for each topology.

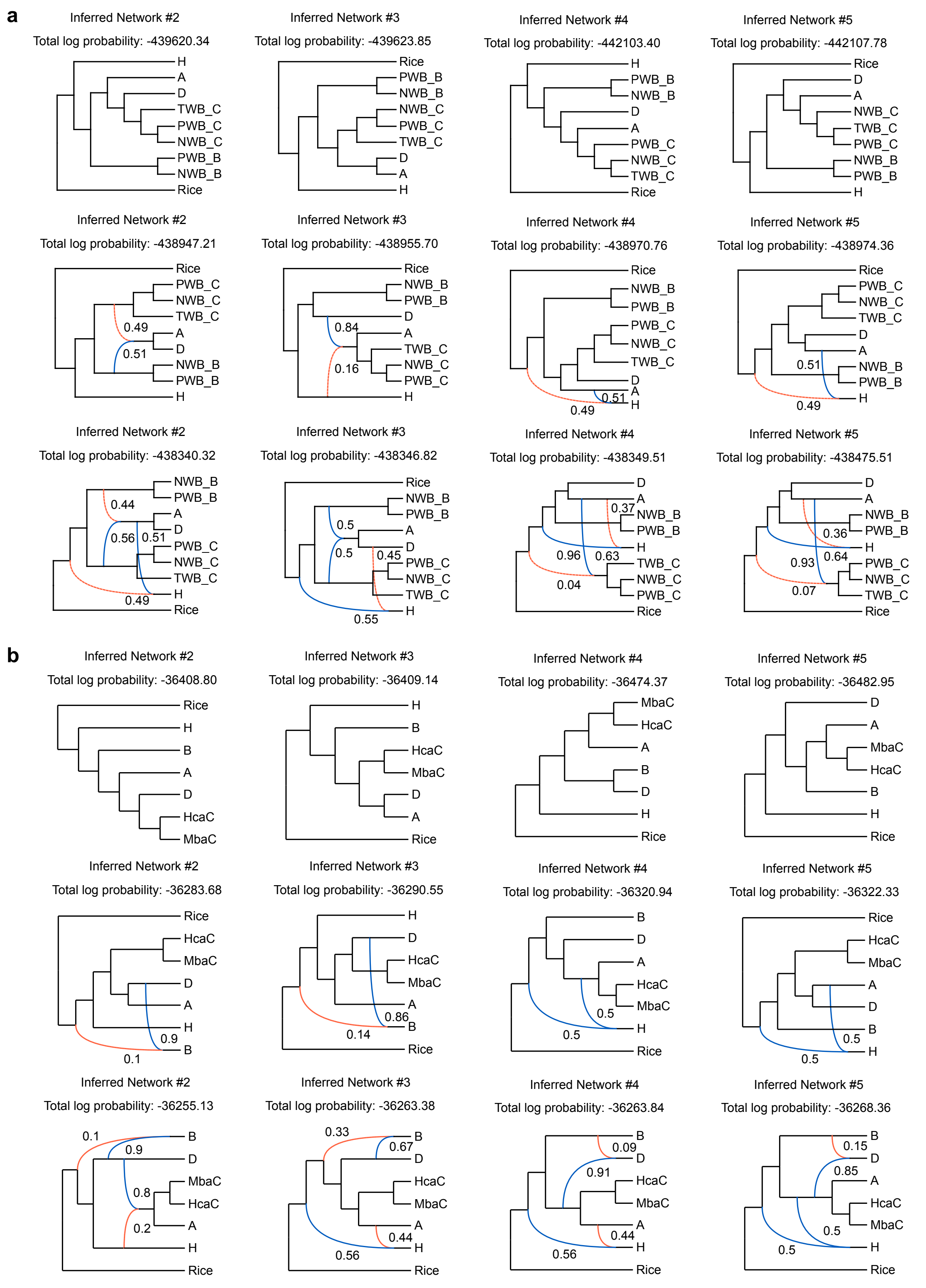

Supplementary Fig. 12. Main hybridization scenarios among different bamboo ancestors revealed by Network analyses. Four of five networks (networks 2-5 shown here and network 1 in Extended Data Fig. 3e,f) assuming zero, one, and two events of hybridization in the presence of ILS are inferred by PhyloNet analyses of both 430 **(a)** and 2,021 **(b)** genes. The log probability for each network is shown. Solid (blue) and dashed (red) curved lines indicate the major and minor edges that contribute to the hybrid descendants, and the numbers next to them indicate the inheritance probabilities of each parent.

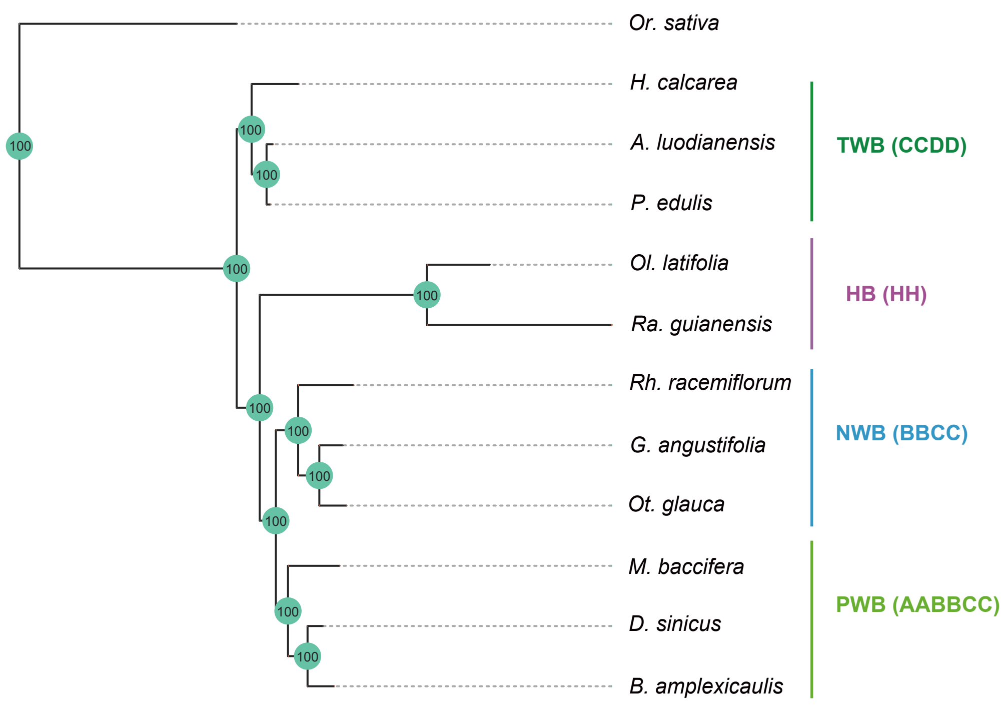

Supplementary Fig. 13. Phylogenetic tree inferred from plastome sequences of 11 sampled bamboos. The non-monophyly of woody bamboos were revealed with bootstrap values inferred from RAxML analyses shown.

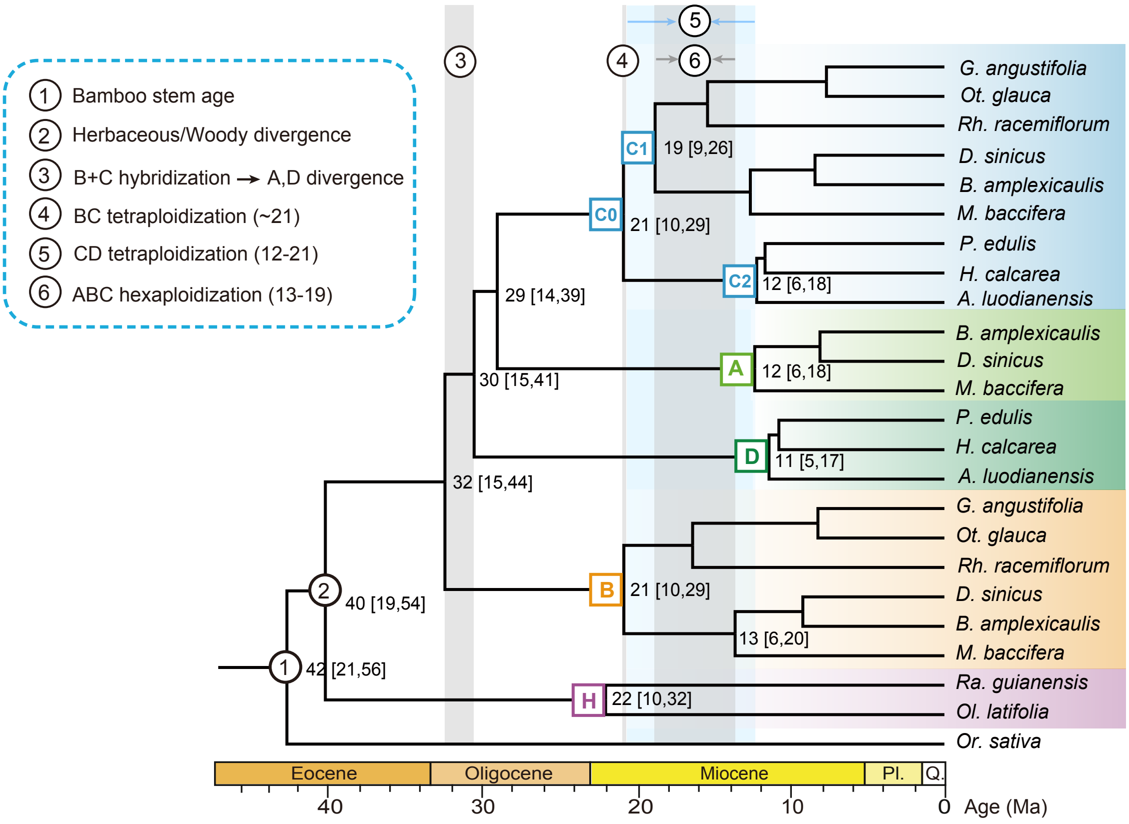

Supplementary Fig. 14. Divergence time estimation of major bamboo lineages. The medium divergence time (million years ago, Ma) with 95% highest posterior density (HPD) interval are presented at nodes. Major evolutionary events are shown in dashed boxes and the corresponding time range is highlighted by shaded color columns.

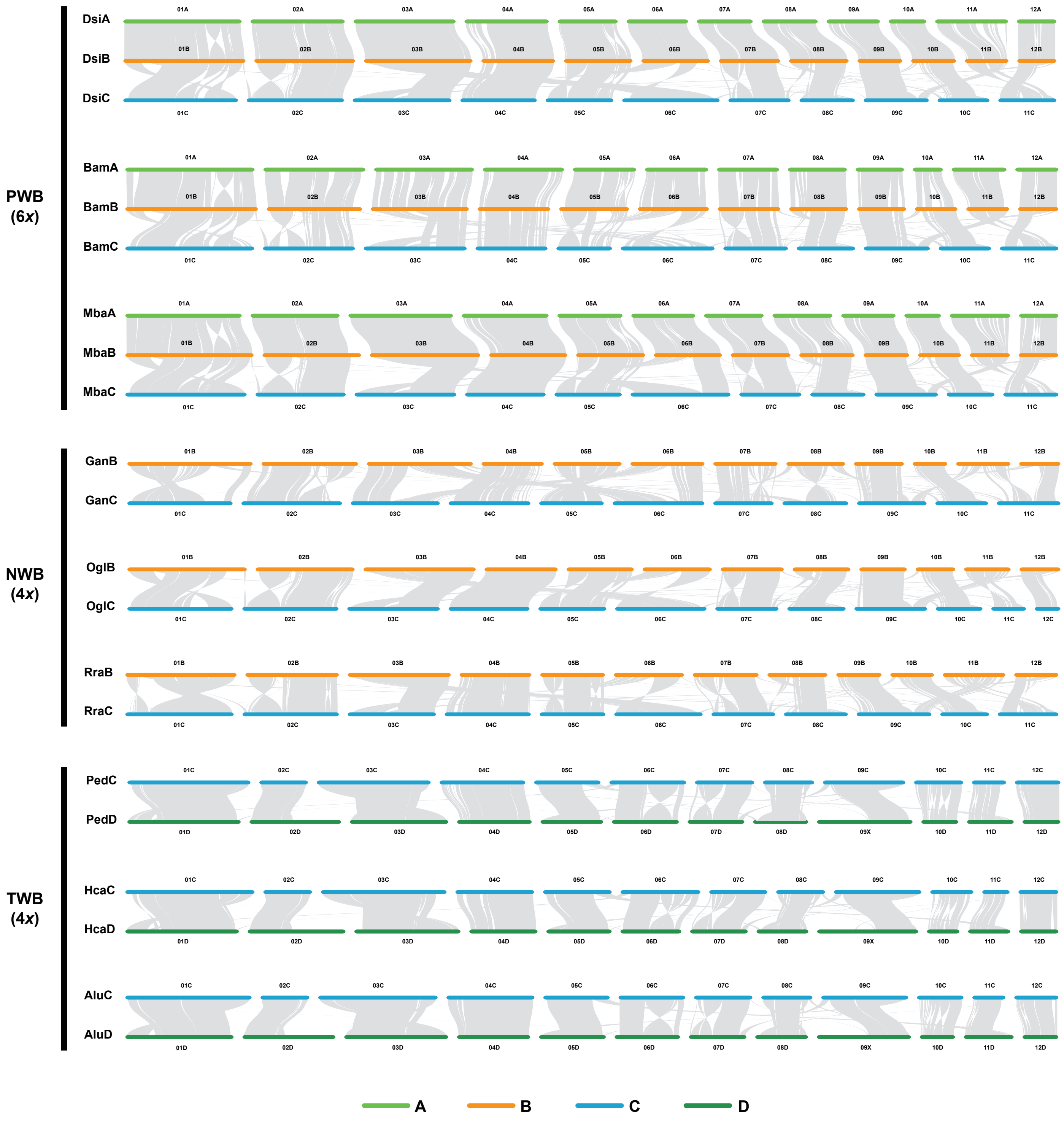

Supplementary Fig. 15. The conserved synteny between subgenomes within the genome of woody bamboos.

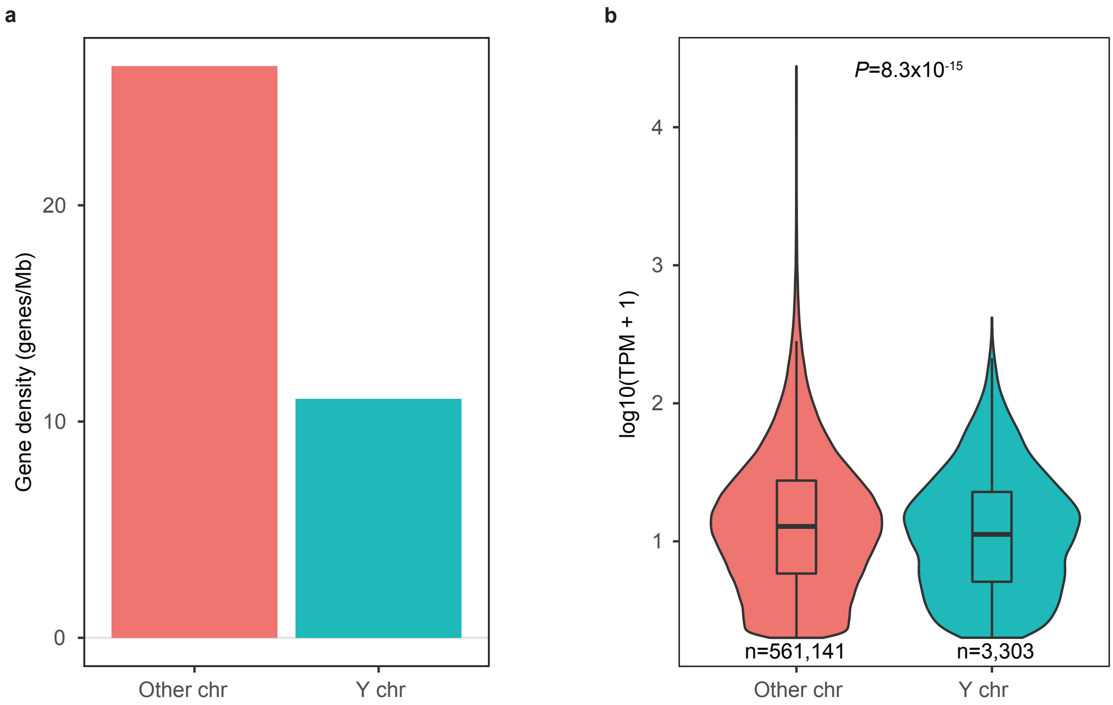

Supplementary Fig. 16. Comparison of gene density (a) and expression level (b) of the possible B chromosome to other 23 chromosomes in *Rh. racemiflorum*. Boxplots: centerline, median; box limits, first and third quartiles; whisker, 1.5x interquartile range; two-sided Wilcoxon rank-sum test.

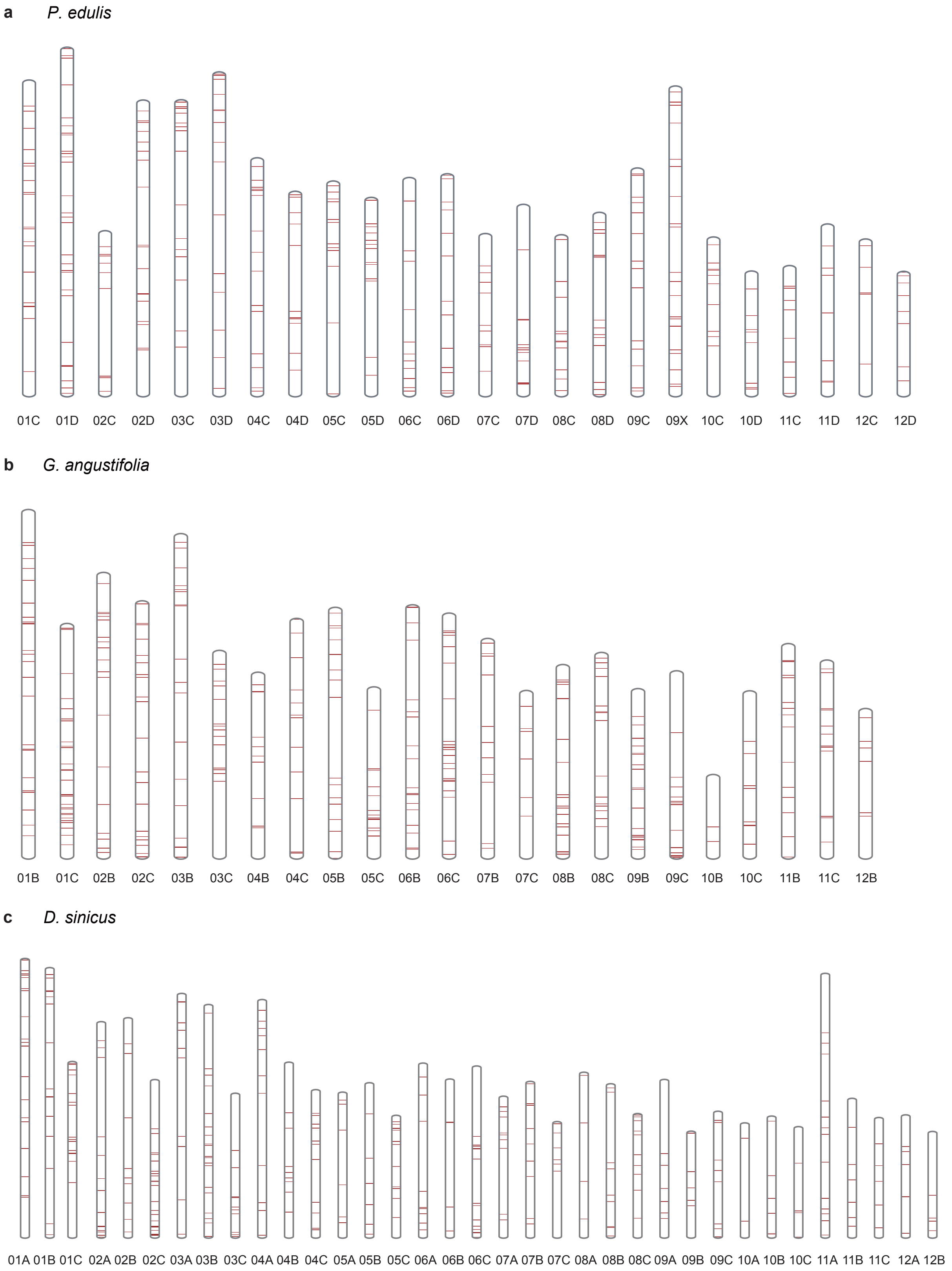

Supplementary Fig. 17. Distribution of putative homoeologous exchanges in each chromosome of three representative species of *P. edulis*, *G. angustifolia* and *D. sinicus*.

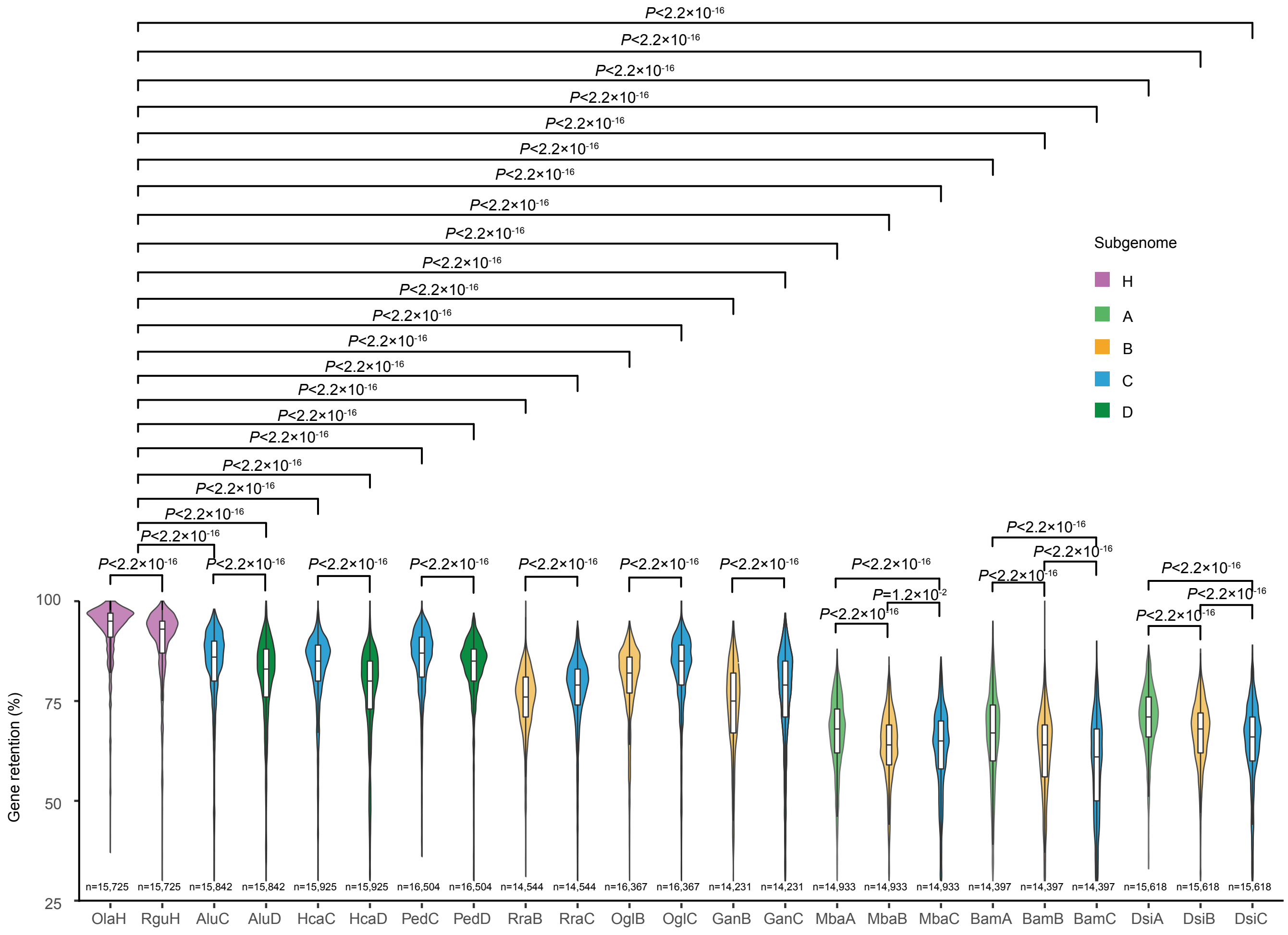

Supplementary Fig. 18. Gene retention patterns among 11 bamboo genomes with the rice genome as reference. The boxplots (centerline, median; box limits, first and third quartiles; whisker, 1.5x interquartile range; two-sided Wilcoxon rank-sum test) were generated using the percentage of retained genes estimated from all bamboo chromosomes.

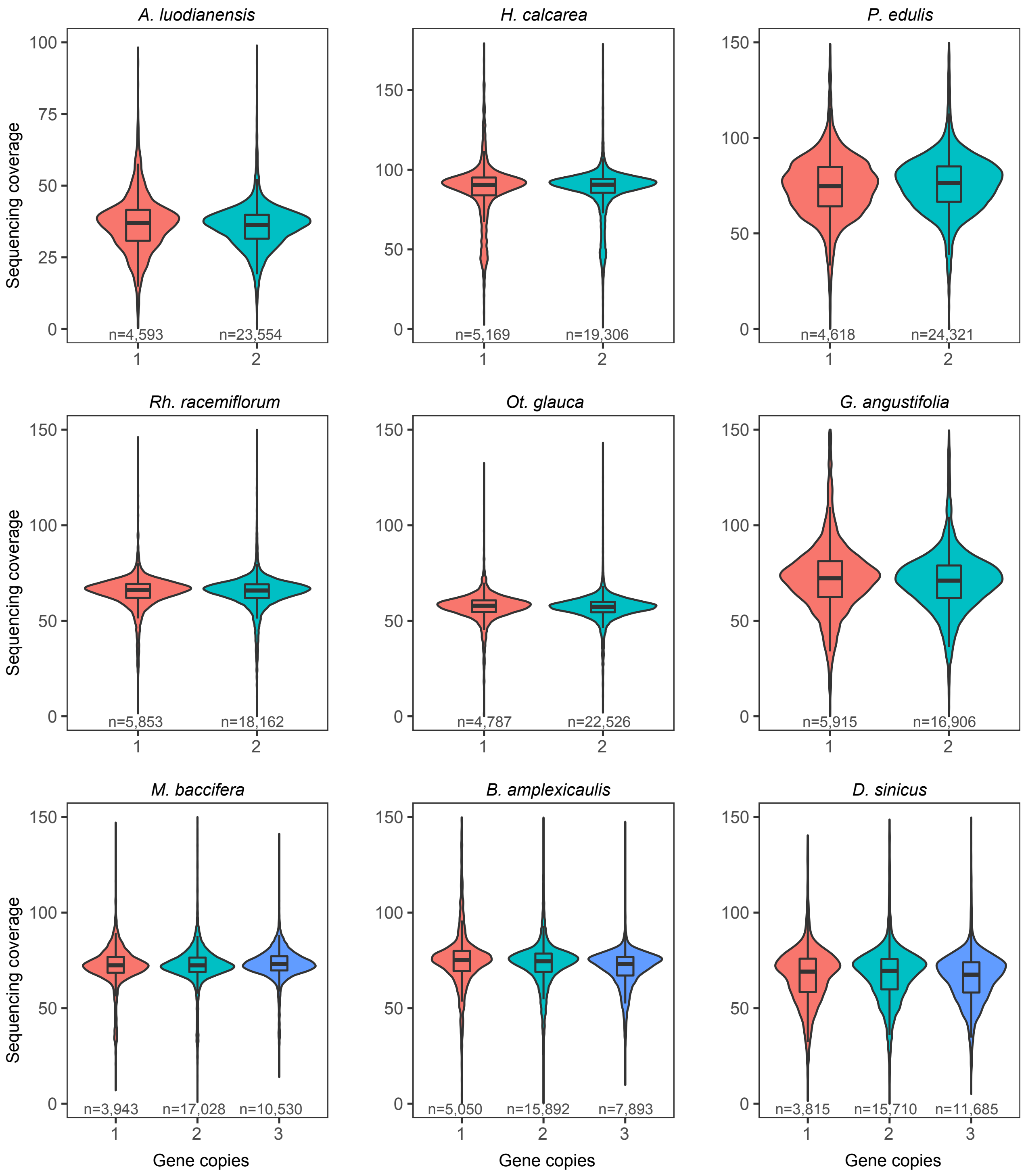

Supplementary Fig. 19. The sequencing coverage of genes retained in single and two copies (for tetraploids) or single, two and three copies (for hexaploids) between subgenomes in nine woody bamboo genomes. The coverage was calculated by mapping the short sequencing reads to the genome assemblies. Boxplots: centerline, median; box limits, first and third quartiles; whisker, 1.5x interquartile range.

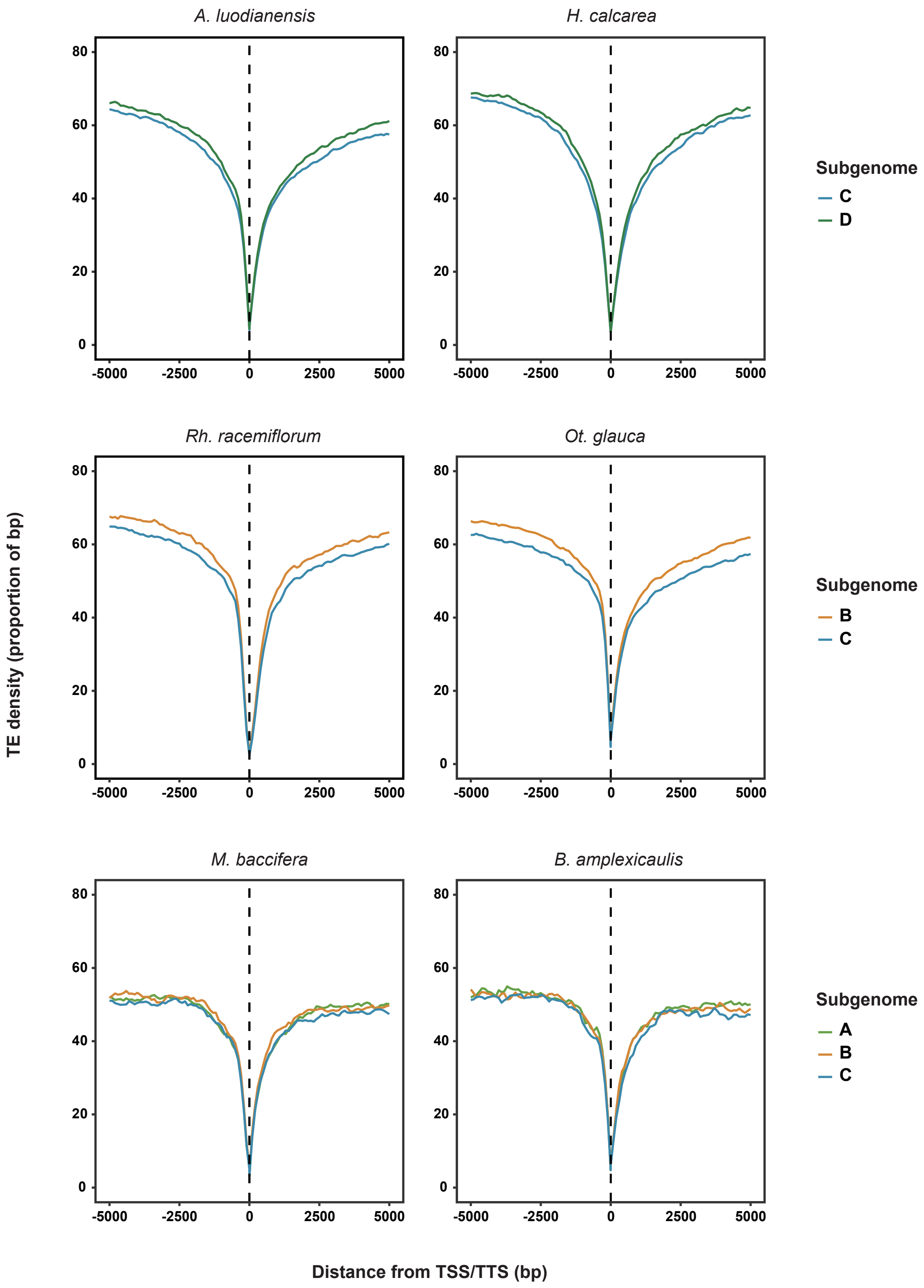

Supplementary Fig. 20. TE landscape surrounding genes among subgenomes in nine woody bamboos. The dashed line indicates the transcriptional start (TSS)/termination (TTS) site. Significant differences (P<0.001) were observed in all comparisons, except for the subgenome A and B comparison in *M. baccifera* and *B. amplexicaulis*.

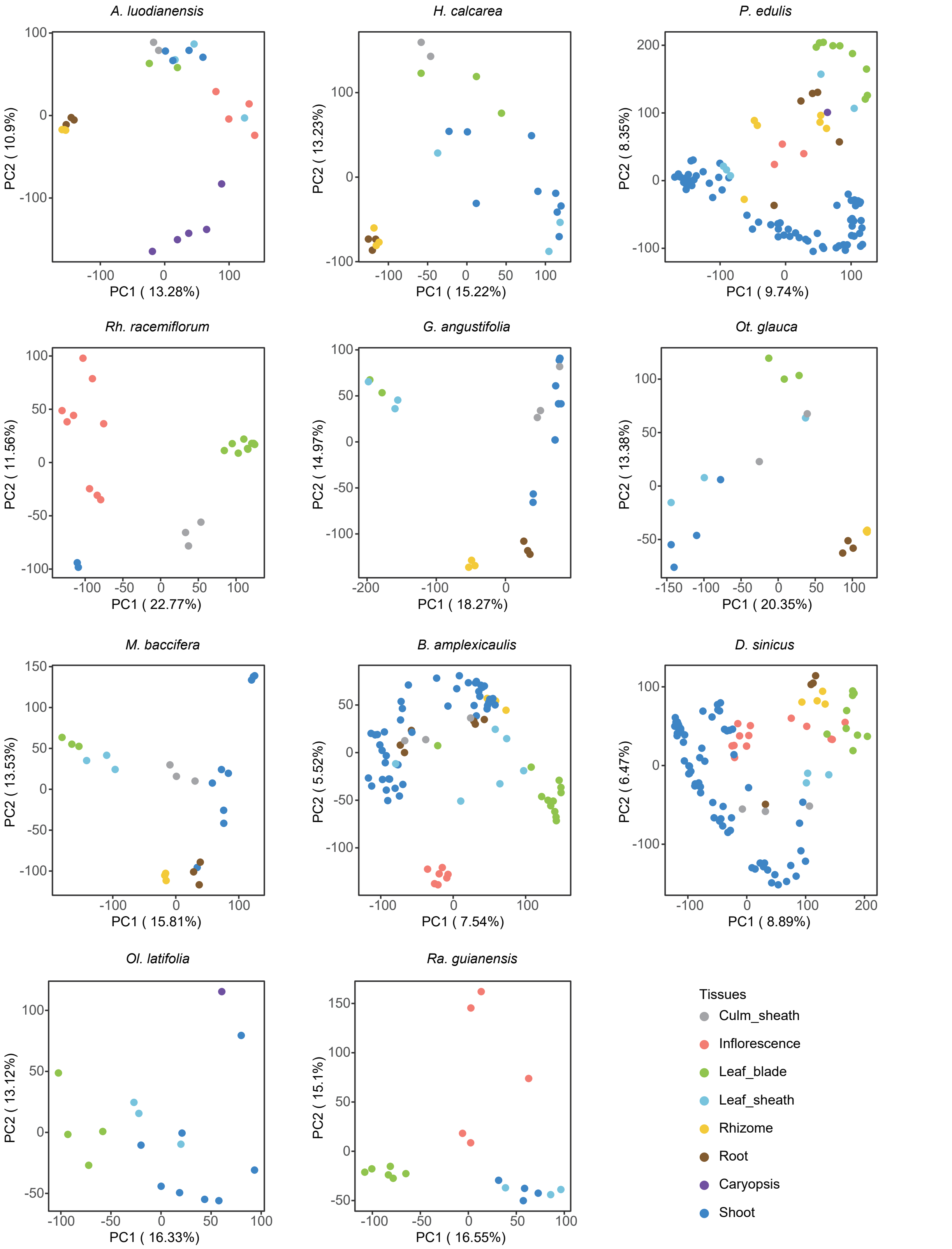

Supplementary Fig. 21. Principal component analysis (PCA) of all 476 transcriptome samples in 11 bamboo species. The log2-transformed expression values are used for analysis with samples colored according to their derived tissues.

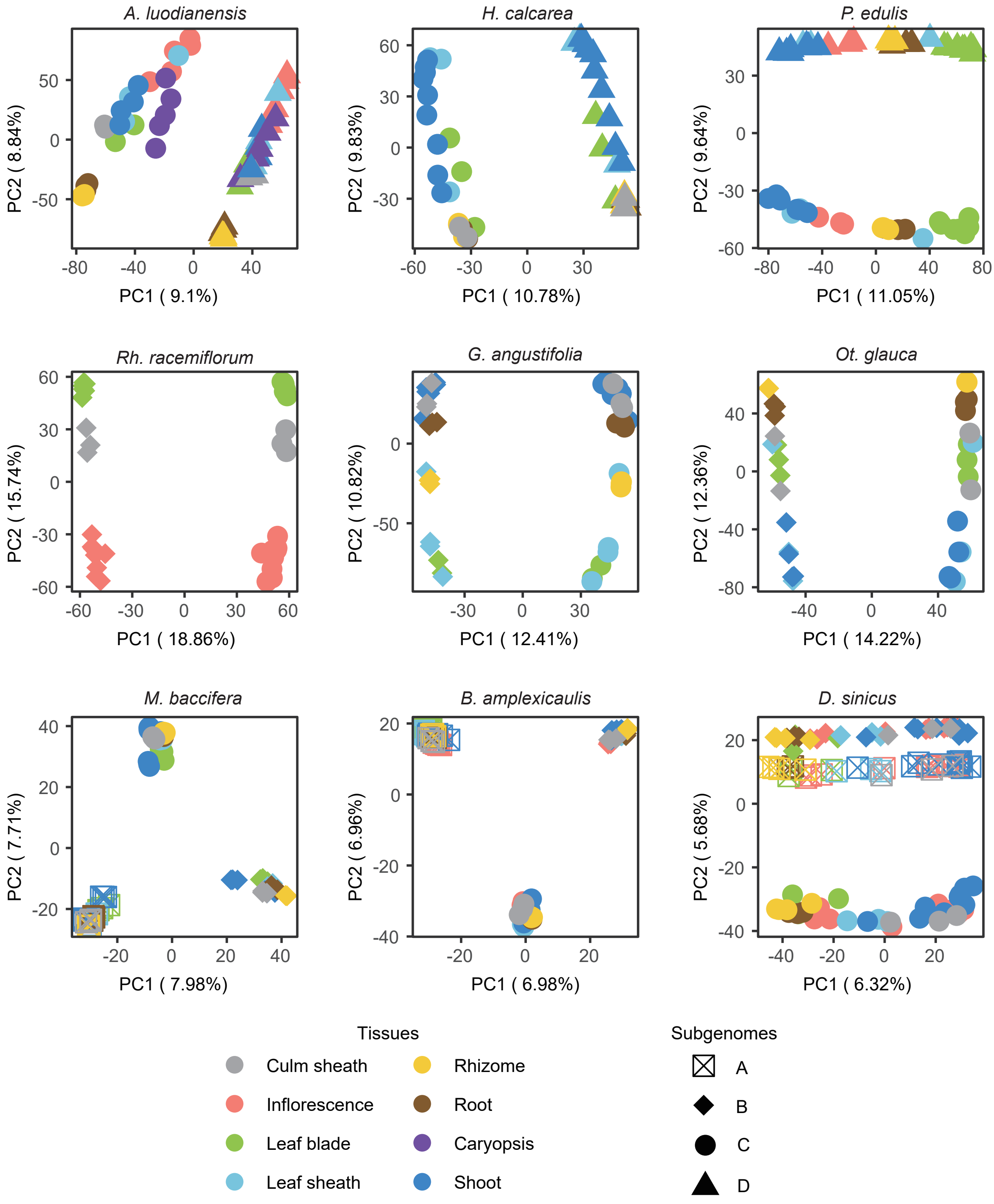

Supplementary Fig. 22. Principal component analysis (PCA) plots for expression similarity of subgenomes of woody bamboos. Samples colored and shaped according to their tissues and subgenomes, respectively.

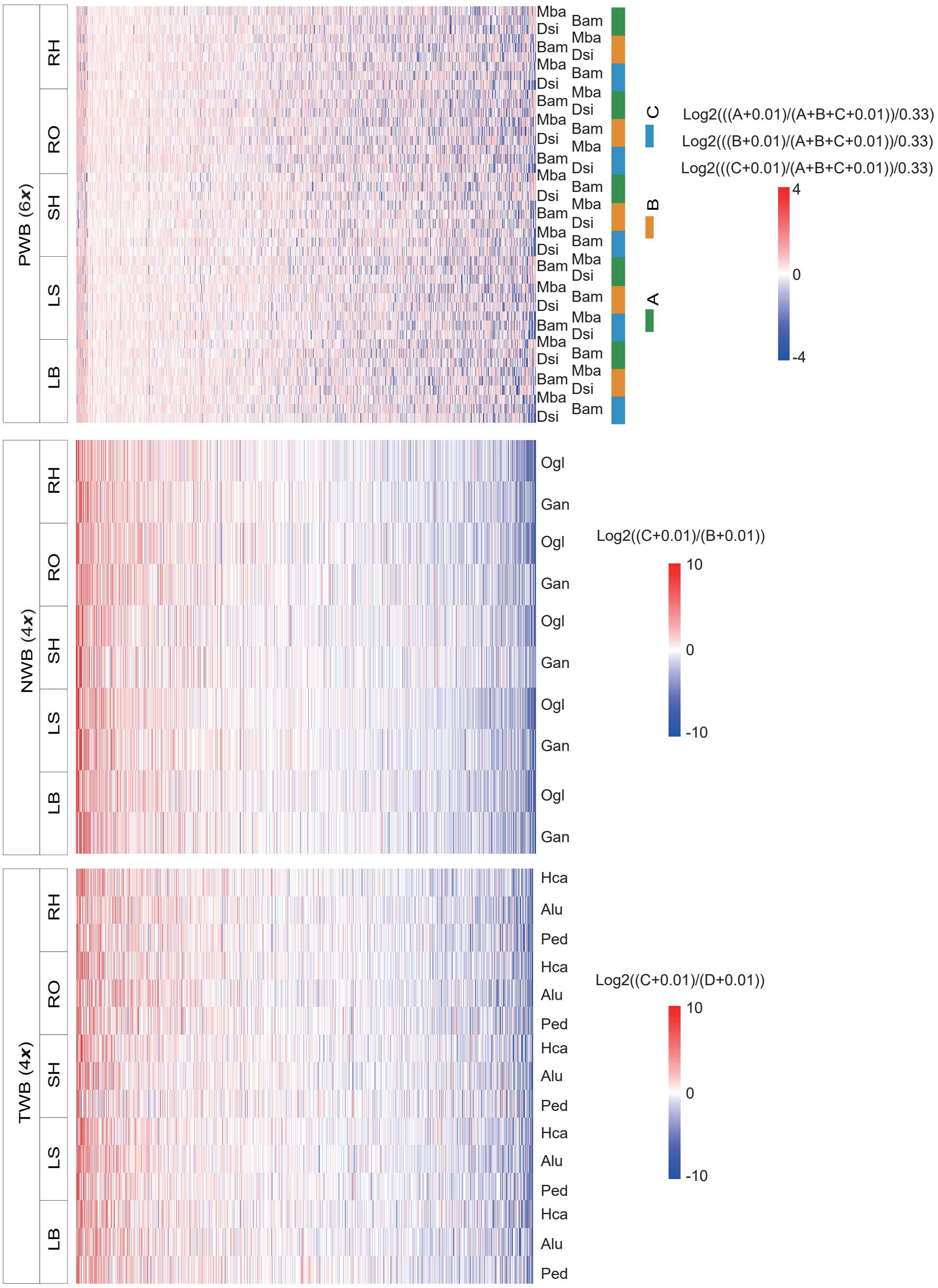

Supplementary Fig. 23. The heat map of expression of homoeologs across five tissues sampled in all the three woody bamboo clades. For the hexaploids, dominance is shown in red and suppressed in blue. For the tetraploids, the expression level of B > C/D > C and C > D/C > B is shown in blue and red, respectively. Leaf blade, LB; leaf sheath, LS; shoot, SH; root, RO; and rhizome, RH.

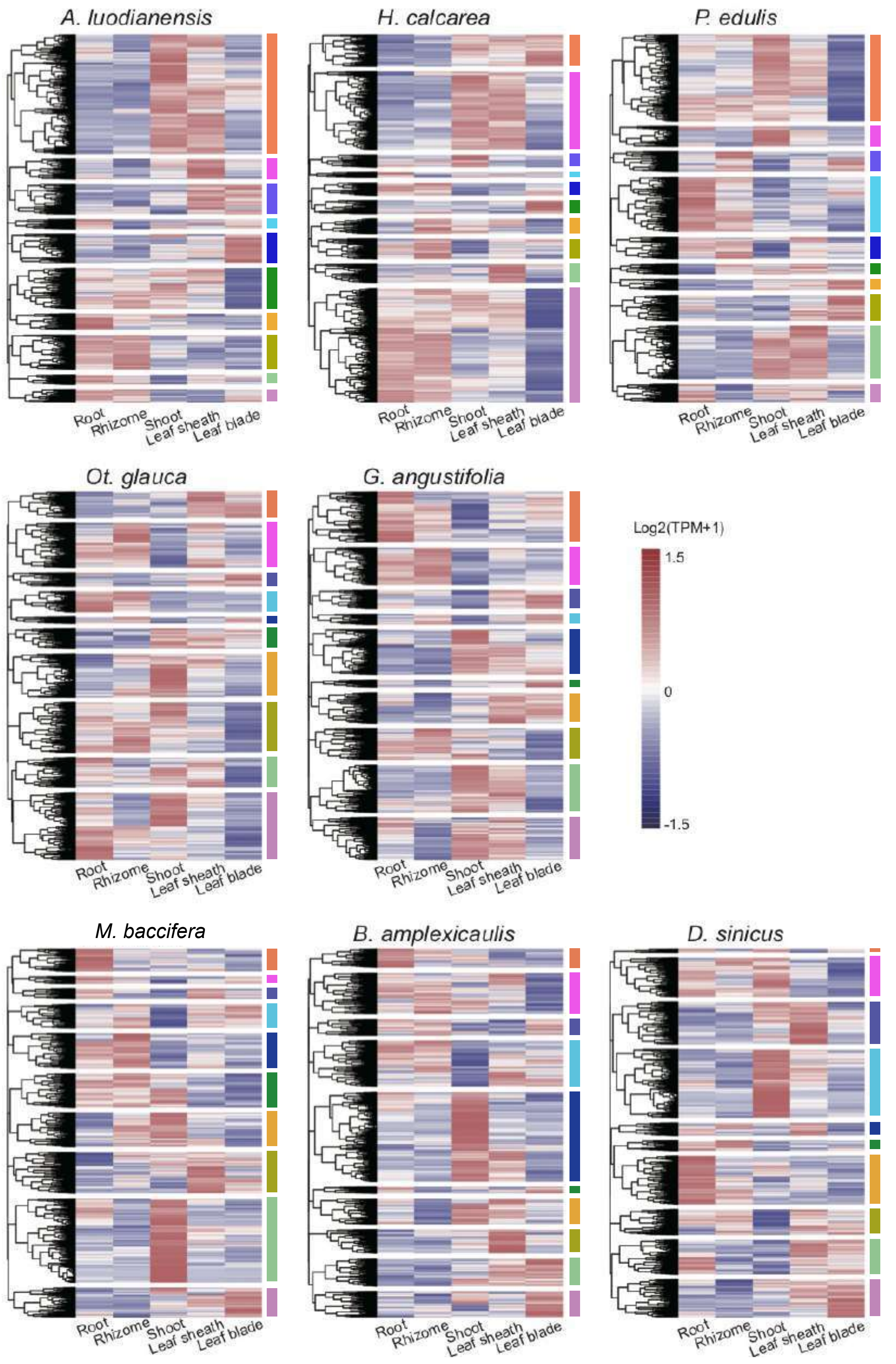

Supplementary Fig. 24. The correlation of gene expression between homoeologs across five tissues in woody bamboos. Genes were clustered into 10 groups indicated by color bars for the 4,123, 3,839 and 1,157 clade-shared homoeologous pairs/triads in TWBs, NWBs and PWBs, respectively.

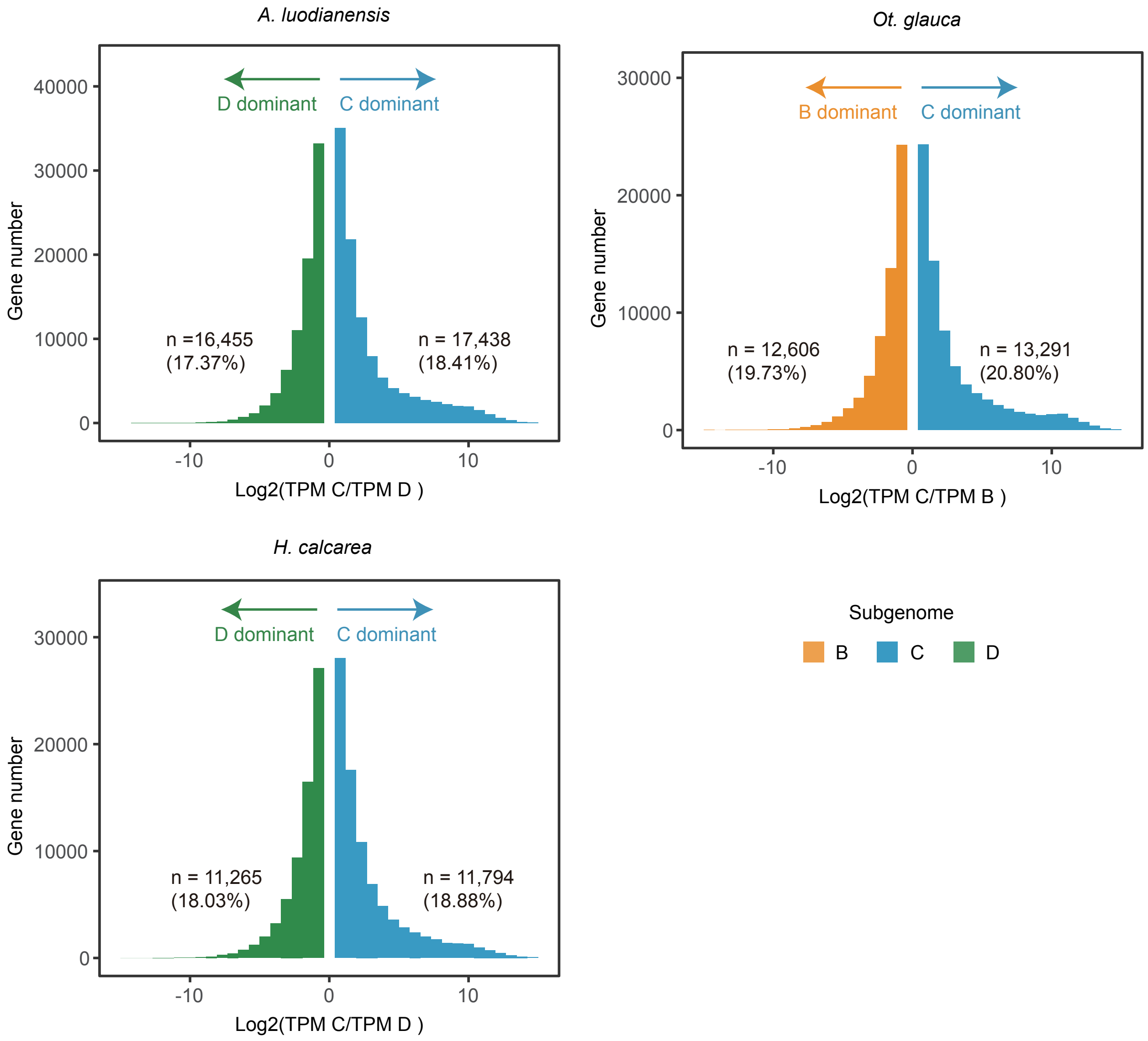

Supplementary Fig. 25. Distributions of genes with biased expression in tetraploid bamboos. The n value indicates the total number of differentially expressed genes under the criteria of |Log2-fold changes (FC)| ≥ 1 and Benjamini-Hochberg adjusted *P* value ≤ 0.05 (two-sided Wald test) for all homoeologous pairs within each species from all sampled tissues.

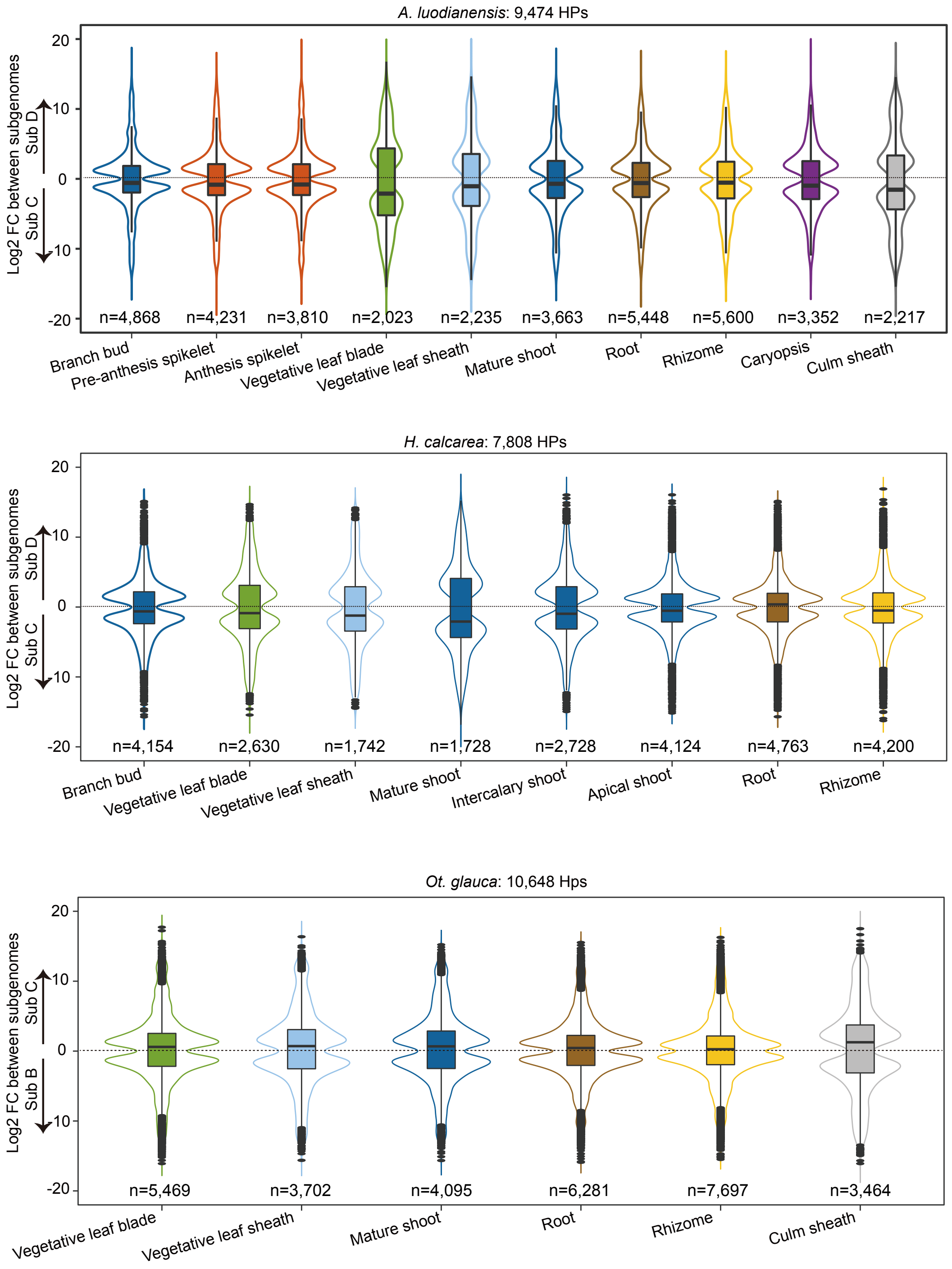

Supplementary Fig. 26. Boxplots of biased expression for homoeologous pairs across different tissues in tetraploid bamboos. Log2-fold changes (FC) in biased pairwise gene expression for all homoeologous pairs (HPs) within each species across different tissues. Boxplots: centerline, median; box limits, first and third quartiles; whisker, 1.5x interquartile range.

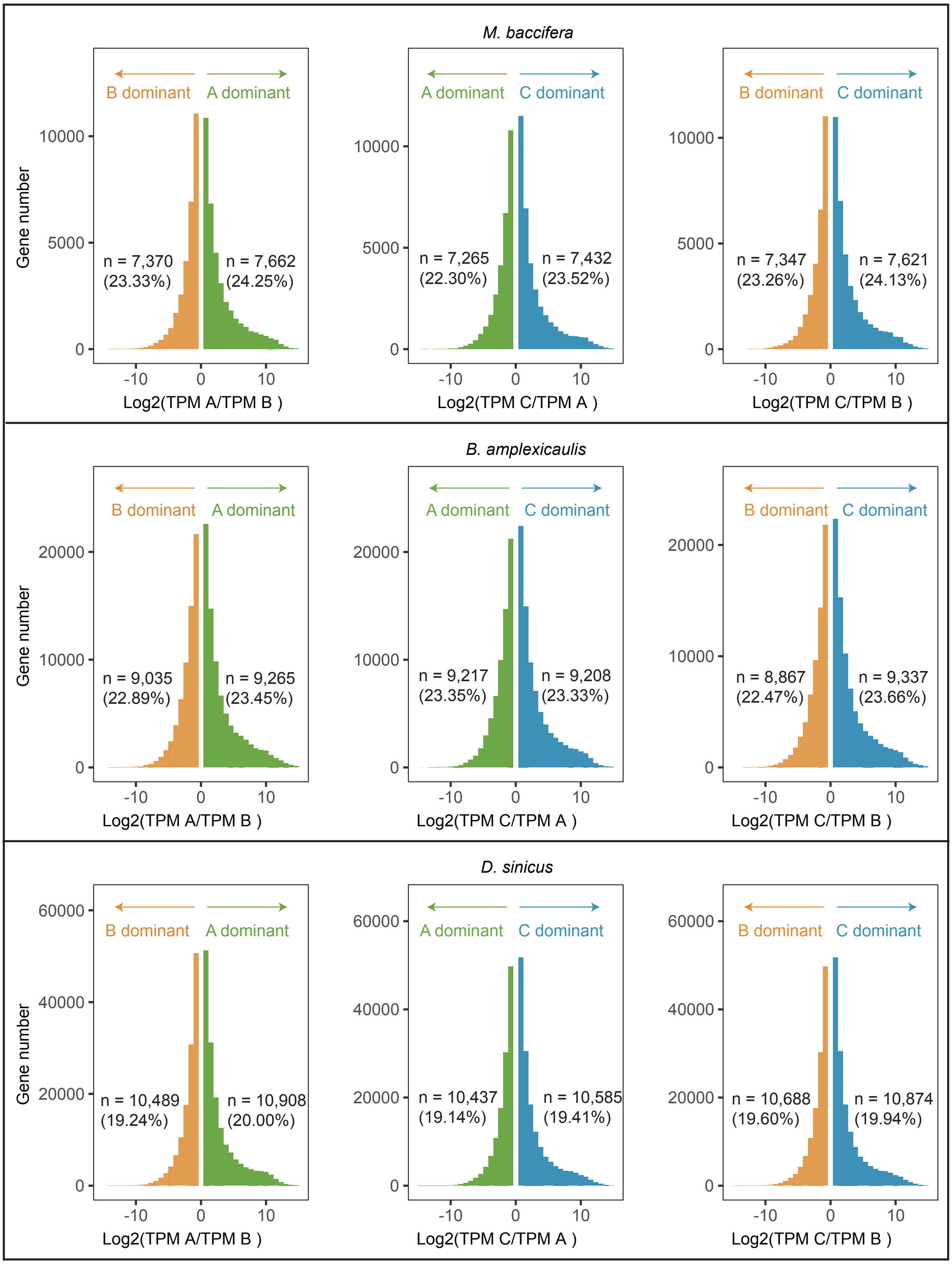

Supplementary Fig. 27. Distributions of genes with biased expression in hexaploid bamboos. The n value indicates the total number of differentially expressed genes in pairwise comparison under the criteria of |Log2-fold changes (FC)| ≥ 1and Benjamini-Hochberg adjusted *P* value ≤ 0.05 (two-sided Wald test) for all homoeologous triads within each species for all sampled tissues.

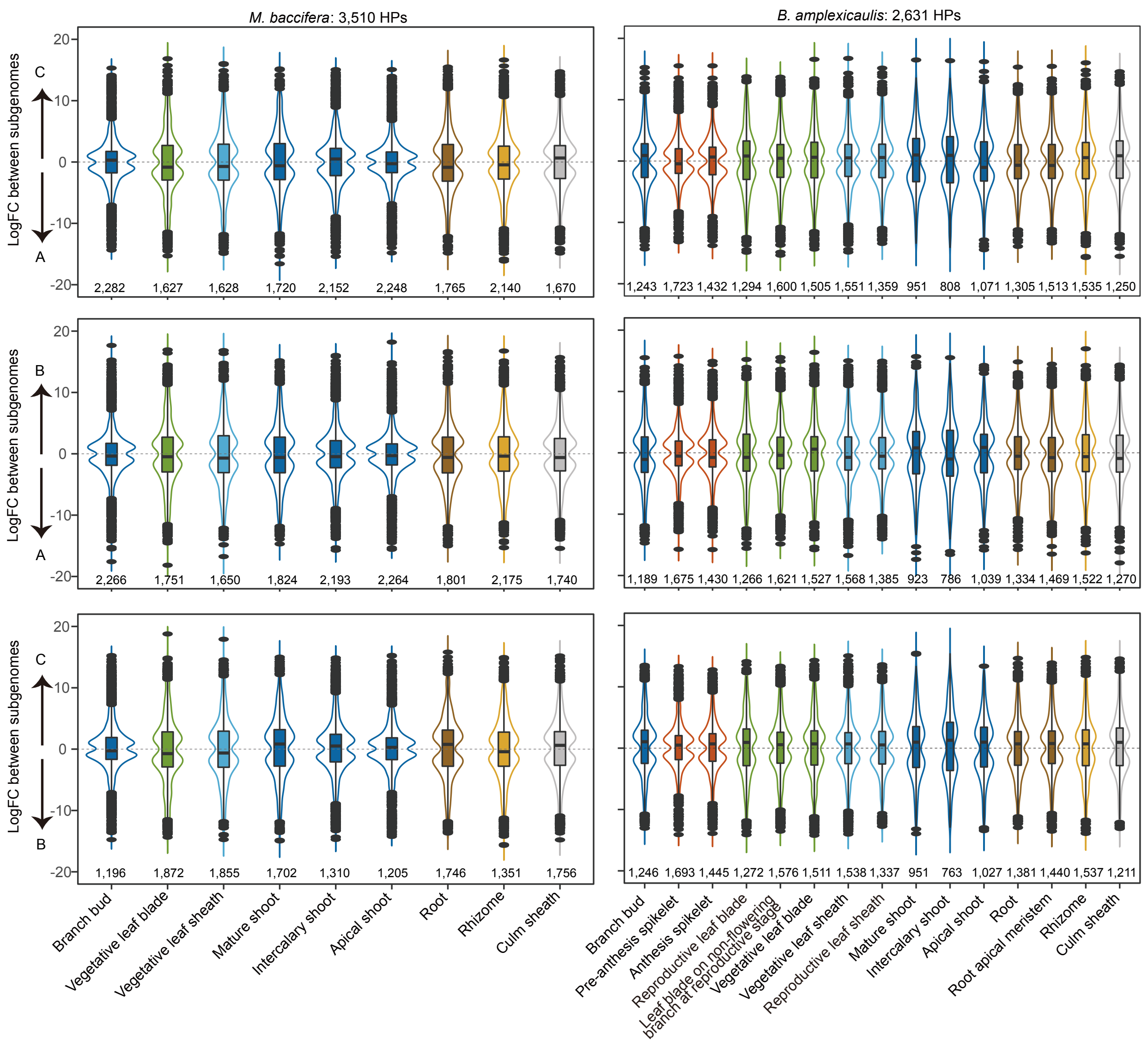

Supplementary Fig. 28. Boxplots of biased expression for homoeologous genes across different tissues in hexaploid bamboos. Log2-fold changes (FC) in biased pairwise gene expression for all homoeologous triads within each species across different tissues. Boxplots: centerline, median; box limits, first and third quartiles; whisker, 1.5x interquartile range.

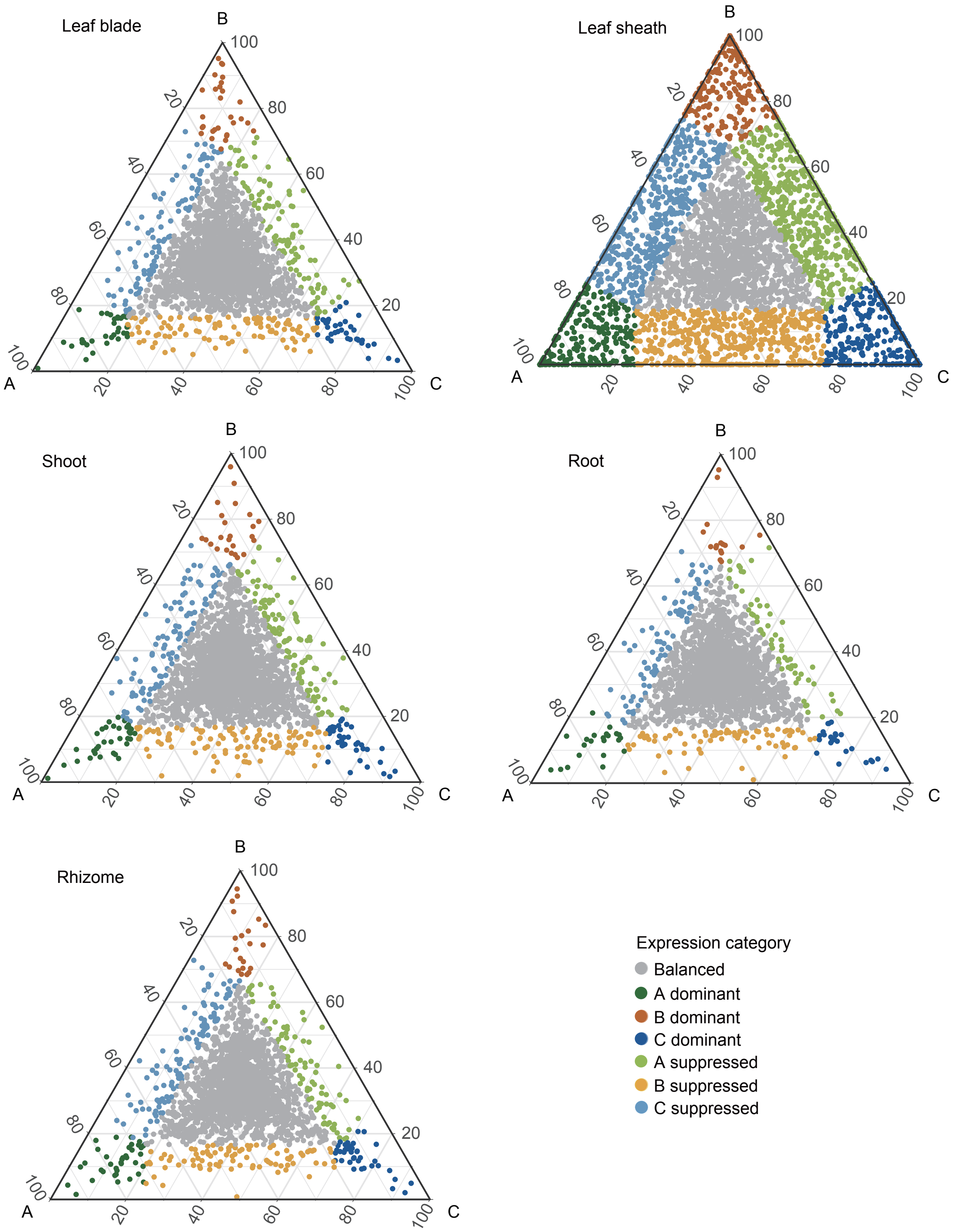

Supplementary Fig. 29. Five ternary plots showing relative expression abundance of 3,895 homoeologous triads (11,685 genes) in *D. sinicus* for each of five tissues. Triads in vertices correspond to single-subgenome-dominant categories and those close to edges and between vertices correspond to suppressed categories. Balanced triads are shown in grey.

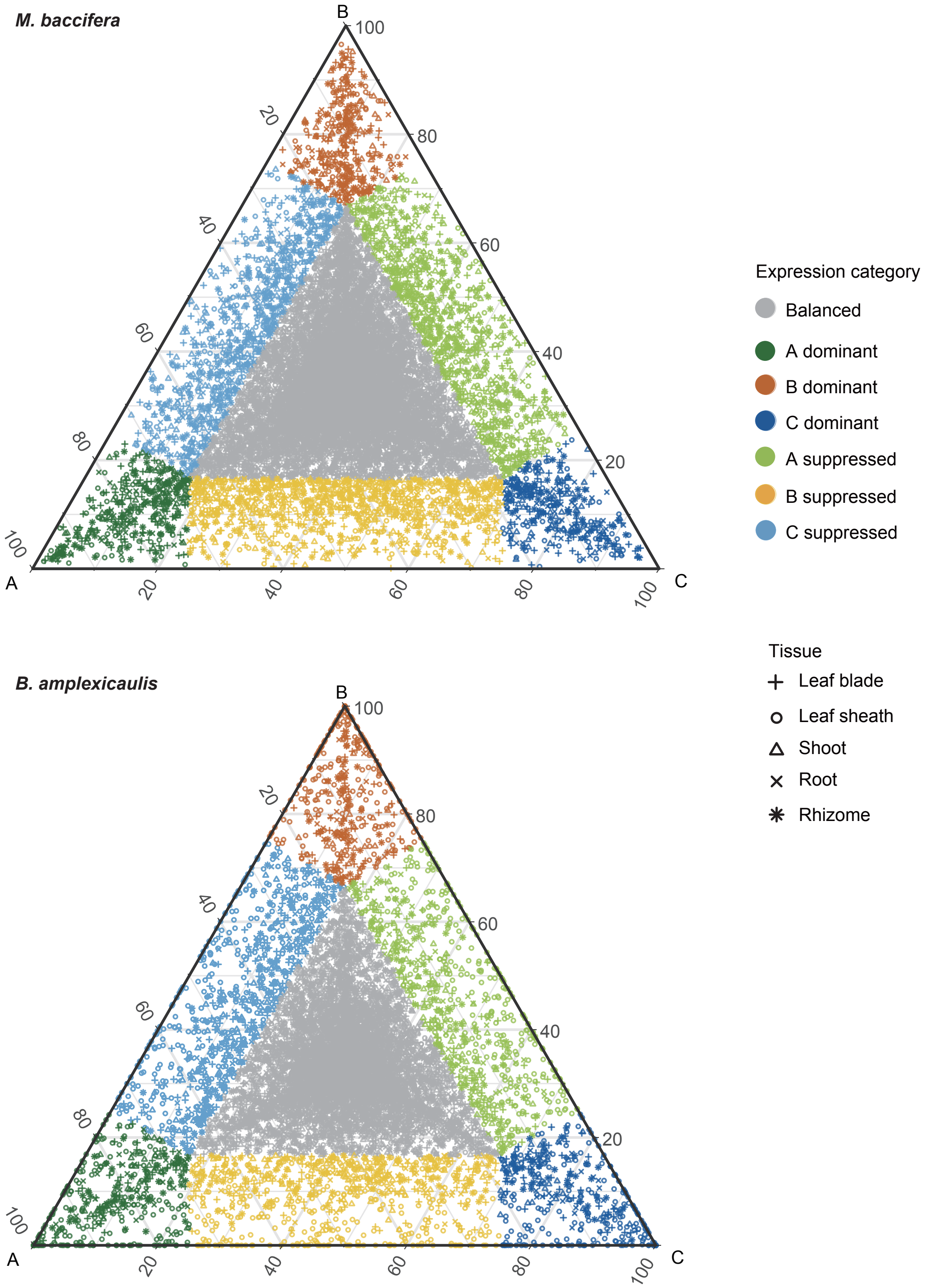

Supplementary Fig. 30. Ternary plots showing relative expression abundance for 3,510 (10,530 genes) and 2,631 (7,893 genes) homoeologous triads in *M. baccifera* and in *B. amplexicaulis*, respectively. Triads in vertices correspond to single-subgenome-dominant categories and those close to edges and between vertices correspond to suppressed categories. Balanced triads are shown in grey.

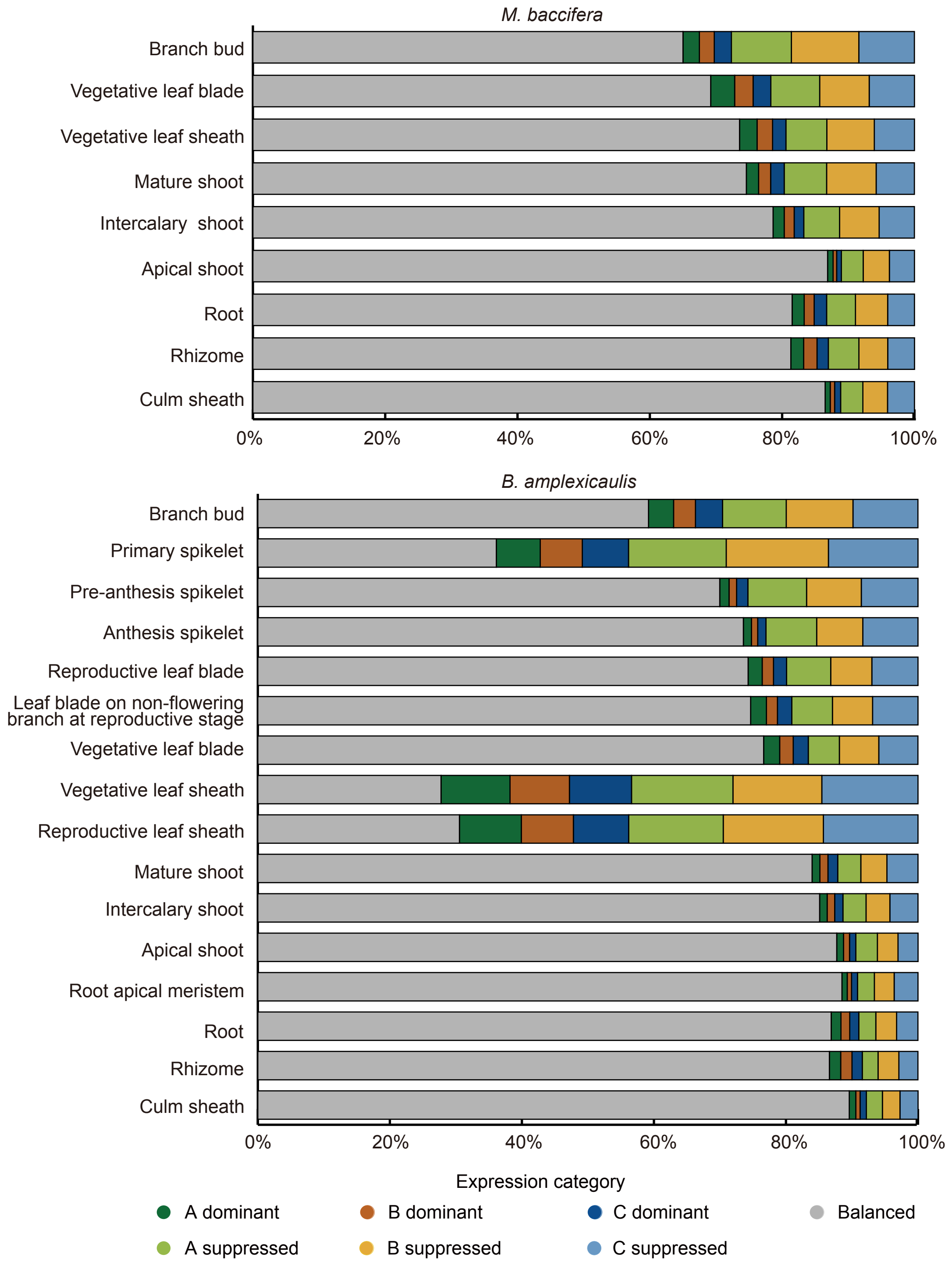

Supplementary Fig. 31. Proportion of triads in each category of homoeologous expression bias across different tissues in *M. baccifera* and *B. amplexicaulis*.

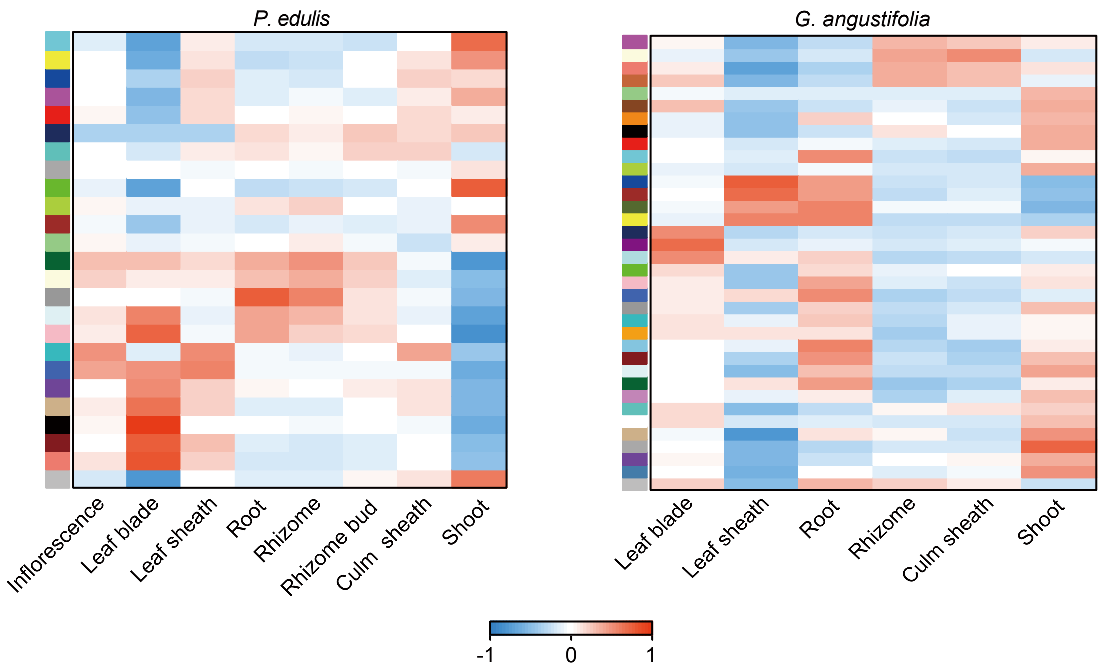

Supplementary Fig. 32. The correlation between the module eigengene (kME; representative gene expression pattern) and the tissue in the WGCNA co-expression network in *P. edulis* and *G. angustifolia*.

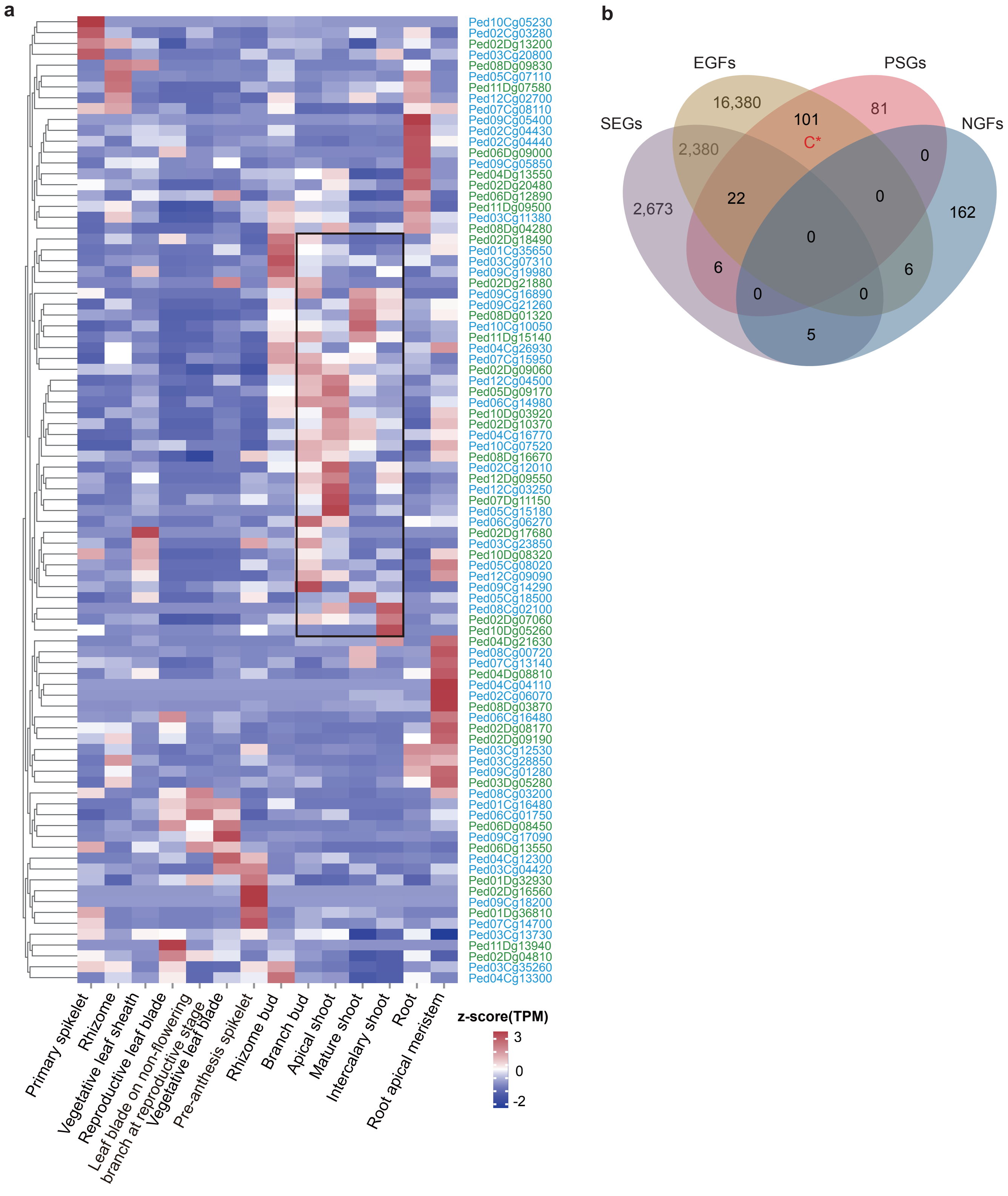

Supplementary Fig. 33. Genomic changes identified for the evolution of unique traits in woody bamboos with *P. edulis* as reference. a, Expression heatmap of new genes across different tissues. **b,** Venn diagram showing the number of positively selected genes (PSGs), new gene families (NGFs), expanded gene families (EGFs) and specific expressed genes (SEGs) in shoot or inflorescence. PSGs, NGFs and EGFs are those identified for the common ancestor node of woody bamboos.

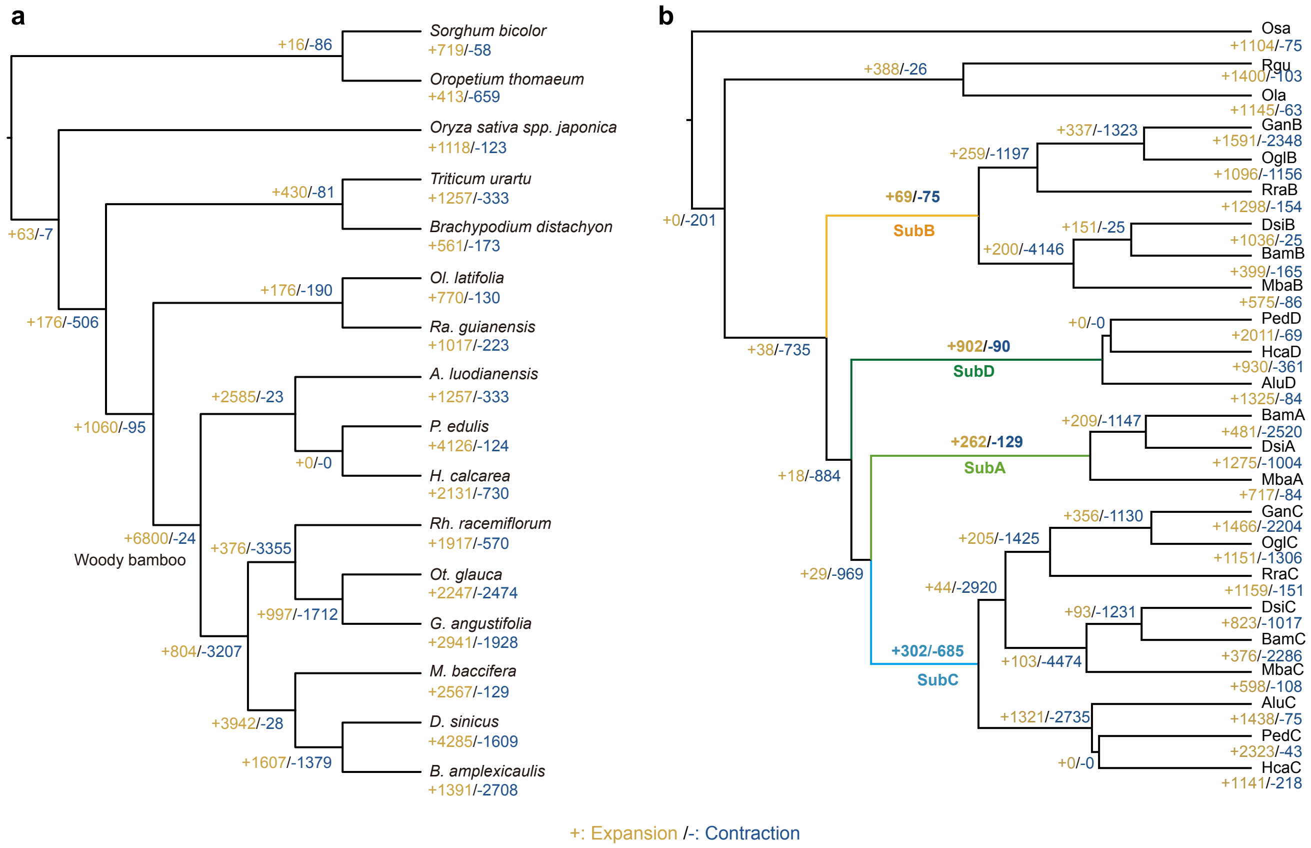

Supplementary Fig. 34. Phylogenetic tree showing significantly expanded (orange) and contracted (blue) gene families along the evolution of bamboos (a) and at the subgenome level (b).

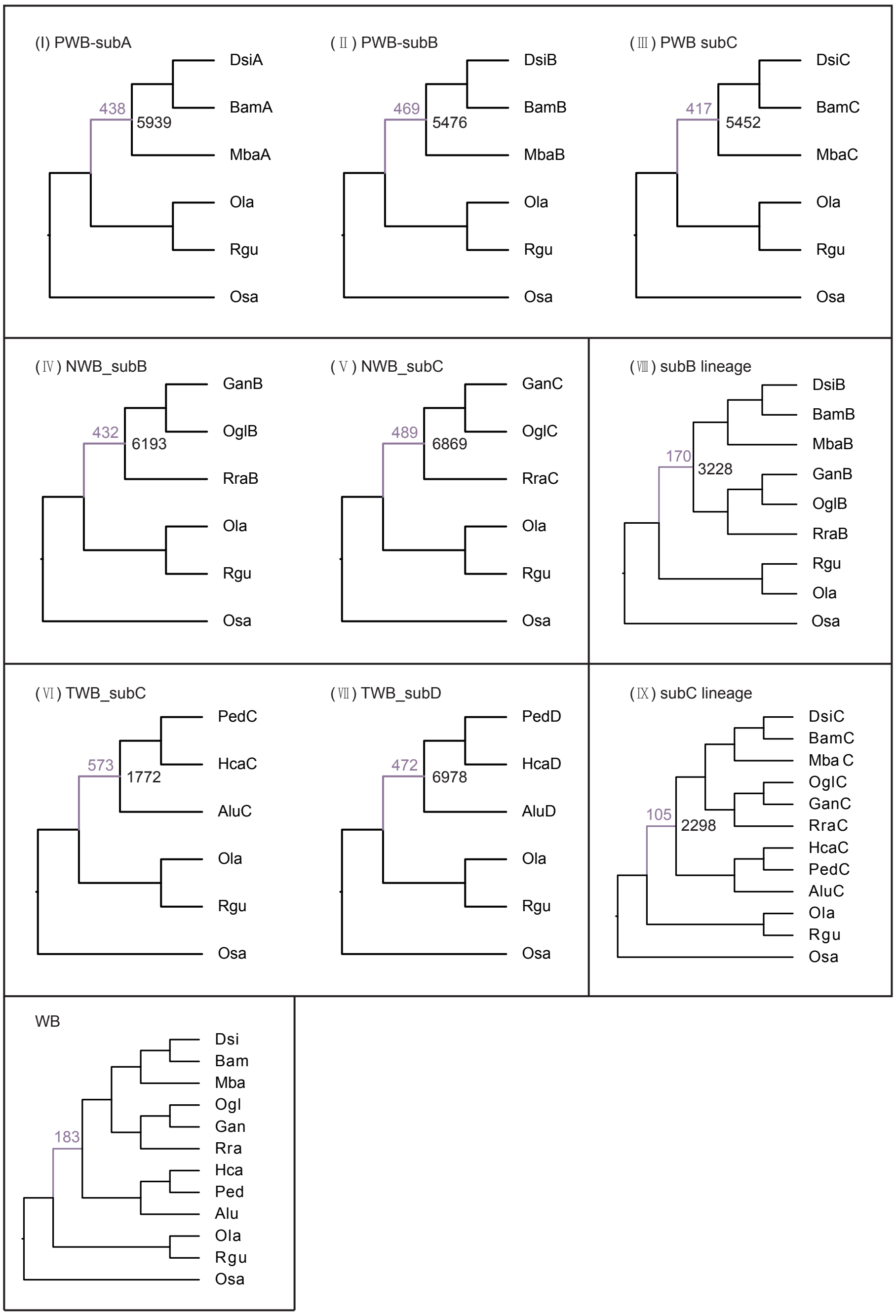

Supplementary Fig. 35. Phylogenetic tree showing the identification of positively selected genes (PSGs) at the ancestral branch of each subgenome lineage. The values beside the node indicate the numbers of single-copy genes tested in each subgenome lineage. These genes on the branch in purple are PSGs identified by PAML with the branch-site model at the *P* value cutoff of 0.05 in the Chi-square test.

Supplementary Table 1. Information of Nanopore sequencing.

Species	Total number of bases	Reads number	Mean length (bp)	N50 length (bp)
Ol. latifolia	73,352,147,900	3,740,905	19,608	25,146
Ra. guianensis	71,458,392,530	2,945,404	24,260	25,104
A. luodianensis	171,050,372,768	7,523,770	22,735	30,154
H. calcarea	215,433,156,772	8,806,594	24,462	30,657
P. edulis	230,554,164,556	11,085,976	20,796	23,742
Rh. racemiflorum	175,463,520,666	7,630,375	22,995	29,229
Ot. glauca	160,375,518,381	5,682,864	28,220	37,127
G. angustifolia	257,424,412,253	10,632,083	24,212	29,968
M. baccifera	111,804,762,060	5,097,822	21,931	29,515
B. amplexicaulis	120,010,247,941	5,094,308	23,557	30,549
D. sinicus	170,979,373,291	7,159,795	23,880	30,763

Supplementary Table 3. Gene annotation of 11 bamboo genomes.

	**Ola**	**Rgu**	**Alu**	**Hca**	**Ped**	**Rra**	**Ogl**	**Gan**	**Mba**	**Bam**	**Dsi**
Number of genes	31,189	27,496	48,870	41,907	58,664	44,113	43,141	47,971	51,908	47,213	56,847
Average gene length (bp)	4,545	4,308	4,543	4,957	4,036	4,201	4,372	4,216	4,252	4,191	4,523
Median gene length (bp)	3,701	3,279	2,976	3,124	2,525	2,656	3,098	2,812	2,950	2,920	3,120
Average CDS length (bp)	1,452	1,292	1,220	1,239	1,121	1,176	1,244	1,161	1,251	1,206	1,239
Median CDS length (bp)	1,224	1,092	1,014	1,038	891	963	1,044	942	1,035	1,005	1,032
Average exon number per gene	5.5	5.3	5.2	5.4	4.8	5.2	5.3	4.8	5.2	5.2	5.2
Median exon length (bp)	158	154	149	147	149	124	151	150	148	147	151
Average intron length (bp)	627	600	713	770	689	718	641	695	655	650	710
Average mRNA length (bp)	1,714	1,715	1,510	1,540	1,402	1,176	1,586	1,548	1,502	1,472	1,546
Median mRNA length (bp)	1,517	1,491	1,305	1,341	1,131	963	1,384	1,257	1,313	1,272	1,335

Note: Ola, *Ol. latifolia*; Rgu, *Ra. guianensis*; Alu, *A. luodianensis*; Hca, *H. calcarea*; Ped, *P. edulis*; Rra, *Rh. racemiflorum*; Ogl, *Ot. glauca*; Gan, *G. angustifolia*; Mba, *M. baccifera*; Bam, *B. amplexicaulis*; and Dsi, *D. sinicus*.

Supplementary Table 4. Repeat content of 11 bamboo genomes.

	HB (2*x*)		TWB (4*x*)			NWB (4*x*)			PWB (6*x*)		
	**Ola**	**Rgu**	**Alu**	**Hca**	**Ped**	**Rra**	**Ogl**	**Gan**	**Mba**	**Bam**	**Dsi**
LTR-RTs (Mb)	206.36	237.37	931.10	702.7	1,114.19	1,036.51	745.72	724.76	381.59	284.28	549.00
Percentage of LTR-RTs (%)	32.29	38.75	56.09	54.13	54.72	60.55	51.48	46.88	35.43	33.43	38.13
Copia (%)	8.09	11.12	10.69	21.32	13.52	12.05	16.82	12.26	13.70	14.50	13.52
Gypsy (%)	17.98	21.06	27.50	22.8	28.55	34.03	26.24	23.51	16.19	10.00	15.26
DNA TEs (Mb)	174.03	136.12	315.13	225.9	443.92	309.31	334.15	434.36	270.65	222.75	412.34
Percentage of DNA TEs (%)	27.23	22.22	18.98	17.4	21.80	18.07	23.07	28.10	25.13	26.19	28.64
Total repeat (Mb)	395.68	385.65	1,271.71	952.64	1,599.72	1,369.00	1,103.18	1,193.57	674.39	521.26	986.41
Percentage of total repeat (%)	61.90	62.95	76.61	73.39	78.56	79.93	76.16	77.21	62.62	61.30	68.51
Average repeat size (Mb)	390.67		1,274.69			1,221.92			727.35		
Average percentage of repeat (%)	62.43		76.19			77.77			64.14		

Note: Ola, *Ol. latifolia*; Rgu, *Ra. guianensis*; Alu, *A. luodianensis*; Hca, *H. calcarea*; Ped, *P. edulis*; Rra, *Rh. racemiflorum*; Ogl, *Ot. glauca*; Gan, *G. angustifolia*; Mba, *M. baccifera*; Bam, *B. amplexicaulis*; and Dsi, *D. sinicus*.

Supplementary Table 11. The proportion of putative homoeologous exchanges (HEs) between subgenomes in the woody bamboo genomes.

Species	Total gene numbers of 25,249 OGs	Number of HE genes	Proportion
A. luodianensis	45,968	265	0.576%
P. edulis	50,969	508	0.997%
H. calcarea	40,614	175	0.431%
Ot. glauca	40,911	267	0.653%
Rh. racemiflorum	40,938	334	0.816%
G. angustifolia	40,207	512	1.273%
B. amplexicaulis	42,763	250	0.585%
D. sinicus	52,814	493	0.933%
M. baccifera	48,651	432	0.888%

Supplementary Table 15. Groups of homoeologous genes in woody bamboos.

**Homoeologous group (C:D)**	***A. luodianensis***	***H. calcarea***	***P. edulis***
1:1	12,254(12,254:12,254)	9,956(9,956:9,956)	12,597(12,597:12,597)
1:N	736(736:1,576)	716(716:1,482)	891(891:2,024)
N:1	752(1,613:752)	774(1,600:774)	937(2,156:937)
Other ratios	89(196:214)	91(195:197)	155(360:357)
1:1 in microsynteny	10,929(10,929:10,929)	9,092(9,092:9,092)	10,808(10,808:10,808)
Total in microsynteny	12,650(14,344:14,249)	10,866(12,777:12,684)	12,734(14,489:14,373)
Total in homoeologeous groups	13,831(14,799:14,796)	11,537(12,467:12,409)	14,580(16,004:15,915)
%	(61.59%:64.59%)	(57.71%:63.12%)	(57.05%:60.08%)
Conserved subgenome-specific genes	(8,627:7,515)	(8,695:6,920)	(9,704:8215)
Non-conserved subgenome-specific genes	(602:596)	(440:328)	(2,335:2,359)
Total	24,028:22,907	21,602:19,657	28,053:2,6491
**Homoeologous group (B:C)**	***Rh. racemiflorum***	***G. angustifolia***	***Ot. Glauca***
1:1	9,068(9,068:9,068)	8,624(8,624:8,624)	11,539(11,539:11,539)
1:N	515(515:1,271)	578(578:1,295)	470(470:1,006)
N:1	577(1,513:577)	632(1,524:632)	455(1,012:455)
Other ratios	98(296:249)	66(171:157)	52(116:112)
1:1 in microsynteny	7,931(7,931:7,931)	7,455(7,455:7,455)	10,648(10,648:10,648)
Total in microsynteny	8,676(9,156:9,103)	8,490(9,085:9,005)	11,590(12,050:12,041)
Total in homoeologeous groups	10,258(11,392:11,165)	9,900(10,897:10,708)	12,516(13,137:13,112)
%	(52.48%:51.80%)	(50.69%:49.97%)	(64.20%:61.67%)
Conserved subgenome-specific genes	(8,614:8,872)	(8,854:9,208)	(6,843:7,713)
Non-conserved subgenome-specific genes	(1,702:1,515)	(1,748:1,512)	(482:435)
Total	21,708:21,552	21,499:21,428	20,462:21,260
**Homoeologous group (A:B:C)**	***M. baccifera***	***B. amplexicaulis***	***D. sinicus***
1:1:1	4,266(4,266:4,266:4,266)	3,236(3,236:3,236:3,236)	4,888(4,888:4,888:4,888)
1:1:N	133(133:133:275)	99(99:99:215)	201(201:201:433)
1:N:1	162(162:402:162)	74(74:192:74)	220(220:580:220)
N:1:1	198(431:198:198)	102(220:102:102)	259(565:259:259)
1:1:0	3,054(3,054:3,054:0)	2,835(2,835:2,835:0)	3,167(3,167:3,167:0)
1:0:1	2,916(2,916:0:2,916)	2,848(2,848:0:2,848)	3,002(3,002:0:3,002)
0:1:1	2,467(0:2,467:2,467)	2,169(0:2,169:2,169)	2,390(0:2,390:2,390)
Other ratios	1,055(1,418:1,362:990)	743(895:786:677)	1,325(2,242:1,241:1,266)
1:1:1 in microsynteny	3,510(3,510:3,510:3,510)	2,631(2,631:2,631:2,631)	3,895(3,895:3,895:3,895)
Total in microsynteny	11,811(10,748:10,309:11,294)	10,380(9,079:8,459:9,569)	12,307(11,736:11,097:11,973)
Total in homoeologeous groups	14,251(12,380:11,882:11,274)	12,096(10,207:9,419:9,321)	15,452(13,979:13,032:12,458)
%	68.74%:71.26%:74.03%	63.10%:64.99%:67.99%	70.01%:73.32%:77.53%
Conserved subgenome-specific genes	5,084:4,358:3,579	5,353:4,505:3,892	5,471:4,355:3,328
Non-conserved subgenome-specific genes	546:435:375	616:570:498	518:389:282
Total	18,010:16,675:15,228	16,176:14,494:13,711	19,968:17,776:16,068

Note: subgenome-specific genes are those found only in one subgenome but not its counterpart(s) within WBs while with (conserved) or without (non-conserved) homoeologs in other bamboo and five grass (rice, *Or. thomaeum*, *S. bicolor*, *T. urartu*, and *B. distachyon*) genomes.

Supplementary Table 16. Distribution of genes among subgenomes in woody bamboos.

Species	Number of genes	Number of genes in subgenomes	A (%)	B (%)	C (%)	D (%)
A. luodianensis	48,870	46,935 (96.03%)	/	/	24,028 (51.19%)	22,907 (48.81%)
H. calcarea	41,907	41,259 (98.45%)	/	/	21,602 (52.36%)	19,657 (47.64%)
P. edulis	58,664	54,544 (92.98%)	/	/	28,053 (51.43%)	26,491 (48.57%)
Rh. racemiflorum	44,113	43,690 (99.04%)	/	21,708 (50.18%)	21,552 (49.82%)	/
G. angustifolia	47,971	42,927 (84.43%)	/	21,499 (50.08%)	21,428 (49.92%)	/
Ot. glauca	43,141	41,722 (96.71%)	/	20,462 (49.04%)	21,260 (50.96%)	/
M. baccifera	51,907	49,913 (96.16%)	18,010 (36.08%)	16,675 (33.41%)	15,228 (30.51%)	/
B. amplexicaulis	47,213	44,381 (94.0%)	16,176 (36.45%)	14,494 (32.66%)	13,711 (30.89%)	/
D. sinicus	56,847	53,812 (94.66%)	19,968 (37.11%)	17,776 (33.03%)	16,068 (29.86%)	/

Supplementary Table 18. Tissue specificity of gene expression in 11 bamboo genomes.

Species	Low specificity	Moderate specificity	High specificity
Ra. guianensis	7,670(32.54%)	12,689(53.84%)	3,210(13.62%)
Ol. latifolia	7,202(31.94%)	11,445(50.76%)	3,901(17.30%)
A. luodianensis	6,333(17.55%)	21,267(58.92%)	8,493(23.53%)
H. calcarea	6,035(18.37%)	20,701(63.00%)	6,123(18.63%)
P. edulis	5,895(15.51%)	22,397(58.92%)	9,723(25.58%)
Rh. racemiflorum	10,513(27.06%)	23,557(60.64%)	4,776(12.29%)
G. angustifolia	7,572(23.52%)	18,195(56.51%)	6,433(19.98%)
Ot. glauca	8,458(24.99%)	19,402(57.32%)	5,989(17.69%)
M. baccifera	8,144(20.94%)	22,630(58.19%)	8,119(20.88%)
B. amplexicaulis	7,380(19.16%)	22,048(57.25%)	9,084(23.59%)
D. sinicus	7,566(17.27%)	26,442(60.35%)	9,808(22.38%)

Note: Genes were classified as either low specificity (Tau < 0.2), moderate specificity ($0.2 \leq$ Tau ≤ 0.8) or high specificity (Tau > 0.8).

Supplementary Table 19. The number of expressed genes in bamboo genomes and subgenomes.

Species	Number of expressed genes	Number of expressed genes in subgenomes			
		A	B	C	D
Ra. guianensis	23,428(85.21%)	/	/	/	/
Ol. latifolia	22,100(70.86%)	/	/	/	/
A. luodianensis	39,912(81.67%)	/	/	19,783(82.33%)	18,755(81.87%)
H. calcarea	35,606(84.97%)	/	/	18,335(84.88%)	16,751(85.22%)
P. edulis	41,962(71.53%)	/	/	20,690(73.75%)	19,271(72.75%)
Rh. racemiflorum	34,706(78.68%)	/	17,015(78.38%)	17,236(79.97%)	/
G. angustifolia	35,416(69.66%)	/	15,957(74.22%)	16,543(77.20%)	/
Ot. glauca	34,595(80.19%)	/	16,284(79.58%)	17,307(81.41%)	/
M. baccifera	40,327(77.69%)	13,903(77.19%)	12,971(77.79%)	12,212(80.19%)	/
B. amplexicaulis	41,494(87.89%)	14,313(88.48%)	12,616(87.04%)	12,288(89.62%)	/
D. sinicus	47,294(83.20%)	16,466(82.46%)	14,790(83.20%)	13,765(85.67%)	/

Note: The expressed genes were counted as TPM ≥ 1 in at least two RNA-seq samples.

Supplementary Table 20. Expression divergence between 1:1 or 1:1:1 homoeologous genes across five common tissues (leaf blade, leaf sheath, shoot, root, and rhizome).

Species	Observed data				Simulated data			
	Pairs	Genes	Clusters	Shifted (%)	Pairs	Genes	Clusters	Shifted (%)
A. luodianensis	4,123	8,246	10	2,617(63.47%)	1,735	3,470	10	1,087(62.65%)
H. calcarea	4,123	8,246	10	2,413(58.52%)	1,735	3,470	10	1,003(57.81%)
P. edulis	4,123	8,246	10	2,516(60.02%)	1,735	3,470	10	1,094(63.05%)
G. angustifolia	3,839	7,678	10	2,593(66.85%)	1,735	3,470	10	1,165(67.15%)
Ot. glauca	3,839	7,678	10	2,630(68.05%)	1,735	3,470	10	1,031(59.42%)
M. baccifera	1,157	3,471	10	929(84.79%)	1,157	3,471	10	929(84.79%)
B. amplexicaulis	1,157	3,471	10	991(88.9%)	1,157	3,471	10	991(88.9%)
D. sinicus	1,157	3,471	10	897(82.67%)	1,157	3,471	10	897(82.67%)

Note: Observed data are homoeologous pairs shared by all three species within each clade. The simulated data are randomly selected homoeologous genes with the number in tetraploids equal to that of hexaploids. The homoeologous genes followed in the different clusters are defined as shifted.

Supplementary Table 21. Comparison of gene expression from five tissues for all homoeologous pairs in five tetraploid genomes.

		C biased	D biased	Balanced	Biased subgenome
A. luodianensis (9,474:9,474)					
	Leaf blade	1,069	954	7,451	C
	Leaf sheath	1,128	1,079	7,267	C
	Shoot	1,712	1,639	6,123	C
	Root	2,445	2,336	4,693	C
	Rhizome	2,484	2,353	4,637	C
	Total	8,838(9.33%)	8,361(8.83%)	30,171(63.69%)	C
H. calcarea (7,808:7,808)					
	Leaf blade	1,294	1,239	5,275	C
	Leaf sheath	894	797	6,117	C
	Shoot	916	812	6,080	C
	Root	1,898	1,899	4,011	D
	Rhizome	1,772	1,713	4,323	C
	Total	6,774(17.35%)	6,460(16.55%)	25,806(66.10%)	C
P. edulis (9,306:9,306)					
	Leaf blade	2,086	1,928	5,292	C
	Leaf sheath	1,347	1,316	6,643	C
	Shoot	1,933	1,794	5,579	C
	Root	2,101	1,964	5,241	C
	Rhizome	2,371	2,277	4,658	C
	Total	9,838(21.14%)	9,279(19.94%)	27,413(58.91%)	C

		C biased	B biased	Balanced	Biased subgenome
G. angustifolia (7,455:7,455)					
	Leaf blade	1,784	1,773	3,898	C
	Leaf sheath	1,615	1,528	4,312	C
	Shoot	1,899	1,729	3,827	C
	Root	1,709	1,706	4,040	C
	Rhizome	2,137	2,140	3,178	C
	Total	9,144(24.53%)	8,876(23.81%)	19,255(51.66%)	C
Ot. glauca (10,648:10,648)					
	Leaf blade	2,392	2,245	6,011	C
	Leaf sheath	1,719	1,624	7,305	C
	Shoot	1,886	1,777	6,985	C
	Root	2,592	2,503	5,553	C
	Rhizome	2,908	2,798	4,942	C
	Total	11,497(21.59%)	10,947(20.56%)	30,796(57.84%)	C

Note: The significantly upregulated genes were defined as biased genes with the |Log2-fold changes (FC)| ≥ 1 and Benjamini-Hochberg adjusted *P* value ≤ 0.05 (two-sided Wald test) in the DeSeq2 analysis.

Supplementary Table 26. Origin and evolution of biased expressed genes in vegetative leaf blade of three woody bamboo clades.

TWB (4*x*)	Biased		Balanced	Total	NWB (4*x*)	Biased		Balanced	Total
	C	D				B	C		
Ancestor	100	75	3,948	4,123	Ancestor	223	237	3,379	3,839
A. luodianensis	373	316	3,434	4,123	*Rh. racemiflorum*	1,209	1,169	1,461	3,839
H. calcarea	671	618	2,834	4,123	*G. angustifolia*	997	1,033	1,809	3,839
P. edulis	970	965	2,188	4,123	*Ot. glauca*	887	926	2,026	3,839

PWB (6*x*)	Biased						Balanced	Total
	Dominant			Suppressed				
	A	B	C	A	B	C		
Ancestor	0	0	2	5	6	3	1,141	1,157
M. baccifera	41	34	35	67	76	72	841	1,157
B. amplexicaulis	29	23	22	60	66	69	894	1,157
D. sinicus	7	6	12	29	35	33	1,041	1,157

Supplementary Table 27. Distribution of tissue-specific expressed genes among subgenomes in *P. edulis* and *D. sinicus*.

Tissues	*P. edulis*			*D. sinicus*			
	C	D	Total	A	B	C	Total
Inflorescence	126	111	247	758	633	643	2,117
Reproductive leaf blade	188	149	359	147	118	87	371
Vegetative leaf blade	172	112	302	158	123	139	443
Leaf sheath	396	355	781	47	54	35	137
Root	196	188	410	184	157	110	476
Rhizome	186	190	402	132	119	110	383
Culm sheath	/	/	/	195	173	134	520
Shoot	2,370	2,193	4,841	1,607	1,453	1,462	4,678

Supplementary Table 29. Number of genes in different species corresponding to the 6,800 expanded gene families shared by woody bamboos.

Species	Gene number	Subgenome			
		A	B	C	D
Oropetium thomaeum	7,781	/	/	/	/
Sorghum bicolor	9,042	/	/	/	/
Brachypodium distachyon	8,754	/	/	/	/
Triticum urartu	7,280	/	/	/	/
Oryza sativa	9,386	/	/	/	/
Ra. guianensis	8,195	/	/	/	/
Ol. latifolia	8,554	/	/	/	/
A. luodianensis	18,470	/	/	8,391	8,346
H. calcarea	17,151	/	/	8,054	7,708
P. edulis	18,889	/	/	8,467	8,391
Rh. racemiflorum	15,404	/	7,587	7,669	/
G. angustifolia	16,257	/	7,430	7,570	/
Ot. glauca	17,049	/	8,158	8,417	/
M. baccifera	21,078	7,107	6,831	6,460	/
B. amplexicaulis	18,517	6,218	5,703	5,636	/
D. sinicus	22,580	7,698	7,073	6,773	/

Supplementary Table 30. The relative contribution of different duplication types for the 6,800 common expanded gene families in different species.

Species	Expanded gene number	Whole-genome duplication	Tandem duplication	Proximal duplication	Transposed duplication
A. luodianensis	18,470	14,917(80.76%)	233(1.26%)	997(5.40%)	1,148(6.22%)
H. calcarea	17,151	14,098(82.20%)	167(0.97%)	754(4.40%)	787(4.59%)
P. edulis	18,889	14,808(78.39%)	675(3.57%)	755(4.00%)	1,553(8.22%)
Rh. racemiflorum	15,404	7,085(45.99%)	426(2.77%)	329(2.14%)	392(2.54%)
G. angustifolia	16,257	10,586(65.12%)	215(1.32%)	1,113(6.85%)	1,743(1.72%)
Ot. glauca	17,049	14,311(83.94%)	514(3.01%)	374(2.19%)	812(4.76%)
M. baccifera	21,078	18,071(85.73%)	163(0.77%)	793(3.76%)	951(4.51%)
B. amplexicaulis	18,517	15,238(82.29%)	516(2.79%)	345(1.86%)	852(4.60%)
D. sinicus	22,580	19,360(85.74%)	608(2.69%)	573(2.54%)	1,164(5.16%)

Supplementary Table 33. The material content associated with lignification and growth rate of the 10th internode in *D. sinicus*.

Stage	Growth rate (cm/d)	Cellulose (%)	Hemicellulose (%)	Lignin (%)
	0.04	0.25	0.49	0.18
ST1	0.05	0.39	0.66	0.20
	0.12	0.23	0.61	0.20
Average	0.07	0.29	0.59	0.19
	0.50	0.42	1.21	0.24
ST2	0.47	0.55	1.37	0.24
	0.56	0.42	1.47	0.27
Average	0.51	0.46	1.35	0.25
	2.66	0.78	1.80	0.42
ST3	2.13	0.71	1.36	0.35
	2.60	0.77	1.87	0.58
Average	2.46	0.75	1.68	0.45
ST4	0.16	1.59	1.32	0.74
	0.16	1.04	3.32	0.85
Average	0.16	1.32	2.32	0.80

Note: We divided the whole rapid growth of shoot into four stages according to the growth curve of 10th internode, stage 1 (ST1) to stage (ST4). The content of cellulose, hemicellulose and lignin was measured using the 10th internode.
