# Supplementary material for: Genome assemblies of 11 bamboo species highlight diversification induced by dynamic subgenome dominance
Source: Nat Genet. 2024 Mar 15;56(4):710–20. doi: 10.1038/s41588-024-01683-0 (PMC11018529; doi:10.1038/s41588-024-01683-0)
Supplement: Supplementary file 2 — Reporting Summary [file 41588_2024_1683_MOESM2_ESM.pdf]

Corresponding author(s): De-Zhu Li
Last updated by author(s): Dec 28, 2023

Reporting Summary

Nature Portfolio wishes to improve the reproducibility of the work that we publish. This form provides structure for consistency and transparency in reporting. For further information on Nature Portfolio policies, see our Editorial Policies and the Editorial Policy Checklist.

Statistics

For all statistical analyses, confirm that the following items are present in the figure legend, table legend, main text, or Methods section.

n/a	Confirmed	
☐	☒	The exact sample size (*n*) for each experimental group/condition, given as a discrete number and unit of measurement
☐	☒	A statement on whether measurements were taken from distinct samples or whether the same sample was measured repeatedly
☐	☒	The statistical test(s) used AND whether they are one- or two-sided<br>*Only common tests should be described solely by name; describe more complex techniques in the Methods section.*
☒	☐	A description of all covariates tested
☐	☒	A description of any assumptions or corrections, such as tests of normality and adjustment for multiple comparisons
☐	☒	A full description of the statistical parameters including central tendency (e.g. means) or other basic estimates (e.g. regression coefficient) AND variation (e.g. standard deviation) or associated estimates of uncertainty (e.g. confidence intervals)
☐	☒	For null hypothesis testing, the test statistic (e.g. F, t, r) with confidence intervals, effect sizes, degrees of freedom and P value noted<br>*Give P values as exact values whenever suitable.*
☒	☐	For Bayesian analysis, information on the choice of priors and Markov chain Monte Carlo settings
☒	☐	For hierarchical and complex designs, identification of the appropriate level for tests and full reporting of outcomes
☒	☐	Estimates of effect sizes (e.g. Cohen's *d*, Pearson's *r*), indicating how they were calculated

Our web collection on statistics for biologists contains articles on many of the points above.

Software and code

Policy information about availability of computer code

Data collection	No software was used for data collection
Data analysis	Genome size and heterozygosity estimation: GenomeScope (https://github.com/schatzlab/genomescope); De novo genome assembly: CANU (v1.7), SMARTdenovo (v1.0.0) (https://github.com/ruanjue/smartdenovo), NextDenovo (v2.3.1) (https://github.com/Nextomics/NextDenovo), Racon (v1.4.21), Nextpolish (v1.3.0), Pilon (v1.23), BWA (v0.7.10-r789), Bowtie2 (v2.3.2), HiC-Pro (v.2.8.1), LACHESIS (https://github.com/shendurelab/LACHESIS); Assembly quality evaluation: BWA (v0.7.10-r789), LTR_retriever (v2.6), Mabs (v2.19) (https://github.com/shelkmike/Mabs); TE annotations: EDTA (v1.8.5), LTR_Finder (v1.07), LTR_retriever (v2.6), Generic Repeat Finder (v1.0), TIR-Learner (v1.19), HelitronScanner (v1.1), RepeatModeler (v2.0.1) (https://github.com/Dfam-consortium/RepeatModeler); Gene annotations: Genscan, Augustus (v2.4), GlimmerHMM (v3.0.4), GeneID (v1.4), SNAP (v2006.07.28), GeMoMa (v1.3.1), HISAT (v2.0.4), Stringtie (v1.2.3), TransDecoder (v2.0) (https://github.com/TransDecoder/TransDecoder), GeneMarkS-T (v5.1), EvidenceModeler (v1.1.1), PASA (v2.0.2), BUSCO (v4.0.6); Methylation analysis: Bismark (v0.21.0), ViewBS (v0.1.9) (https://github.com/xie186/ViewBS); Subgenome identification: jcvi (v1.1.17), MAFFT (v7.471), PAL2NAL v14 (https://www.bork.embl.de/pal2nal/), RAxML (v8.2.12), OrthoFinder (v2.3.12), Identity (v1.0) (https://github.com/BioinformaticsToolsmith/Identity); Phylogenetic analysis and divergence time estimation: Newick utilities (v1.6.0) (https://github.com/tjunier/newick_utils), ASTRAL (v5.6.3), ipyrad (v0.9.74) (https://ipyrad.readthedocs.io/en/master/), MEGA-X, phyparts (v0.0.1) (https://bitbucket.org/blackrim/phyparts/src/master/), GetOrganelle (v1.7.1), MAFFT (v7.471), trimAl (v1.4), RAxML (v8.2.12), MCMCTREE in the PAML (v4.9); ILS and hybridization analysis: PhyloNet (v3.8.0), QuIBL (https://github.com/miriammiyagi/QuIBL), HyDe (v0.4.3) (https://github.com/pblischak/HyDe); Ancestral karyotype reconstruction: MCScanX (https://github.com/wyp1125/MCScanX), IAGS (https://github.com/xjtu-omics/IAGS); Identification of chromosomal rearrangements and inversions: jcvi (v1.1.17), MUMmer (v4.00rcl), bedtools (v2.30.0), EMBOSS-6.6.0, SyRI (v1.5), SURVIVOR (v1.0.7), OrthoFinder (v2.5.2); Gene retention evaluation: CoGe's SynMap2 (https://genomevolution.org/coge/); Inference of gene families and homoeologous groups: CAFÉ (v4.2.1) (https://github.com/hahnlab/CAFE.git), DIAMOND (v2.1.8) (https://github.com/bbuchfink/diamond), OrthoFinder (v2.5.2), Bowtie2 (v2.3.4.1), MCScanX (https://github.com/wyp1125/MCScanX), r8s (v1.8.1), (http://

ceiba.biosci.arizona.edu/r8s/r8s1.81.tar.gz), DupGen_finder (https://github.com/qiao-xin/DupGen_finder); Transcriptome analyses: FastQC (v0.11.8), Fastp (0.20.1), HISAT2 (v2.1.0), SAMtools (v1.10), StringTie (v1.3.4d) (http://ccb.jhu.edu/software/stringtie), DESeq2 (v1.14.1), R (v4.0.3), tispec R-package (https://rdrr.io/github/roonysgalbi/tispec); Expression divergence between subgenomes: R (v4.1.2); Expression bias between subgenomes: DESeq2 (v1.14.1), Co-expression analysis and hub genes: WGCNA (v1.69) (https://horvath.genetics.ucla.edu/html/CoexpressionNetwork/Rpackages/WGCNA/); Identifying new genes, PSGs and tissue-specific expressed genes: "myTAI" R package (v0.9.3) (https://github.com/drostlab/myTAI), PAML package (v4.8), DESeq2 (v1.14.1), KaKs-Calculator (v2.0), PAL2NAL v14, OrthoFinder (v2.5.2), MAFFT (v7.475), ParaAT (v2.0); Identification of lignin genes and their expression: OrthoFinder (v2.5.2), MAFFT (v7.475), IQ-TREE2 (v2.0.3), DESeq2 (v1.14.1), STEM (v1.3.13); Reference for all software have been described in the Methods. The custom codes included in this study are available at GitHub (https://github.com/yunlongliukm/BGSP). Codes are also archived at Zenodo (https://doi.org/10.5281/zenodo.10146649).

For manuscripts utilizing custom algorithms or software that are central to the research but not yet described in published literature, software must be made available to editors and reviewers. We strongly encourage code deposition in a community repository (e.g. GitHub). See the Nature Portfolio guidelines for submitting code & software for further information.

Data

Policy information about availability of data

All manuscripts must include a data availability statement. This statement should provide the following information, where applicable:

- Accession codes, unique identifiers, or web links for publicly available datasets
- A description of any restrictions on data availability
- For clinical datasets or third party data, please ensure that the statement adheres to our policy

The 11 bamboo genome assemblies (GenBank numbers: JAYEVB000000000, JAYEVC000000000, JAYEVD000000000, JAYEVE000000000, JAYEVF000000000, JAYEVG000000000, JAYEVH000000000, JAYEVI000000000, JAYEVJ000000000, JAYEVK000000000 and JAYGGG000000000), raw sequencing data and RNA-seq data are available at NCBI (accession: PRJNA948693). Genomes and annotations can be accessed at CoGe (https://genomevolution.org/coge/NotebookView.pl?nid=3091) and the bamboo genomic resource website (http://bamboo.genobank.org/). Functional annotation of the genomes used the Poales_odb10 database (https://busco-data.ezlab.org/v5/data/lineages/poales_odb10.2020-08-05.tar.gz). Gene family inferences used the KEGG (https://www.genome.jp/kegg/kegg2.html) and GO (https://geneontology.org/) databases.

Human research participants

Policy information about studies involving human research participants and Sex and Gender in Research.

Reporting on sex and gender	N/A
Population characteristics	N/A
Recruitment	N/A
Ethics oversight	N/A

Note that full information on the approval of the study protocol must also be provided in the manuscript.

Field-specific reporting

Please select the one below that is the best fit for your research. If you are not sure, read the appropriate sections before making your selection.

☒ Life sciences ☐ Behavioural & social sciences ☐ Ecological, evolutionary & environmental sciences

For a reference copy of the document with all sections, see nature.com/documents/nr-reporting-summary-flat.pdf

Life sciences study design

All studies must disclose on these points even when the disclosure is negative.

Sample size	No statistical methods were required to establish sample size for this study. To cover different ploidal levels and genome diversity, 11 representative bamboo species were chosen for genome sequencing. Four bamboo species (Raddia guianensis, Phyllostachys edulis, Guadua angustifolia, and Dendrocalamus sinicus) representing herbaceous bamboos (HBs), temperate woody bamboos (TWBs), neotropical woody bamboos (NWBs) and paleotropical woody bamboos (PWBs) for whole-genome bisulfite sequencing (WGBS). A total of 476 transcriptome samples representing different tissues at various developmental stages across the 11 sequenced bamboos were sampled for RNA extraction and transcriptome sequencing, mostly with three biological replications per tissue per species.
Data exclusions	Raw sequence data were quality filtered as described in the manuscript. In the phylogenetic analysis, we removed 26 genes from the 456 "perfect-copy" syntenic gene data set and 654 genes from the 2675 "perfect-copy" syntenic gene data set based on the criteria as described in the Methods, respectively.
Replication	At least two biological replicates and three for the most were collected for each tissue type of RNA-seq. Two biological replicates were

Replication	collected for each leaf tissue sample for whole-genome bisulfite sequencing (WGBS). The experiment of anatomical observation of shoot in Dendrocalamus sinicus was repeated independently three times. Bootstrapping for phylogenetic analyses based on "perfect-copy" syntenic genes from 11 bamboo species and rice genome were replicated 200 times, while bootstrapping for phylogenetic analyses based on plastid genome sequences were replicated 1000 times. Two parallel runs were performed in the analyses of divergence time estimation. All attempts at replication were successful.
Randomization	Genomic analyses were conducted in a non-randomized order as we do not expect batch variations.
Blinding	Group allocation was not relevant to this study, so blinding was not necessary.

Reporting for specific materials, systems and methods

We require information from authors about some types of materials, experimental systems and methods used in many studies. Here, indicate whether each material, system or method listed is relevant to your study. If you are not sure if a list item applies to your research, read the appropriate section before selecting a response.

Materials & experimental systems

n/a	Involved in the study
☒	☐ Antibodies
☒	☐ Eukaryotic cell lines
☒	☐ Palaeontology and archaeology
☒	☐ Animals and other organisms
☒	☐ Clinical data
☒	☐ Dual use research of concern

Methods

n/a	Involved in the study
☒	☐ ChIP-seq
☒	☐ Flow cytometry
☒	☐ MRI-based neuroimaging
